# Eunuchs or Females? Causes and Consequences of Gynodioecy on Morphology, Ploidy, and Ecology of *Stellaria graminea* L. (Caryophyllaceae)

**DOI:** 10.3389/fpls.2021.589093

**Published:** 2021-04-12

**Authors:** Jaromír Kučera, Marek Svitok, Eliška Gbúrová Štubňová, Lenka Mártonfiová, Clément Lafon Placette, Marek Slovák

**Affiliations:** ^1^Plant Science and Biodiversity Centre, Institute of Botany, Slovak Academy of Sciences, Bratislava, Slovakia; ^2^Faculty of Ecology and Environmental Sciences, Technical University in Zvolen, Zvolen, Slovakia; ^3^Department of Ecosystem Biology, Faculty of Science, University of South Bohemia in České Budějovice, České Budějovice, Czechia; ^4^Slovak National Museum, Natural History Museum, Bratislava, Slovakia; ^5^Botanical Garden, P. J. Šafárik University, Košice, Slovakia; ^6^Department of Botany, Charles University, Prague, Czechia

**Keywords:** Carpathians, ecological drivers, gynodioecy, sexual polymorphism, *Stellaria graminea*, whole genome duplication

## Abstract

Plant speciation results from intricate processes such as polyploidization, reproductive strategy shifts and adaptation. These evolutionary processes often co-occur, blurring their respective contributions and interactions in the speciation continuum. Here, relying on a large-scale study, we tested whether gynodioecy triggers the divergent evolution of flower morphology and genome between sexes, and contributes to the establishment of polyploids and colonization of ecological niches in *Stellaria graminea*. We found that gynodioecy in *S. graminea* leads to flower morphology divergence between females and hermaphrodites, likely due to sexual selection. Contrary to our expectations, gynodioecy occurs evenly in diploids and tetraploids, suggesting that this reproductive strategy was not involved in the establishment of polyploids. Both diploid and tetraploid females have a larger genome size than hermaphrodites, suggesting the presence of sex chromosomes. Finally, ecology differs between cytotypes and to a lesser extent between sexes, suggesting that the link between environment and presence of females is indirect and likely explained by other aspects of the species’ life history. Our study shows that gynodioecy leads to the consistent evolution of sexual traits across a wide range of populations, cytotypes and environments within a given species, and this likely contributes to the phenotypic and genetic distinctiveness of the species from its sister clades.

## Introduction

Sexual polymorphisms in flowering plants have been of fundamental interest to evolutionary biologists since Darwin’s era (e.g., Darwin, 1877; Richards, 1997; Geber et al., 1999; Renner, 2014 for review). While the majority of angiosperms are hermaphrodites, the recurrent evolution of sexual polymorphisms in reproduction systems has occurred in various vascular plant lineages (e.g. Richards, 1997; Barrett, 2002; Barrett and Hough, 2013; Bachtrog et al., 2014; Renner, 2014; Käfer et al., 2017). Gynodioecy is one of the sexual polymorphisms characterized by the coexistence of two genetically determined sexual morphs, hermaphrodites and females (Darwin, 1877; Charlesworth and Charlesworth, 1978; Delph et al., 2007; McCauley and Bailey, 2009). This phenomenon is uncommon across angiosperms, but evolved convergently in numerous lineages (≪1%; Godin and Demyanova, 2013; Caruso et al., 2016; Rivkin et al., 2016).

Gynodioecy is considered to be an intermediate step in the transition between hermaphroditism and dioecy (Charlesworth and Charlesworth, 1978; Van de Paer et al., 2015). However, it remains unclear whether dioecy and gynodioecy share the same genetic basis, and to which extent this depends on the species. Indeed, on one hand, the genetic basis of gynodioecy was shown to be predominantly determined by the interaction of cytoplasm and nuclear restorer genes, a process called cytoplasmic male sterility (CMS) (Frank, 1989; Schnable and Wise, 1998; Wise and Pring, 2002). This situation differs to dioecy, which is considered to be determined by nuclear genes (Charlesworth and Charlesworth, 1978; Charlesworth, 2002). On the other hand, the same sex chromosome was found to cause dioecy and gynodioecy in *Papaya* species (Yu et al., 2008), suggesting that a common genetic basis may facilitate the evolutionary transition from hermaphroditism to dioecy *via* gynodioecy. This exciting perspective nevertheless requires additional investigation.

The genetic factors determining gynodioecy are also difficult to disentangle from various intrinsic but also environmental factors (Caruso et al., 2016). For example, stressful or extreme environments may favor the presence of one sex or the other, for example, stressful or extreme environments may favor the presence of one sex or the other, leading to the diversification of niches between females and hermaphrodites in gynodioecious plants (Alonso and Herrera, 2001; Vaughton and Ramsey, 2005; Caruso and Case, 2007; Ruffatto et al., 2015; Abdusalam et al., 2017). Also, meta-analytical studies indicated that sexually polymorphic species are more likely to be herbs than trees, and more frequently occupy the temperate zone than (sub)tropical regions (Caruso et al., 2016; Rivkin et al., 2016). It, however, remains unclear whether the expression of sex is plastic and influenced by environmental factors, as previously shown (Vaughton and Ramsey, 2012; Buide et al., 2018), or whether gynodioecy is associated with other life-history/adaptive traits, or demographic/evolutionary processes, such as polyploidy, that are also linked to environmental factors. The link between ecological factors and gynodioecy is especially important to consider in polyploid species, as it has been shown that polyploid cytotypes often undergo shifts in their ecological niches compared to their diploid counterparts (Wei et al., 2019).

Indeed, whole genome duplication (WGD) is a mechanism playing one of the most remarkable roles in vascular plant evolution (Ramsey and Schemske, 1998; Miller and Venable, 2000; Ashman et al., 2013; Glick et al., 2016). Polyploid lineages are often found in more extreme environments than their diploid counterparts (e.g., Van de Peer et al., 2009; Wei et al., 2019), and gynodioecy may play a role in the establishment of polyploids in such environments via several mechanisms. For example, it was shown that in challenging conditions, the seeds and progeny from females show higher fitness than those from hermaphrodite parents (Spigler and Ashman, 2011; Dalton et al., 2013). This is one of the possible scenarios explaining why sexual polymorphism is often found more frequently in polyploid compared to diploid species (Ashman et al., 2013; Wang et al., 2019). In particular, gynodioecy was found to be strongly associated with polyploidy in *Lycium californicum* (Blank et al., 2014; Miller et al., 2016). Nevertheless, the positive association between sexual polymorphism and WGD is not a flawless rule. Indeed, the opposite transitions from sexually monomorphic polyploids toward sexually polymorphic diploids, or the co-occurrence of sexual dimorphisms in both diploids and polyploids, can also be found (Miller and Venable, 2000; Ashman et al., 2013; Blank et al., 2014). The link between WGD and gynodioecy therefore remains unclear (Miller and Venable, 2000; Pannell et al., 2004; Ashman et al., 2013; Glick et al., 2016), and requires further investigation.

Whole genome duplication is often accompanied by important phenotypic changes (e.g., Otto, 2007; Van de Peer et al., 2009). On the other hand, gynodioecy may provide the possibility to reallocate resources to more specialized female functions, including seed production. This is usually accompanied by other changes such as the size of floral organs. In most gynodioecious species females bear smaller flowers than hermaphrodites (e.g., Delph et al., 1996; Shykoff et al., 2003; Miller et al., 2016; Kamath et al., 2017). This is likely attributable to Bateman’s principle (Bateman, 1948), i.e., male reproductive success is often pollinator-limited, exerting a stronger selective pressure on the male function to attract pollinators compared to the female function, which explains why larger flower size is mostly driven by the male function (Paterno et al., 2020). Therefore, systems showing variability both in ploidy and reproductive strategies represent an opportunity to evaluate the relative impact of each process and their interaction on floral evolution.

We focus here on *Stellaria graminea (Caryophyllaceae)*, a perennial and predominantly autogamous herb which often spreads clonally and occupies open, native or man-made habitats from sea level to mountainous zones in Eurasia. It also non-indigenously occurs in Northern America and New Zealand (e.g., Gadella, 1977; Kurtto, 2001; Morton, 2005; Slovák et al., 2012). *Stellaria graminea* is a diploid-tetraploid species complex (2*n* = 2*x* = 26 and 2*n* = 4*x* = 52), but apart from this, the odd ploidy triploid cytotype (2*n* = 3*x* = 39) has been recurrently reported (Gadella, 1977; Harmaja, 1992; Kurtto, 2001; Morton, 2005; Goldblatt and Lowry, 2011; Rice et al., 2015). Most *S. graminea* plants bear perfect, self-compatible flowers, but male-sterile individuals are often reported (Horne, 1914; Gadella, 1977; Chater and Heywood, 1993; Kurtto, 2001; Morton, 2005; Slovák et al., 2012). Several authors thus consider the species gynodioecious (Kurtto, 2001; Morton, 2005; Slovák et al., 2012), though without investigating whether gynodioecious populations are diploid, polyploid or both. *Stellaria graminea* thus appears to be a good model for understanding the association between sexual polymorphism and WGD and its impact on reproduction evolution. While gynodioecy has been proposed to be ancestral in Caryophyllaceae (Desfeux et al., 1996), it is only reported in 12.4% of *Stellaria* species (Godin, 2020). It therefore suggests that gynodioecy has been lost several times in *Stellaria*, and this loss might be ploidy-dependent, given the possible advantages gynodioecy may provide to polyploids, as explained above. True gynodioecy and associated male sterility should, however, not be confused with the effects of heteroploid hybridization, which also leads to male infertility (e.g., Soltis and Soltis, 2009; Kolář et al., 2017). In mixed ploidy and sexually dimorphic systems such as the genus *Stellaria* L., heteroploid hybrids might have been mistaken for female individuals (Chinnappa, 1985; Macdonald et al., 1988; Kurtto, 2001; Chinnappa et al., 2005; Morton, 2005; Dang and Chinnappa, 2007). In contrast, other authors suggest that plants with imperfect floral architecture are fully sterile triploid hybrids between a diploid and a tetraploid parent (Gadella, 1977; Morton, 2005). Nevertheless, fully fertile *S. graminea* with a chromosome number close to triploid level were reported from Finland (Harmaja, 1992). Thus, the characterization of *S. graminea* morphs with imperfect floral morphology is undecided. It is therefore important to determine whether the male-sterile plants are fully sterile triploid hybrids or whether the species is gynodioecious, with interbreeding female and perfect-flowered hermaphrodites within populations. Likewise, the male-sterile plants should be characterized for their morphological and ecological specificities.

Employing an extensive population sampling from the Carpathian region and a combination of karyological, cytogenetic, morphometric, pollen, and ecological analyses, we investigated the occurrence of gynodioecy in *S. graminea*, and compared the floral morphology, genome size and ecology of plants bearing female and hermaphrodite flowers. We found that (1) *S. graminea* sexual morphs with imperfect flowers and reduced petal size are true fertile gynodioecious females and not sterile triploids, showing that the *Stellaria* genus is prone to gynodioecy; (2) True gynodioecy exists both in diploid and tetraploid *S. graminea*, suggesting that WGD did not play any role in the maintenance of gynodioecy or *vice versa* in this species; (3) Genome size differences between females and hermaphrodites suggest the presence of sex chromosomes both in diploids and tetraploids; (4) Floral morphology divergence was mostly observed between females and hermaphrodites, and to a lesser extent between diploids and tetraploids, suggesting a stronger role of sexual selection than WGD in floral evolution; (5) The presence of gynodioecy, but also of different cytotypes, is correlated with specific abiotic conditions at the broad geographic scale, making it difficult to disentangle a physiological link between environment and gynodioecy (plasticity) from a broad association with other aspects of the life history of *S. graminea*. Thanks to a broad geographical study spanning a whole biogeographical region (the Carpathians), our study provides important insights on the evolution of gynodioecy.

## Materials and Methods

### Sampling Design and Studied Area

We investigated variation in sexual system and ploidy of *S. graminea* across the entire Carpathian region (see Hurdu et al., 2016; Kliment et al., 2016), where the species is common and abundant (Slovák et al., 2012). The Carpathian region, a component of the European Alpine System (Ozenda, 1985), is known for its huge geomorphological, environmental and biological diversity (e.g., Pawłowski, 1970; Hurdu et al., 2016; Mráz et al., 2016) and thus provides a unique opportunity to explore various aspects of plant evolution including diversification of sexual system and WGD.

**FIGURE 1:**
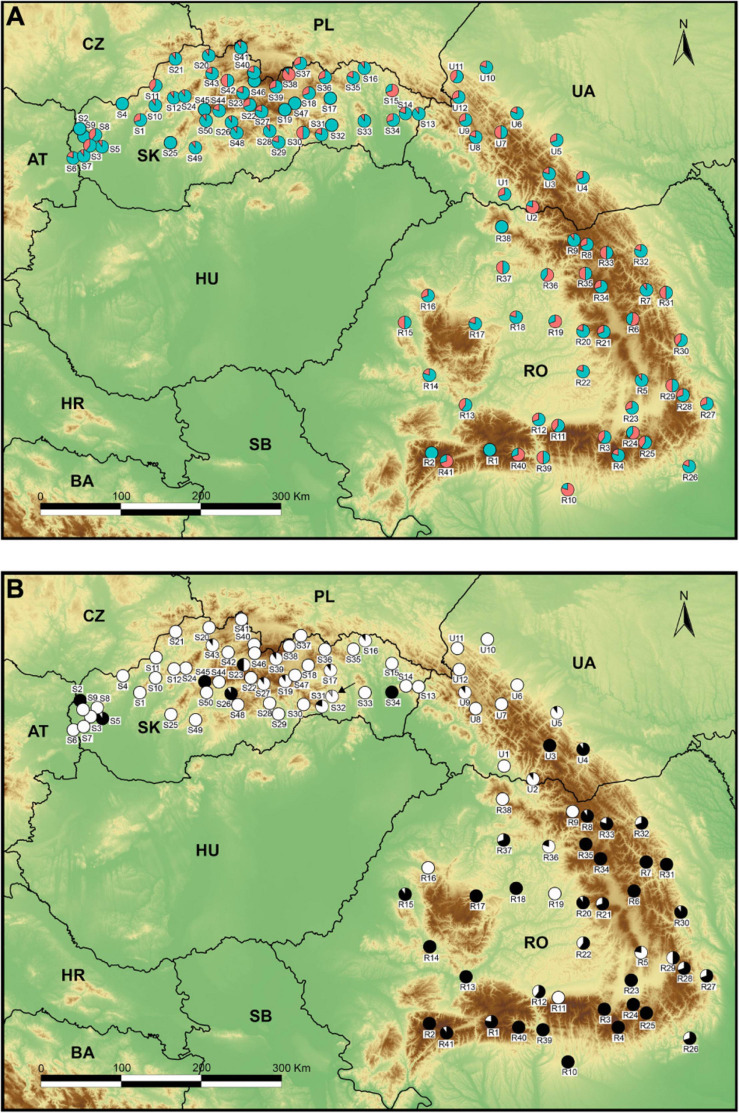
Map of 103 sample sites of *Stellaria graminea* used in the study. **(A)** Frequency and proportion of sexual morphs detected within the study: the hermaphrodite morph – cyan (*n* = 712) and the male-sterile morph – red (*n* = 290). Population codes (white squares) follow Supplementary Table 1. **(B)** Frequency and proportion of cytotypes detected within the study: diploid – black (*n* = 375), tetraploid – white (n = 626), gray – triploid (*n* = 1, marked also with a black arrow).

We investigated the spatial pattern of sexual polymorphism and ploidy level of 1002 individuals from 103 populations throughout the Carpathian region (50 from Slovakia, 12 from Ukraine, and 41 from Romania, Figure 1 and Supplementary Table 1). Populations represented the entire ecological and altitudinal range of the species. Nevertheless, we did not identify significant variability in the bedrock preference of the species, the vast majority of populations occupy habitats with non-alkaline soils (cf. Slovák et al., 2012). Vegetative reproduction and clonal growth are known for this species (e.g., Gadella, 1977; Kurtto, 2001; Morton, 2005; Slovák et al., 2012). We collected ten randomly selected individuals per population, with a minimum distance of 50 m from one another to minimize the collection of potential clones.

### Sexual Morph Identification

**FIGURE 2:**
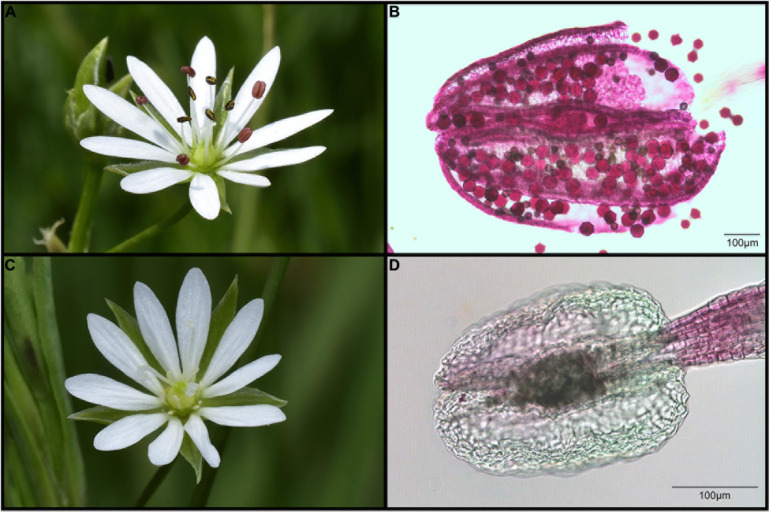
Flower structure in the diploid cytotype of *Stellaria graminea* (locality SK-ST2). **(A)** Hermaphrodite morph with fully developed stamens. **(B)** Microphotograph of well-developed anthers of the hermaphrodite morph, with viable pollen grains. Pollen viability was determined using modified Alexander staining. **(C)** The male-sterile morph with undeveloped stamens. **(D)** Microphotograph of vestigial anthers of the male-sterile morph, with no pollen grains.

We determined the sex of each plant by examining every open flower, mature bud and developing fruit in the field, and on collected flowers and herbarium material, for the presence and development of sexual organs. Plants were considered hermaphrodite if every flower had well-developed anthers with visible pollen (Figures 2A,B), and well-developed seeds could be found within the developing fruits. Plants were considered female if every flower had vestigial anthers (Figures 2C,D) and developing fruits had well-developed seeds. For a subset of samples (621 individuals out of 1002), we performed a pollen viability assay and confirmed that the vestigial anthers were sterile, as all females identified as such did not bear any pollen grains. We analyzed only plants for which we could unambiguously determine sexual morphs and ploidy level (Supplementary Table 2). Twenty-eight individuals in which it was not possible were excluded [17 individuals excluded for unreliable relative DNA content (RGS) result and 11 excluded due to impossible sex assignment] from the final dataset (thus *n* = 1002 instead of 1,030).

### Pollen Viability Estimation and Seed Production

Pollen viability was determined using a modified Alexander’s stain, following the protocol of Peterson et al. (2010). The discrimination between viable and non-viable grains was evaluated based on their shape, size and the color of their staining. Well-stained magenta-red pollen grains of regular shape were considered viable, while bluish-green or greenish-purple and shrunken grains were considered aborted and non-viable (see Figures 2A–D and Peterson et al., 2010). At least 100 grains per flower were observed. The pollen quality was estimated in 621 individuals (one flower per plant) and was expressed as the percentage of viable pollen grains per 100 screened pollen grains (Supplementary Table 2). Its variation between and within both cytotypes was calculated using univariate statistics parameters (median, 10, 25, 75, and 90th percentile as well as the extreme values) and visualized by box-plot graphs (Supplementary Figure 1).

Fruits were available on 760 of the plants and we checked the capsules for the presence of well-developed seeds (Supplementary Table 2).

### Chromosome Number Determination

Chromosome numbers were determined from metaphase cells of actively growing root tips obtained from field transplanted plants (*n* = 22; Supplementary Table 1). Root tips were pre-treated in a 0.002-M aqueous solution of 8-hydroxyquinoline for approximately 24 h at 4°C, fixed in 96% ethanol and 98% acetic acid (3:1) for 1 h; washed in distilled water; hydrolyzed in a mixture of 35% HCl and 96% ethanol (1:1) for 3 min, and finally washed in distilled water again. Permanent squashes were made using the cellophane square method (Murín, 1960), stained with a 7% Giemsa solution in Sörensen phosphate buffer (Fluka Analytical, Munich, Germany), washed, dried, mounted, and observed with a high-power oil immersion objective (Mártonfi et al., 1999). At least two intact c-metaphase plates per plant were observed.

### Relative DNA Content (RGS) Estimation

DNA-ploidy diversity and relative genome size (2C value given in arbitrary units, a.u. hereafter) was estimated for 1002 individuals from 103 populations (Supplementary Tables 1, 2). The 4′,6-diamidino-2-phenylindole flow cytometry (FCM)analyses were performed on young, intact leaf tissues conserved in silica gel. Before analyses, reference plants with counted chromosome numbers (2*n* = 2*x* = 26 and 2*n* = 4*x* = 52, see Supplementary Table 1) were analyzed simultaneously with the most appropriate internal DNA reference standards, and the ratio of their G0/G1 peak positions was recorded. Cytotypes 2*x* and 4*x* were analyzed using *Bellis perennis* L. (2C DNA = 3.38 pg; Schönswetter et al., 2007) as a standard. However, histogram peaks of several accessions termed “peculiar 4x,” Supplementary Table 2 and Supplementary Figures 2, 6) overlapped with that of *B. perennis*, so were analyzed with the alternative standard *Glycine max* “Polanka” (2C DNA = 2.50 pg; Doležel et al., 1994). DNA ploidy levels of analyzed plants with unknown chromosome number were assessed by their peak position relative to the DNA reference standard peak. Details of the laboratory protocol are given in Hodálová et al. (2010). FCM analyses were performed using a Partec CyFlow ML flow cytometer (Partec GmbH, Munster, Germany), equipped with an HBO 100 W mercury arc lamp. The resulting histograms based on 5000 stained nuclei were evaluated using Partec FloMax software (v. 2.52; Partec GmbH, Munster, Germany). The reliability of each measurement was assessed by calculating the coefficients of variation (CV) of both standard and *Stellaria* samples (Supplementary Table 2). Individual analyses with CVs of the G0/G1 peak exceeding the 5% threshold were discarded and re-analyzed. The final quality of RGS measurements, i.e., CVs of standard and *Stellaria* samples ranged between 1.20–4.47 and 0.90–4.47, respectively (Supplementary Table 2). In tetraploid samples with variation in their RGS, simultaneous analyses were performed (Supplementary Figure 2).

### Morphological Differentiation of Sex-Associated Morphs and Cytotypes

Morphological differences between sexes and cytotypes were analyzed using eight quantitative floral traits scored in 995 individuals from 103 populations (Supplementary Tables 1, 3).

Special attention was paid to character selection because phenotypic plasticity has been recurrently evidenced in several *Stellaria* species (e.g., Gadella, 1977; Macdonald et al., 1988; Kurtto, 2001; Kurepin et al., 2015). Inclusion of traits affected by phenotypic plasticity might lead to distorted results and misinterpretations (cf. Slovák et al., 2012), therefore we only measured the following floral traits that were not thought to show strong phenotypic plasticity: the maximum length of the petal, the maximum width of the petal, the depth of the petal incision, the maximum length of the style, the maximum length of the sepal, the maximum width of the sepal, the maximum width of the membranous sepal margin and the length from the base to the widest part of the sepal. Traits associated with anthers were not considered because they were practically absent in male-sterile individuals and to evaluate morphological differences between these two types was meaningless (Figures 2A–D).

We dissected fresh flowers, attached the organs to paper with translucent adhesive tape to preserve original character parameters, and scanned them using a Microtek ScanMaker 9800XL scanner. Scanned material was measured using an Olympus SZ61 stereomicroscope and the QuickPHOTO Micro 2.3 software. The remaining plant parts were dried and deposited as voucher specimens in the herbarium of the Plant Sciences and Biodiversity Center (SAV, Holmgren et al., 1990).

### Data Analysis

#### Morphological Variation Within and Between Sex-Associated Phenotypes and Cytotypes

Redundancy analysis (RDA) (Rao, 1964) based on 995 individuals was used to evaluate interacting effects of cytotype and sex on flower morphology of *S. graminea*. In contrast to univariate techniques, RDA allows the analysis of multivariate morphological data and the testing of the statistical significance of individual terms in a factorial design (Legendre and Anderson, 1999). We conducted the analysis on z-standardized morphological features to eliminate dimensional heterogeneity in the floral traits. A series of randomization tests (1,000 permutations) was used to assess overall significance of the RDA model, and significance of the individual terms and ordination axes, respectively. Since multiple individuals were collected from the same populations, the effect of population identity was partialed-out as a covariate in the RDA. Randomization scheme of the tests was restricted to permutations of observations within populations (spatial blocks) in order to account for spatial autocorrelation inherent in the hierarchical structure of the data (Anderson and Braak, 2003). Variance in the floral traits explained by the model was calculated as a goodness-of-fit measure. To gain more insight into cytotype- and sex-related effects on flower morphology, the RDA was supplemented by generalized linear mixed-effect models on individual floral traits of 995 individuals, using the same parameters as below.

#### Ploidy Level, RGS Variation and Their Association With Sex-Associated Phenotypes

We tested whether the monoploid RGS of an individual (*n* = 1002) differs between cytotypes or plant sexes. We explored the strength of the evidence supporting the hypothesis using generalized linear mixed-effect models (GLMM) (Bolker et al., 2009). As the response is a strictly positive and continuous variable, we used GLMM with Gaussian error distribution and log-link function. The model was formulated to quantify monoploid relative DNA content as a function of cytotype, sex, and their interaction. Population identity was included as a random effect to deal with non-independence in the hierarchical design. The model parameters were tested using likelihood ratio tests (Pinheiro and Bates, 2000). Marginal and conditional determination coefficients were calculated to quantify the proportion of the total variance explained by the fixed effects and by both fixed and random effects, respectively (Nakagawa et al., 2017). We carefully checked residuals for heterogeneity and spatial autocorrelation using diagnostic plots and correlograms (Bjornstad and Falck, 2001). No severe miss-specification of the model was observed.

#### Ecological Drivers of Cytotype and Sex-Associated Phenotype Patterns

Additional models were formulated to estimate the effects of habitat quality on the frequency of occurrence of cytotypes and sexes in populations (*n* = 103). Generalized linear models (GLM) with a binomial distribution and logit link function (McCullagh and Nelder, 1989) were employed to assess the influence of environment on the relative proportion of tetraploids in the populations. Environmental conditions were defined in terms of topographic, climatic and radiation characteristics (Supplementary Table 4). Since the ecological data somehow share the same information on habitat quality, we screened the habitat characteristics for pairwise correlations to prevent multicollinearity problems. Subsequently, altitude and global annual radiation were excluded from the dataset due to a high degree of redundancy concerning mean annual temperature (Pearson’s *r* = −0.92, *p* < 0.0001) and annual photosynthetically active radiation (*r* = 0.88, *p* < 0.0001), respectively. In a subset of four remaining parameters used to formulate GLMs, variance inflation factors (VIFs) did not indicate any clear multicollinearity patterns (all VIFs < 2.1) (Quinn and Keough, 2002). Since the residual variance of the model was more extensive than expected under the binomial distribution, standard errors of the model terms were adjusted for overdispersion (McCullagh and Nelder, 1989). A series of likelihood ratio tests were used to assess the significance of the model coefficients, and non-significant terms were excluded by backward stepwise elimination. Again, diagnostic plots of residuals were used to determine model performance, and we did not find any violation of distributional and independence assumptions in the final model. Variance-function-based R2 was used as a goodness-of-fit measure due to its direct applicability to quasi-models (Zhang, 2017).

Finally, the relationships between environmental conditions and the sex ratio were assessed by GLMM with binomial errors and logit link function. The same subset of four environmental characteristics as above was used to define the habitat. We also included interactions of the four environmental variables with cytotype into the GLMM to further explore potential cytotype-specific effects of environmental drivers. The final model was built as outlined above, but we added an observation-level random effect to account for overdispersion in the proportion data, which provides more accurate estimates of standard errors (Harrison, 2014). All analyses were performed in R (R Core Team, 2019) using the packages AFEX (Singmann et al., 2019), effects (Fox and Weisberg, 2018), lme4 (Bates et al., 2015), ncf (Bjornstad, 2019), and rsq (Zhang, 2020).

## Results

### True Gynodioecy Is Associated With Female-Specific Flower Characters in *Stellaria graminea*

We identified two sexual morphs in *S. graminea* both in diploid and tetraploid cytotypes. The first morphotype corresponds to plants (*n* = 712) bearing hermaphrodite flowers with fully developed organs of both sexes, including well-developed anthers producing viable pollen grains (Figures 2A,B). The median value of pollen viability in hermaphrodites (*n* = 621) was 81.7% (ranging between 28 and 98%; Supplementary Figure 1). We found 20 individuals (2.0%) with flowers having both well-developed and vestigial anthers. So-called gynomonoecious individuals were found in both diploid and tetraploid cytotypes. Since the well-developed anthers produce fertile pollen (pollen viability ranges between 58 and 94%), these individuals were assigned to the hermaphrodite morph. The second sexual morph was represented by male sterile (female) plants (*n* = 290) with flowers having solely vestigial anthers containing no pollen grains (Figures 2C,D).

**FIGURE 3:**
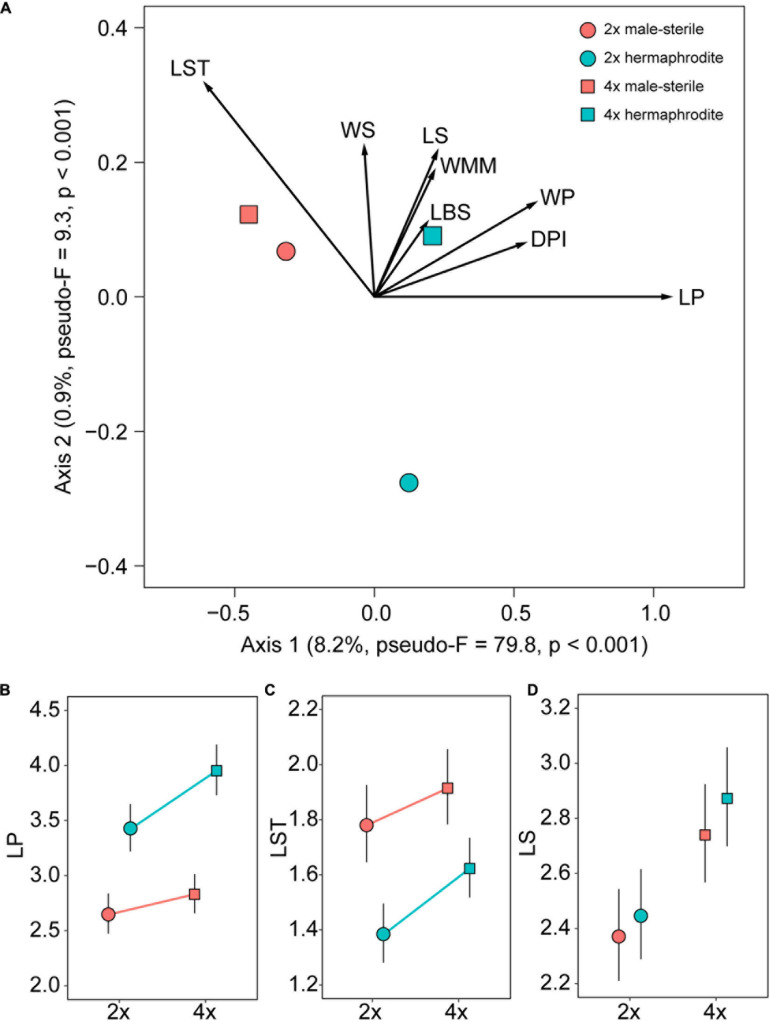
Hermaphrodite and female flowers of *Stellaria graminea* (*n* = 705 and *n* = 289, respectively) display different characters. **(A)** RDA ordination plot showing interacting effects of sex and cytotype on measured floral traits (vectors). Variance explained by the ordination axes and details of significance tests are given in parentheses. Scaling of the ordination plot is focused on correlations between morphological traits. **(B–D)** The effect of cytotype, sex and their interaction on selected measured floral morphological traits. **(B)** The maximum length of the petal (mm). **(C)** The maximum length of the style (mm). **(D)** The maximum length of the sepal (mm). GLMM-based estimates (circles and squares) and their 95% confidence intervals (error bars) are displayed. Significant interactions of sex with cytotype are depicted by lines linking cytotypes within sexes. For the model details see Supplementary Table 1.

The overall flower morphology was significantly affected by both plant cytotype and sex, as indicated by the partial RDA (pseudo-*F* = 30.02, *p* < 0.001, var. explained = 9.2%; Figure 3A). Females and hermaphrodites of both cytotypes were clearly distinguishable from each other and separated in ordination space along the first RDA axis accounting for 8.2% of the variance in morphological data. Hermaphrodites had longer and wider petals, deeper petal incision, and shorter styles compared to females (Figures 3A–D and Supplementary Figures 3A,B). The phenotypic distinction between diploids and tetraploids was less obvious. The cytotypes were separated along the second ordination axis (0.9% variance explained) with hermaphrodites of the two cytotypes showing significantly different flower shapes (pseudo-*F* = 7.68, *p* < 0.001), but flowers of 2*x* and 4*x* females being statistically comparable (pseudo-*F* = 2.78, *p* = 0.054) (Figures 3A–D). The effect of cytotype differed between the sexes (cytotype × sex interaction: pseudo-*F* = 4.05, *p* = 0.002). However, for a subset of traits not obviously involved in reproductive functions, such as sepals, the influence of cytotype was more pronounced than sex, with tetraploids showing larger organs than diploids (Figure 3D and Supplementary Figures 3C,E).

We found well-developed seeds in 759 individuals (Supplementary Table 2 and Supplementary Figure 4), comprising hermaphrodites (2*x*: *n* = 205; 4*x*: *n* = 351) as well as females (2*x*: *n* = 107; 4*x*: *n* = 96).

Of the 103 populations, 90 were sex-dimorphic, harboring hermaphrodite and female individuals, while only 13 contained solely hermaphrodites (Figure 1A). The 20 gynomonoecious individuals were found in 18 different populations (1–2 individuals per population, Supplementary Table 2) which were all sex-dimorphic (Supplementary Table 2).

### Gynodioecy Is Not Specifically Associated With Polyploidy in *S. graminea*

The direct chromosome counting (*n* = 22) revealed the presence of two cytotypes, diploid (2*n* = 2*x* = 26) and tetraploid (2*n* = 4*x* = 52) (Supplementary Table 1 and Supplementary Figure 5). Plants having different RGS, with their histogram peaks overlapping those of the standard *B. perennis* were tetraploids with 2*n* = 4*x* = 52 (Supplementary Table 1 and Supplementary Figure 5).

The RGS-based DNA ploidy level estimation confirmed the presence of DNA-diploids (*n* = 375) and DNA-tetraploids (*n* = 626) (Figure 1B, Supplementary Figure 6, and Supplementary Table 2). A single plant had an RGS value consistent with triploidy (SK-ST32-1; Supplementary Table 2 and Supplementary Figure 6). The real ploidy and number of chromosomes of this individual is uncertain because unfortunately, we could not confirm them by direct chromosome counting.

In sixty-nine (67%) populations, we found only a single cytotype (2*x* – 20 pops; 4*x* – 49 pops.), while 34 (33%) were heteroploid. Diploids were found mainly in the south and south eastern populations (Figure 1B).

When associating cytotypes and sexual morphs, both cytotypes were found in hermaphrodite (2*x*: *n* = 245; 4*x*: *n* = 467) as well as in female morphs (2*x*: *n* = 130; 4*x*: *n* = 159). The single triploid individual seemed to be hermaphrodite (Supplementary Table 2). The two major cytotypes did not differ in pollen viability, since diploids (*n* = 204) had an average pollen viability of 81.5% (ranging between 40 and 99%), while tetraploids (*n* = 417) had 82% (28–98% range) (Supplementary Figure 1 and Supplementary Table 2). Well-developed seeds were produced by plants of both major cytotypes (Supplementary Table 2).

### Females Have Consistently Higher Monoploid Genome Size Compared to Hermaphrodite Individuals

**FIGURE 4:**
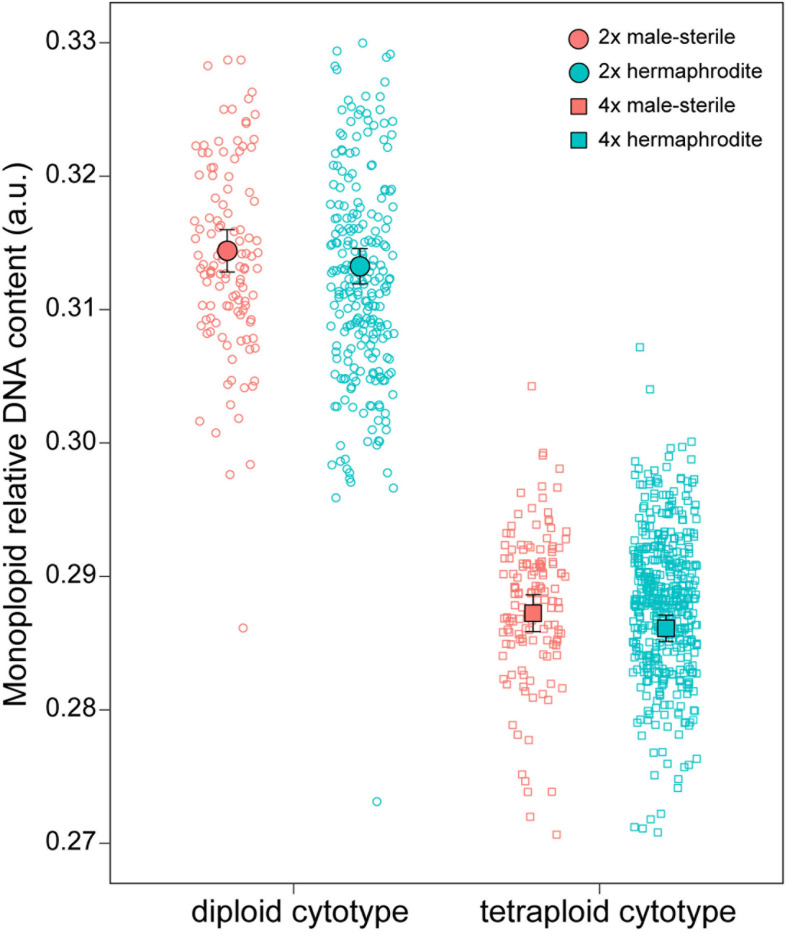
Females (red; *n* = 290) show higher monoploid relative DNA content compared to hermaphrodites (cyan; *n* = 712) in *Stellaria graminea*. Mean differences in monoploid AGS within diploid (*n* = 375) and tetraploid (*n* = 626) cytotypes. Black circles represent mean values, while error bars show 95% confidence intervals.

The RGS ranged from 0.53 a.u (RO1_5) in DNA-diploids to 1.2 a.u. (SK17_7) in DNA-tetraploids, exhibiting 2.28-fold variation (Supplementary Table 2 and Supplementary Figure 6). Mean values of RGS at the monoploid level were 0.313 (range: 0.28 to 0.37 a.u.) in DNA-diploids and 0.29 (range: 0.26 to 0.38 a.u.) in DNA-tetraploids (Figure 4, Supplementary Table 2, and Supplementary Figure 6). The assumed DNA-triploid individual possessed an RGS of 0.79 a.u. (0.26 a.u. in the monoploid genome).

DNA content differed between the sexes, with females showing a significantly higher RGS [χ^2^_(__1__)_ = 5.27, *p* = 0.0217], and between cytotypes, with diploids showing higher monoploid RGS [χ^2^_(__1__)_ = 710.53, *p* < 0.0001; Figure 4]. We found no evidence for an interaction [χ^2^_(__1__)_ = 0.01, *p* = 0.9417]. Explanatory power of the model was very high (*R*^2^_m_ = 0.91, *R*^2^_c_ = 0.98).

### Ploidy Rather Than Sex Shows a Strong Association With Environment

**FIGURE 5:**
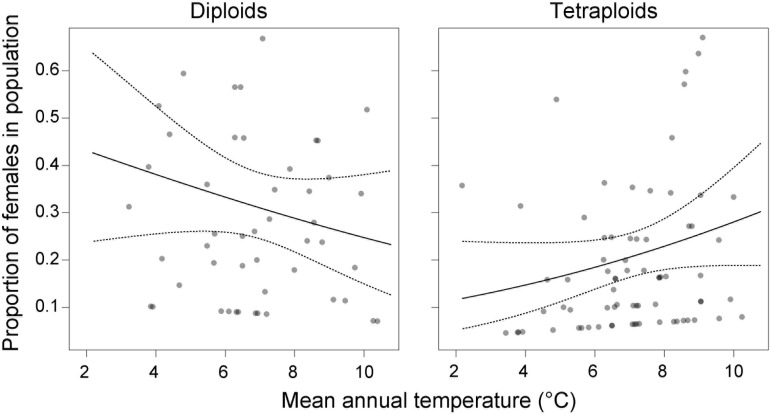
Contrasting effect of mean annual temperature on the proportion of male-sterile individuals in diploid and tetraploid populations of *Stellaria graminea* in the Carpathians. GLMM-predicted proportions (solid lines) and their 95% confidence intervals (dashed lines) are displayed along with partial residuals (a single dot per population of each cytotype).

Among environmental variables (Supplementary Table 1), population sex ratio covaried significantly only with mean annual temperature [χ^2^_(__1__)_ = 11.41, *p* = 0.0097], but in different directions for the two cytotypes [temperature × cytotype: χ^2^_(__1__)_ = 4.45, *p* = 0.0350]. We found more male-sterile individuals at colder sites in diploids but at warmer sites in tetraploids (Figure 5), though this model accounted for only little variation (*R*^2^_m_ = 0.03, *R*^2^_c_ = 0.19).

**FIGURE 6:**
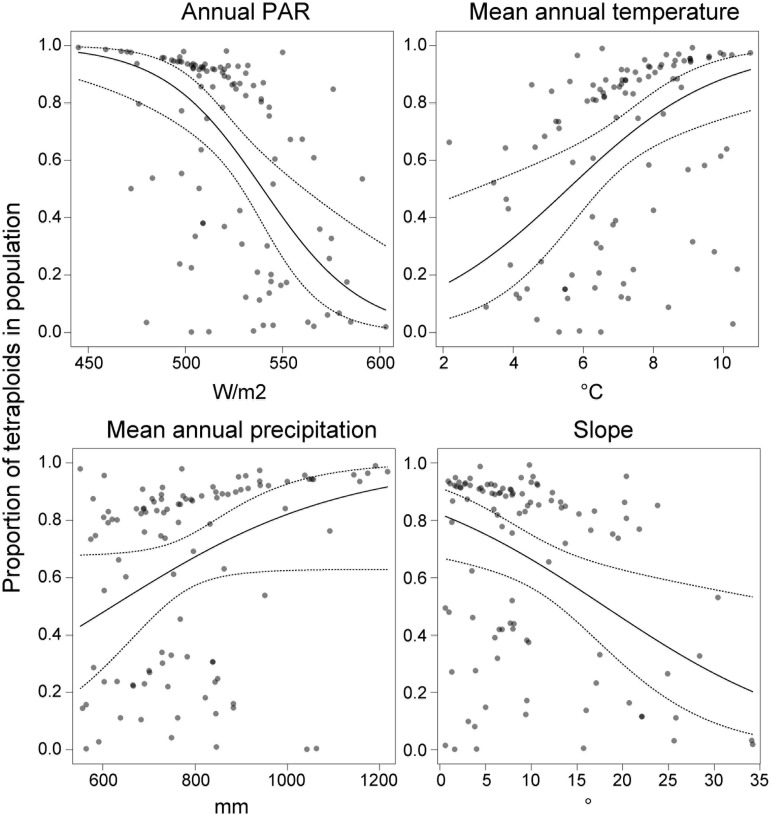
The effect of annual photosynthetically active radiation (PAR), mean annual temperature, precipitation and terrain slope on the proportion of tetraploids (*n* = 626) in the Carpathian populations (*n* = 103) of *Stellaria graminea*. GLM-predicted proportions (solid lines) and their 95% confidence intervals (dashed lines) are displayed along with partial residuals (dots), keeping other variables constant.

Cytotype frequency covaried with Photosynthetically Active Radiation [PAR; *F*_(__1_,_98__)_ = 16.84, *p* < 0.0001], mean annual temperature [*F*_(__1_,_98__)_ = 11.84, *p* = 0.0008], precipitation [*F*_(__1_,_98__)_ = 3.95, *p* = 0.0497] and terrain slope [*F*_(__1_,_98__)_ = 7.45, *p* = 0.0075] (Figure 6). The proportion of tetraploids increased with increasing temperature and precipitation, but decreased with increasing PAR and slope. Overall, the model was significant [*F*_(__4_,_98__)_ = 10.69, *p* < 0.0001] and explained 34% of the total variation.

## Discussion

### True Gynodioecy Is Found in *Stellaria graminea* at Both Diploid and Tetraploid Levels

The vast majority of populations of *S. graminea* from the Carpathians were sexually dimorphic, harboring hermaphrodite and male-sterile individuals. Plants of *S. graminea* bearing male-sterile flowers produced well-developed seeds, indicating no noticeable disruption in female functionality and thus confirming the genuine presence of gynodioecy in this species, as has been demonstrated for *S*. *longipes* Goldie (Philipp, 1980; Dang and Chinnappa, 2007) and suggested for several other *Stellaria* species (e.g., Kurtto, 2001; Chinnappa et al., 2005; Morton, 2005; Slovák et al., 2012). By contrast, while triploid cytotypes in *S. graminea* have been reported (cf. Gadella, 1977; Harmaja, 1992; Kurtto, 2001; Morton, 2005; Goldblatt and Lowry, 2011; Rice et al., 2015), the one triploid individual found in our study suggests that triploid hybrids are rare in nature. Thus, the widespread male-sterile phenotype we observed in our study cannot be explained by the sterility of heteroploid hybrids, and instead is a case of true gynodioecy. This is the first observation of this kind in *S. graminea*.

One of our crucial findings is the presence of gynodioecy in *S. graminea* at both diploid and tetraploid levels. To date, the gynodioecy in *Stellaria* has been proven or suggested almost exclusively for polyploid species complexes such as *S. longipes*, *S. palustris*, and *S. graminea* (Kurtto, 2001; Chinnappa et al., 2005; Dang and Chinnappa, 2007). As gynodioecy has been proposed to be ancestral in Caryophyllaceae (Desfeux et al., 1996), it is likely that gynodioecy in the polyploid *S. graminea* was inherited from diploids, and then sexes spread to reach similar frequencies in polyploid populations. This scenario may nevertheless be hard to clarify depending on the origin of polyploids in *S. graminea* (single vs. multiple origins, auto- vs. allopolyploid), which is currently unknown. In particular, it remains unclear how gynodioecy was inherited from diploids, i.e., either being present in the first polyploid individuals or introgressed by recurrent interploidy or even interspecific gene flow in the case of an allopolyploid origin. The virtual absence of triploids in our study is not in favor of this hypothesis, but interploidy gene flow may still happen *via* unreduced gamete formation (Chinnappa, 1985; Harmaja, 1992).

Several plants of S. *graminea* from the Carpathians were gynomonoecious, i.e., bearing flowers with both sterile and fertile anthers. Such an intermediate stage of sexual expression indicates lability in the sexual polymorphism itself and it is viewed as a transition step from co-sexuality to male-sterility. Gynomonoecy is considered to be a mechanism stabilizing the establishment of the form of gynodioecy known as the gynodioecious–gynomonoecious sexual system (Dufay et al., 2010). Indeed, this kind of instability in sexual dimorphism expression was already observed in several *Stellaria* species (Horne, 1914; Kurtto, 2001; Dang and Chinnappa, 2007). The molecular mechanism leading to the formation of such intermediate sexual morphs relies on incomplete restoration of male-fertility nuclear loci operating in species with the nuclear-cytoplasmic inheritance of gynodioecy (Desfeux et al., 1996; Bernasconi et al., 2009). A certain level of violation in the functionality of nuclear male fertility restorer genes in *S. graminea* populations might also indicate observed oscillation in pollen viability level in hermaphrodites of both ploidies (Supplementary Figure 1). We found no specific patterns in the distribution of these gynomonoecious individuals neither from ploidy level nor geographic distribution viewpoint. This exciting problem deserves further investigation including detailed within and between population screening of sexuality in the species.

### Gynodioecy Is Accompanied With Female Function Specialization in *S. graminea*

Sexual polymorphism impacts the floral morphology not only concerning sexual organs but also in adjacent floral parts. The nearly universal phenotypic trend in gynodioecious species is that the perianth flower size decreases in females compared to their hermaphrodite counterparts (e.g., Delph et al., 1996; Shykoff et al., 2003; Miller et al., 2016; Kamath et al., 2017). We found that female flowers of *S. graminea* had significantly smaller petals compared to hermaphrodites, independently of the cytotype (see also Horne, 1914). However, diploid and tetraploid female flowers were virtually indistinguishable. This could be explained by gynodioecy being inherited from one cytotype to the other. Alternatively, the phenotypic similarity of females could be a result of convergent evolution between diploids and tetraploids. Indeed, the classical Bateman’s (1948) principle postulates that the reproductive success of males (hermaphrodites in the case of *Stellaria*) is pollinator-limited, while it is not the case for females. Therefore, the evolution of larger petals, promoting pollinator attraction, is usually driven by male selective interests rather than female ones (Paterno et al., 2020). In light of this principle, females would be similarly under relaxed selective pressure for pollinator attraction both in diploids and tetraploids, leading to a convergent flower morphology. Nevertheless, *S. graminea* is assumed to be self-compatible (Kurtto, 2001). It is not clear whether this self-compatibility means that *S. graminea* still mostly outbreeds or instead has significant selfing rates. In case selfing is substantial, the selective pressure to attract pollinators by more conspicuous hermaphrodite flowers may be limited. It is therefore necessary to further investigate the mating strategies of *S. graminea* to understand the divergent evolution of female and hermaphrodite petal size in this species. In addition, style length in *S. graminea* was larger in females compared to hermaphrodites, again irrespectively of the ploidy level. Such an increase in size is not rare in gynodioecious females and can be explained by the compensatory relocation of resources to female functions (e.g., Rodríguez-Riaño and Dafni, 2007; Miller et al., 2016). The longer style in females might increase the flower’s visibility despite the smaller size of its corolla, improving female reproductive success. Alternatively, in a context of sexual selection, a longer style may increase the opportunity for mate (pollen tube) selection, which is predicted to be beneficial for the female fitness (Mulcahy, 1979; Stephenson et al., 2003).

Alternatively, female flowers in *S. graminea* may bear longer styles than hermaphrodite flowers because the female styles are more mature when the flowers open, while hermaphrodite flowers are protandrous and their styles gradually elongate during flowering, which might cause at least some of them to be shorter than those of females. Nevertheless, in such a scenario, we would expect that among the hundreds of collected hermaphrodite flowers, some would be at the same maturity stage as female flowers, and thus would have a similar style length. This was not the case, as there was no overlap in style length between female and hermaphrodite flowers (see Figure 3C).

On the other hand, calyx-associated traits, which play a minute or no role in pollinator attraction, showed significant size shifts dependent mostly on the ploidy level. This argues in favor of sexual selection being responsible for the flower divergence between females and hermaphrodites rather than life history events such as polyploidization. Overall, our results suggest that the presence of gynodioecy in *S. graminea* is accompanied with the specialization of female flowers.

### The Association Between Temperature and Female Occurrence Is Likely Due to the Interaction Between Mating Strategy and Other Evolutionary Processes in *S. graminea*

Abiotic ecological factors might directly trigger the formation of sex-associated spatial pattern in gynodioecious plants, for example if female and hermaphrodite individuals of a given species have different habitat optima. Specific environments can act as stressors and can increase female frequencies in populations via increasing the magnitude of reproductive compensation or costs (e.g., Caruso et al., 2016; Rivkin et al., 2016). The majority of studied populations of *S. graminea* in the Carpathians were dimorphic, nevertheless, our analyses showed a mild effect of temperature on the occurrence of females, in a ploidy-dependent manner. In particular, tetraploid females showed a trend to occupy habitats with a warmer climate, which was not the case for diploids. A warmer environment has been associated with an increase in female rate in several gynodioecious plant systems (Alonso and Herrera, 2001; Vaughton and Ramsey, 2005; Caruso and Case, 2007; Ruffatto et al., 2015; Abdusalam et al., 2017). Interestingly, the positive correlation between warm environment and higher frequencies of females in a wild population of *Lobelia syphilitica* L. (Caruso and Case, 2007) was not confirmed in *ex situ* induced temperature regime experiments (Bailey et al., 2017). This suggests that the increased frequency of females with higher temperature is not a direct physiological response. The fact that the temperature is not correlated with the occurrence of females in diploids, but only in tetraploid *S. graminea*, also argues against a direct physiological response, which would most likely be similar between diploids and tetraploids. Instead, the occurrence of females in tetraploids might be related to other evolutionary or demographic processes such as migration or adaptation affecting this cytotype. For example, it remains to be tested whether the propensity to clonality depends on the cytotype, but if it does, it is likely to contribute to the patterns we observe here. Also, our study uncovered that the proportion of tetraploids increased with temperature and precipitation, while it decreased with PAR and slope, and this cytotype-environment association was in general stronger than the one between sex and environment. The occurrence of tetraploid females of *S. graminea* in warmer conditions might thus be related to niche shifts experienced by the tetraploid cytotype and its adaptation to warmer and more humid conditions compared to their diploid counterparts. Besides, an ecological adaptation of tetraploids is plausibly accompanied by another exciting phenomenon, sex-dependent plasticity. This concept implies that the overall vigor of a hermaphrodite might oscillate depending on changes in environmental variables, while a female’s fitness is much more conservative and stay essentially stable irrespectively of habitat conditions (Delph, 1990). This sex dependent advantage enables females to successfully colonize novel, often challenging habitats without additional costs. Thus, although the tetraploid cytotype of *S. graminea* seems to be preadapted to warmer niches, hermaphrodites might be, due to their plasticity, less vigorous in such novel and likely suboptimal conditions. In contrast, this may not be the case for tetraploid females, which may be less plastic and favored in a suboptimal environment.

The cytotype pattern in *S. graminea* from the Carpathians uncovered diffuse mosaic-like structure with frequent cytotype contact zones, which is not commonly seen in similar heteroploid species complexes (but see, e.g., Sonnleitner et al., 2010; Čertner et al., 2019). Nonetheless, when considering the entire sampled area, we can see an essentially clear trend with the prevalence of diploids in Southern and South-eastern Carpathians. At the same time, tetraploids predominate northward in Western Carpathians. Such ploidy dependent south-north differentiation with higher ploidies occupying northern regions and *vice versa* is not rare in the European continent and most plausibly mirrors the Pleistocene history of given species and cytotypes (Ehrendorfer, 1980; Weiss-Schneeweiss et al., 2013; Kolář et al., 2016).

### A Proto-Sex Chromosome Responsible for Gynodioecy in *S. graminea*?

Gynodioecy is considered as an evolutionary transition to dioecy, and this transition might be facilitated if gynodioecy and dioecy share a similar genetic basis, i.e., the presence of sex chromosome(s) building on a nucleo-cytoplasmic determination of male-sterility (CMS). While this has been shown in *Papaya* species (Yu et al., 2008), it remains largely unexplored in other systems. Here, FCM analyses uncovered a significant increase in average values of monoploid relative DNA content in both diploid and tetraploid females of *S. graminea* compared to hermaphrodites. One of the possible reasons for monoploid DNA content increase in females might be the occurrence of homo- or heteromorphic sex chromosomes (Charlesworth, 2002). Indeed, transposable element accumulation is well known to occur in sex chromosomes (Chalopin et al., 2015), and could explain the detectable difference in genome size between females and hermaphrodites. Although sex chromosomes have not been identified in the genus *Stellaria* to date, we cannot entirely rule out their existence here, or at least the existence of proto-sex chromosomes. Indeed, the presence of a female “syndrome” (thinner and shorter petals, longer style) suggests that a group of alleles involved in female flower morphology are genetically linked (potentially with reduced recombination) to the nuclear determinants of the CMS (Frank, 1989; Schnable and Wise, 1998; Wise and Pring, 2002). Heteromorphic chromosomes were evidenced in related genus *Silene* and were shown to have evolved at least twice independently in this genus (Ming et al., 2007; Slancarova et al., 2013; Vyskot and Hobza, 2015; Bačovský et al., 2020). Finally, in different *Papaya* species, the same sex chromosome determines gynodioecy and dioecy (Yu et al., 2008). Additional genetic analyses, such as a full diallel crossing design, in *S. graminea* are required to test these hypotheses.

Altogether, our study is the first to report gynodioecious females in *S. graminea*, which could have been mistaken for sterile triploid hybrids. To the question “eunuchs or females?”, we can thus answer “females.” It provides a comprehensive set of data to further explore gynodioecy, its morphological, ecological, cytogeographical and genetic causes and consequences. The present results argue against the role of gynodioecy in the establishment of polyploids, as well as the direct role of the environment as a trigger to produce female flowers. Finally, this work suggests that gynodioecy in *S. graminea* might rely on sex chromosome(s), a feature that may facilitate the transition toward dioecy.

## Data Availability Statement

The original contributions presented in the study are included in the article/Supplementary Material, further inquiries can be directed to the corresponding author/s.

## Author Contributions

MSl and JK designed the study. EG, JK, and MSl performed the research. EG, JK, and LM collected the data. JK and MSv analyzed the data. MSl and CL interpreted the data. MSl, MSv, and CL wrote the manuscript. All authors contributed to the article and approved the submitted version.

## Conflict of Interest

The authors declare that the research was conducted in the absence of any commercial or financial relationships that could be construed as a potential conflict of interest.
