# Supplementary material for: Eunuchs or Females? Causes and Consequences of Gynodioecy on Morphology, Ploidy, and Ecology of Stellaria graminea L. (Caryophyllaceae)
Source: Front Plant Sci. 2021 Apr 12;12:589093. doi: 10.3389/fpls.2021.589093 (PMC8072285; doi:10.3389/fpls.2021.589093)
Supplement: Supplementary file 1 [file Data_Sheet_1.docx]

***Supplementary Material***

**Eunuchs or females? Causes and consequences of gynodioecy on morphology, ploidy and ecology of *Stellaria graminea* L. (Caryophylaceae)**

Jaromír Kučera^1,⁑^, Marek Svitok^2,3,⁑^, Eliška Gbúrová Štubňová^1,4^, Lenka Martónfiová^5^, Clément Lafon Placette^6^ and Marek Slovák^1, 6, *^

## The following Supplementary Material is available for this article:


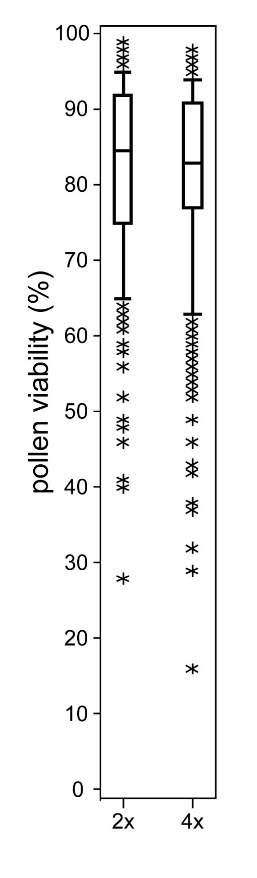


**SUPPLEMENTARY FIGURE S1** Boxplots displaying the variation in pollen fertility between cytotypes of hermaphrodite individuals of S. graminea. Comparison is made between all screen diploid (n = 204) and all tetraploid individuals (n = 417). Rectangles define the 25th and 75th percentiles; horizontal lines show the median; whiskers are from the 10th to the 90th percentiles; asterisks indicate the extreme values.

**
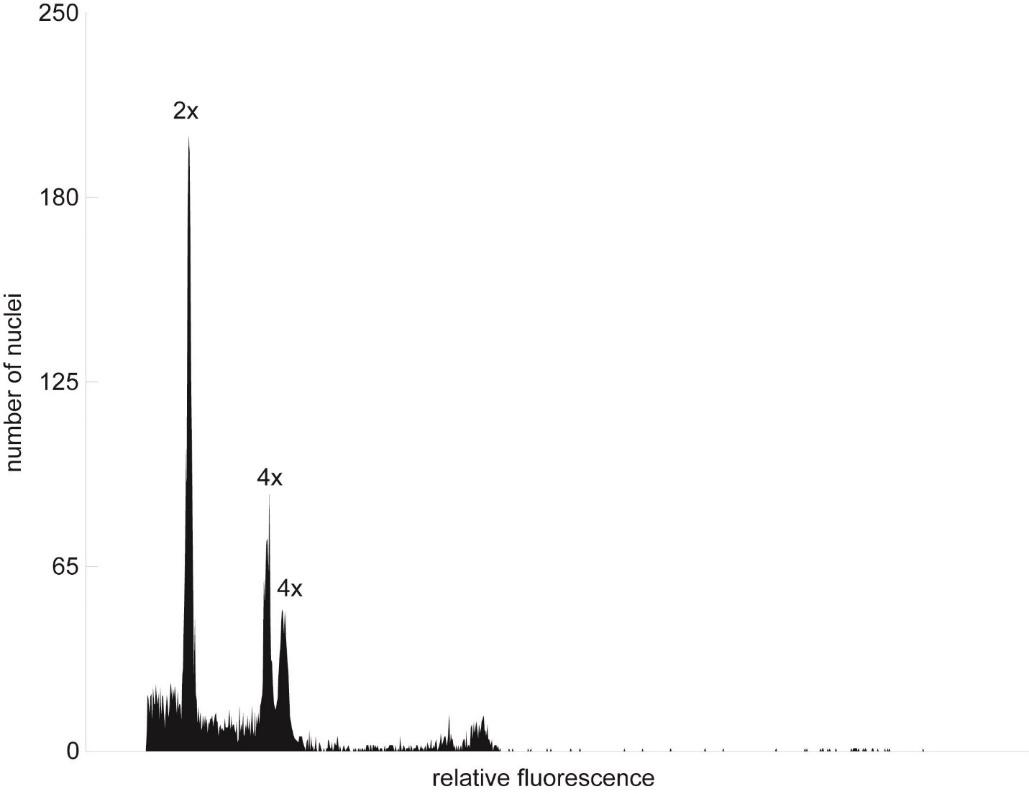
**

**SUPPLEMENTARY FIGURE S2** Flow cytometry histogram showing differences in relative DNA content of diploid and two tetraploid cytotypes detected in *S. graminea.* The analysis was run with a one diploid accession RO15_8, one ‘peculiar tetraploid’ accessions SK3_6 and one tetraploid accession SK24_4. Nuclei of all individuals were isolated, stained with DAPI and analysed simultaneously.


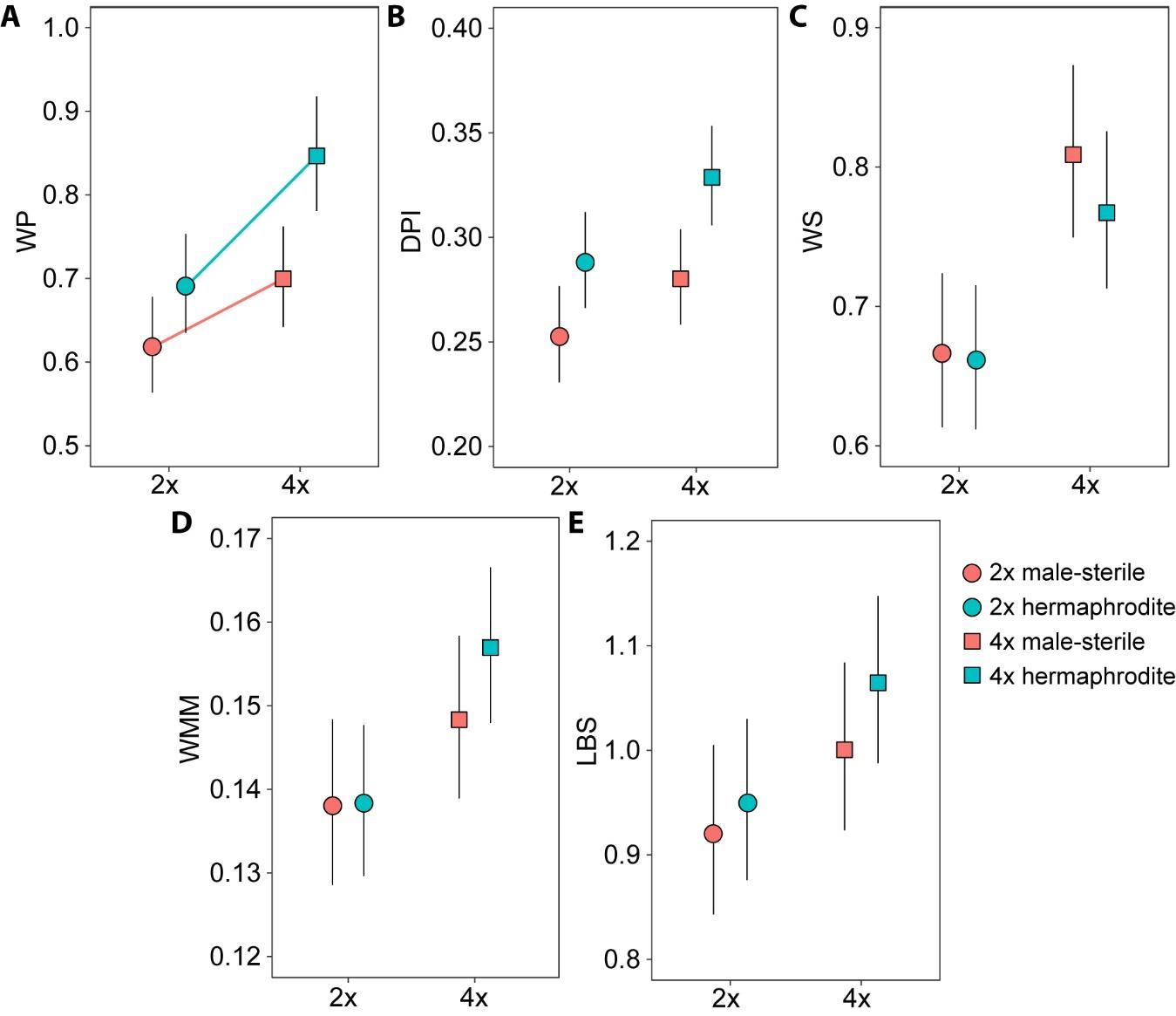


**SUPPLEMENTARY FIGURE S3**. Hermaphrodite (n=705) and female flowers (n=289), display different characters. The effect of cytotype, sex and their interaction on selected measured floral morphological traits. The maximum width of the petal (mm). **(B)** The depth of the petal incision (mm). **(C)** the maximum width of the sepal (mm). **(D)** the maximum width of the membranous sepal margin (mm). **(E** the length from the base to the widest part of sepal (mm). GLMM-based estimates (circles and squares) and their 95% confidence intervals (error bars) are displayed. Significant interactions of cytotype with sex are depicted by lines linking cytotypes within sexes. For the model details see Supplementary Table S1.


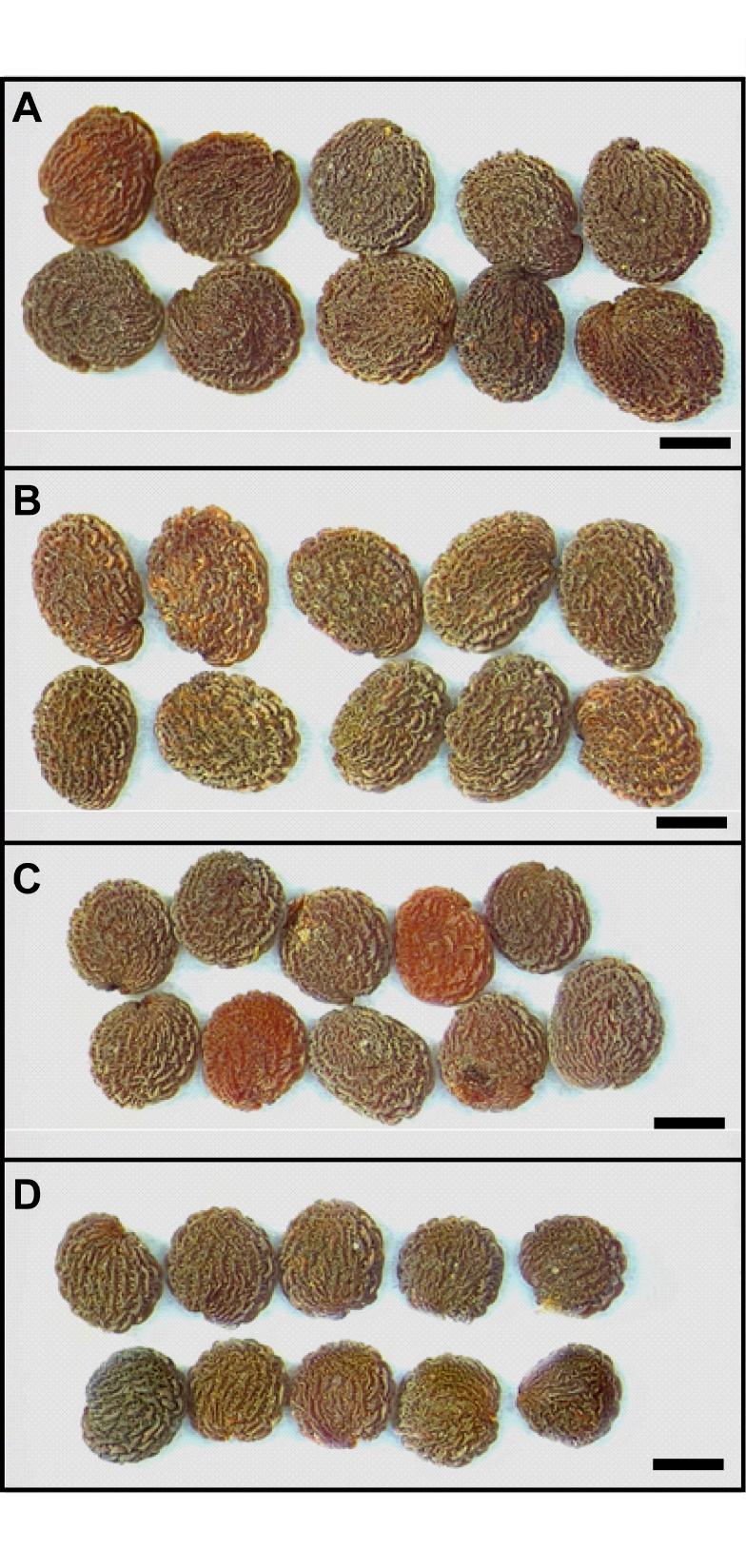


**SUPPLEMENTARY FIGURE S4** Microphotograph of seeds from analysed gender associated morphs and cytotypes of *S. graminea.* **(A)** Hermaphrodite tetraploid morph (locality ST RO38-9). (**B)** Male-sterile tetraploid morp.h (locality ST UA10-7). **(C)** Hermaphrodite diploid morph (locality ST RO37-7). **(D)** Male-sterile diploid morph (locality ST RO23-6). For locality codes and details, see Supplementary Table S1. Scale bare is 1000 µm.


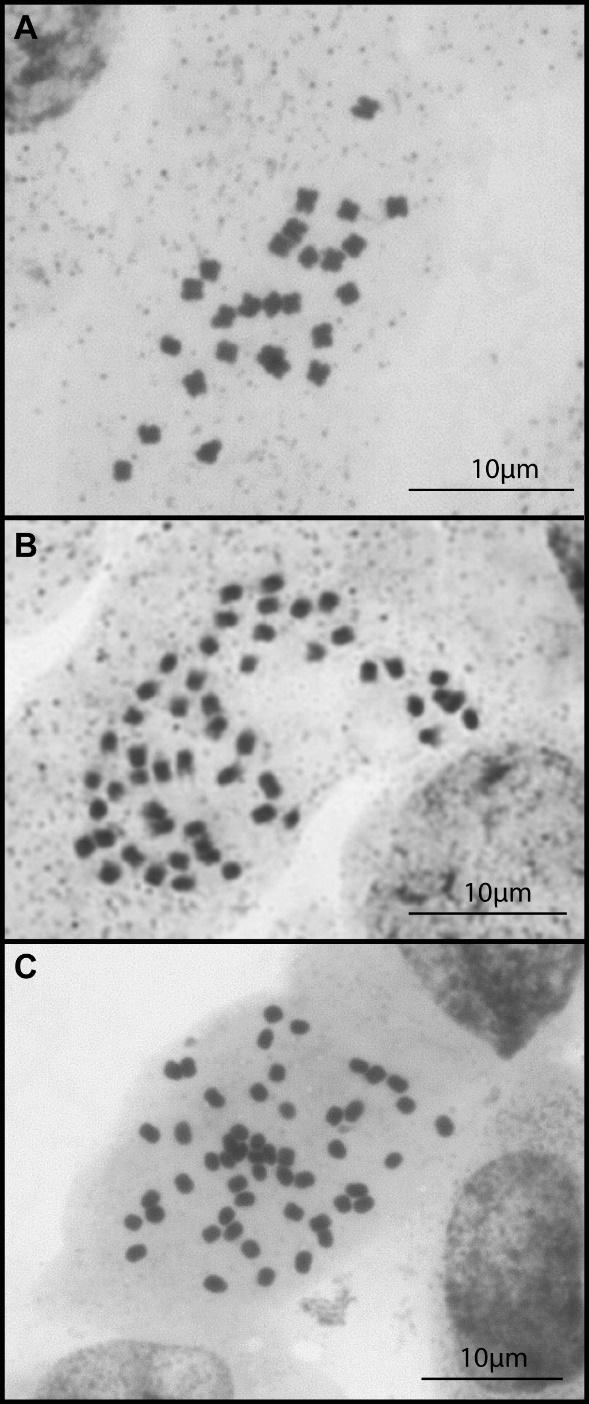


**SUPPLEMENTARY FIGURE S5** Mitotic metaphases of *S. graminea* cytotypes. **(A)** The diploid cytotype with 2*n* = 2*x* = 26 (locality SK ST2). **(B)**The ‘peculiar’ tetraploid cytotype with 2*n* = 4*x* = 52 having different absolute genome size overlapping with that of *Bellis perennis* L. as an internal reference standard (locality ST SK6) **(C)** The tetraploid cytotype with 2*n* = 4*x* = 52 (locality ST SK11).  For locality codes and details, see Supplementary Table S1.

**
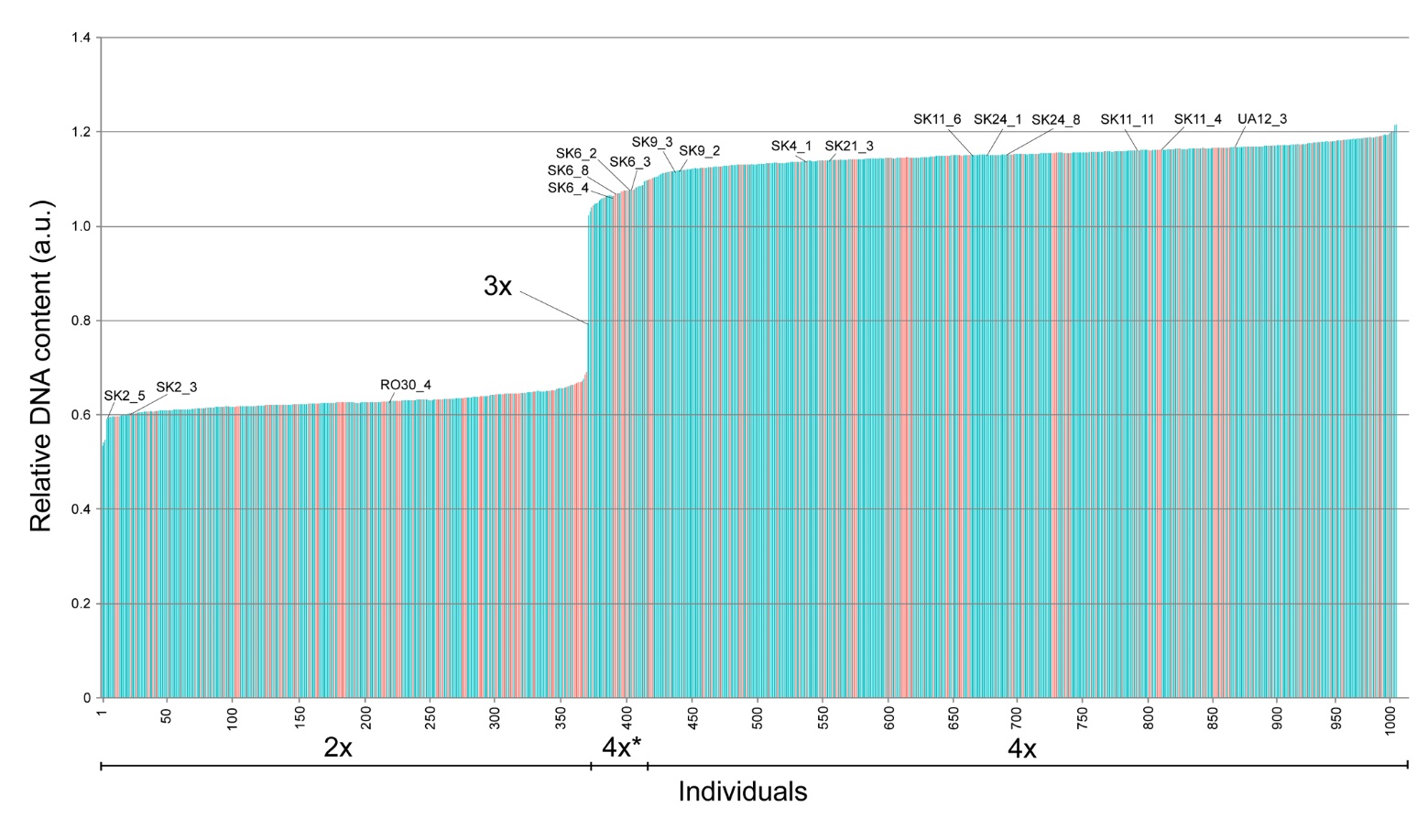
**

**SUPPLEMENTARY FIGURE S6** The histogram of relative DNA content of females (red; n=290) and hermaphrodites (cyan; n=712) in *S. graminea.* The total bar height displays 2C-values. The bars are sorted in ascending C-value order. The reference individuals in which the chromosomes were counted are labelled with population/individual code following Supplementary Table S1. The ploidy levels are indicated below the bars except triploid cytotype marked directly in the diagram.

**SUPPLEMENTARY TABLE S1** The list of sample sites of *S. graminea* used in the study. Locality details are as follows: the country code (SK – Slovakia, RO – Romania and UA – Ukraine); the name of mountain range/lowland and peak or nearest village or city, the habitat type, coordinates; the date of collection, the name of collectors: EŠ – Eliška Štubňová, JK – Jaromír Kučera, MS – Marek Slovák; MA– morphological analyses; RGS - relative genome size analyses. Voucher specimens are deposited in SAV.

| **Locality Code** | **Locality details** | **No. of individuals analyzed per population** | | **Chromosome number and ploidy level** |
| --- | --- | --- | --- | --- |
|  |  | **MA** | **RGS** |  |
| SK-ST1 | Slovakia; Považský Inovec Mts., Javorníček Mt., xerotermous meadow; 48°40´21´´N, 17°57´01´´E; 572 m; 21 Aug. 2010; leg. JK | 6 | 6 | - |
| SK-ST2 | Slovakia; Záhorská nížina lowland, near Závod village, wet meadow; 48°32´05´´N, 17°00´20´´E; 152 m; 8 Jul. 2011; leg. JK & MS | 10 | 10 | 2n=2x=26 (two individuals) |
| SK-ST3 * | Slovakia; Malé Karpaty Mts., Pezinská Baba Mt., mesophylous meadow; 48°20´58´´N, 17°11´23´´E; 519 m; 8 Jul. 2011; leg. JK & MS | 10 | 10 | 2n=4x=52 (two individuals) |
| SK-ST4 | Slovakia; Biele Karpaty Mts., near to the Topolecká village, mesophylous meadow; 48°49´46´´N, 17°38´41´´E; 439 m; 9 Jul. 2011; leg. JK | 10 | 10 | 2n=4x=52 (one individual) |
| SK-ST5 | Slovakia; Podunajská nížina lowland, near Budmerice village, meadow in castle park; 48°21´47´´N, 17°23´42´´E; 208 m; 10 Jul. 2011; leg. JK | 10 | 10 | - |
| SK-ST6 | Slovakia; Záhorská nížina lowland, Devínska NováVes - the part of city Bratislava, wet meadow; 48°12´48´´N, 16°58´05´´E; 138 m; 11 Jul. 2011; leg. JK & MS | 10 | 10 | 2n=4x=52 (six individuals) |
| SK-ST7 | Slovakia; Malé Karpaty Mts., near Borinka village, mesophylous meadow; 48°15´08´´N, 17°06´44´´E; 293 m; 11 Jul.2011; leg. JK & MS | 10 | 10 | - |
| SK-ST8 | Slovakia; Malé Karpaty Mts., near Plavecké Podhradie village, meadow; 48°29´15´´N, 17°15´45´´E; 234 m; 11 Jul. 2011; leg. JK & MS | 10 | 10 | - |
| SK-ST9 | Slovakia; Záhorská nížina lowland, Mešterova lúka protected area, wet meadow; 48°28´36´´N, 17°03´31´´E; 177 m; 11 Jul.2011; leg. JK & MS | 9 | 9 | 2n=4x=52 (two individuals) |
| SK-ST10 | Slovakia; Strážovské vrchy Mts., near Peťovka village, mowed meadow; 48°50´57´´N, 18°10´09´´E; 334 m; 2 Aug. 2011; leg. JK & MS | 10 | 10 | - |
| SK-ST11 | Slovakia; Biele Karpaty Mts., near Krivoklát village, meadow; 49°03´01´´N, 18°08´54´´E; 394 m; 2 Aug. 2011; leg. JK & MS | 10 | 10 | 2n=4x=52 (three individuals) |
| SK-ST12 | Slovakia; Strážovské vrchy Mts., near Zliechov village, meadow; 48°56´46´´N, 18°27´15´´E; 724 m; 2 Aug. 2011; leg. JK & MS | 10 | 10 | - |
| SK-ST13 | Slovakia; Bukovské vrchy Mts., Ubľa village, wet meadow; 48°54´59´´N, 22°21´52´´E; 226 m; 18 Aug. 2011; leg. JK & MS | 10 | 10 | - |
| SK-ST14 | Slovakia; Vihorlat Mts., near Zemplínske Hámre village, wet meadow; 48°56´18´´N, 22°09´38´´E; 436 m; 18 Aug. 2011; leg. JK & MS | 10 | 10 | - |
| SK-ST15 | Slovakia; Nízke Beskydy Mts., near Volica village, wet pasture; 49°08´47´´N, 21°55´32´´E; 268 m; 18 Aug. 2011; leg. JK & MS | 10 | 10 | - |
| SK-ST16 | Slovakia; Nízke Beskydy Mts., Nižný Mirošov village, mesophylous meadow; 49°22´13´´N, 21°28´14´´E; 313 m; 18 Aug. 2011; leg. JK & MS | 10 | 10 | - |
| SK-ST17 | Slovakia; Šarišská vrchovina Mts., Lipovce village, mesophylous meadow; 49°03´29´´N, 20°56´32´´E; 614 m; 19 Aug. 2011; leg. JK & MS | 10 | 10 | - |
| SK-ST18 | Slovakia; Spišské vrchy Mts., Levočská dolina valley, mesophylous meadow; 49°03´44´´N, 20°35´32´´E; 594 m; 19 Aug. 2011; leg. JK & MS | 10 | 10 | - |
| SK-ST19 | Slovakia; Slovenský raj Mts., Vernár village, mesophylous meadow; 48°54´57´´N, 20°15´52´´E; 789 m; 19 Aug. 2011; leg. JK & MS | 10 | 10 | - |
| SK-ST20 | Slovakia; Západné Beskydy Mts., VeľkáRača Mt., forest clearings; 49°24´45´´N, 18°57´25´´E; 1130 m; 27 Aug. 2011; leg. JK | 10 | 10 | - |
| SK-ST21 | Slovakia; Západné Beskydy Mts., U Papajov settlement, mesophylous meadow; 49°20´56´´N, 18°25´54´´E; 720 m; 28 Aug. 2011; leg. JK | 10 | 10 | 2n=4x=52 (two individuals) |
| SK-ST22 | Slovakia; Nízke Tatry Mts., near Chata M. R. Štefánika cottage, mountain meadow; 48°55´36´´N, 19°39´02´´E; 1715 m; 19 Sep. 2011; leg. MS | 9 | 9 | - |
| SK-ST23 | Slovakia; Nízke Tatry Mts., Pavčina Lehota village, ruderal meadow; 49°02´15´´N, 19°33´52´´E; 694 m; 17 Sep. 2011; leg. MS | 10 | 10 | - |
| SK-ST24 | Slovakia; Malá Fatra Mts., above Fačkovské sedlosadle, mountain meadow; 48°57´52´´N, 18°37´27´´E; 903 m; 6 Oct. 2011; leg. JK | 9 | 9 | 2n=4x=52 (two individuals) |
| SK-ST25 | Slovakia; Pohronský Inovec Mts., near Hostie village, mesophylous meadow; 48°28´06´´N, 18°27´55´´E; 266 m; 16 Jul. 2012; leg. JK & MS | 7 | 7 | - |
| SK-ST26 | Slovakia; Slovenské rudohorie Mts., near Strelníky village, mountain meadow; 48°43´03´´N, 19°24´23´´E; 703 m; 17 Jul. 2012; leg. JK, MS & EŠ | 10 | 10 | - |
| SK-ST27 | Slovakia; Slovenské rudohorie Mts., near Polomka village, wet meadow; 48°50´59´´N, 19°50´11´´E; 574 m; 17 Jul. 2012; leg. JK, MS & EŠ | 10 | 10 | - |
| SK-ST28 | Slovakia; Muránska planina Mts., Dielik sadle, wet meadow; 48°42´02´´N, 19°59´04´´E; 578 m; 17 Jul. 2012; leg. JK, MS & EŠ | 10 | 10 | - |
| SK-ST29 | Slovakia; Drienčanský kras Mts., near Ratkovská Lehota village, xerotermous meadow; 48°33´56´´N, 20°06´15´´E; 269 m; 17 Jul. 2012; leg. JK, MS & EŠ | 10 | 10 | - |
| SK-ST30 | Slovakia; Slovenské rudohorie Mts., Čučma village, glade; 48°40´35´´N, 20°32´32´´E; 353 m; 18 Jul. 2012; leg. JK, MS & EŠ | 10 | 10 | - |
| SK-ST31 | Slovakia; Slovenský kras Mts., Hačava village, mesophylous meadow; 48°40´08´´N, 20°50´03´´E; 651 m; 18 Jul. 2012; leg. JK, MS & EŠ | 10 | 10 | - |
| SK-ST32 | Slovakia; Slovenské rudohorie Mts., under Kojšova hoľa Mt., glade; 48°46´17´´N, 20°59´10´´E; 982 m; 18 Jul. 2012; leg. JK, MS & EŠ | 8 | 8 | - |
| SK-ST33 | Slovakia; Slanské vrchy Mts., Banské village, wet meadow; 48°48´50´´N, 21°31´36´´E; 653 m; 18 Jul. 2012; leg. JK, MS & EŠ | 10 | 10 | - |
| SK-ST34 | Slovakia; Vihorlat Mts., near Porúbka village, mesophylous meadow; 48°51´04´´N, 21°58´07´´E; 422 m; 18 Jul. 2012; leg. JK, MS & EŠ | 10 | 10 | - |
| SK-ST35 | Slovakia; Nízke Beskydy Mts., near Bardejov city, mesophylous meadow; 49°16´01´´N, 21°17´54´´E; 321 m; 19 Jul. 2012; leg. JK, MS & EŠ | 10 | 10 | - |
| SK-ST36 | Slovakia; Spišské vrchy Mts., near Plaveč castle ruin, meadow; 49°15´16´´N, 20°50´37´´E; 491 m; 19 Jul. 2012; leg. JK, MS & EŠ | 10 | 10 | - |
| SK-ST37 | Slovakia; Pieniny Mts., Haligovce village, ruderal meadow; 49°22´46´´N, 20°26´26´´E; 501 m; 19 Jul. 2012; leg. JK, MS & EŠ | 10 | 10 | - |
| SK-ST38 | Slovakia; Belianske Tatry Mts., Ždiar village, mountain meadow; 49°16´01´´N, 20°15´20´´E; 898 m; 19 Jul. 2012; leg. JK, MS & EŠ | 10 | 10 | - |
| SK-ST39 | Slovakia; Vysoké Tatry Mts., near Štrbské pleso glacial lake, mountain meadow; 49°07´14´´N, 20°04´31´´E; 1225 m; 19 Jul. 2012; leg. JK, MS & EŠ | 10 | 10 | - |
| SK-ST40 | Slovakia; Západné Tatry Mts., near Primula cottage, wet meadow; 49°15´04´´N, 19°42´14´´E; 993 m; 20 Jul. 2012; leg. JK, MS & EŠ | 10 | 10 | - |
| SK-ST41 | Slovakia; Západné Beskydy Mts., near Slaná hora settlement, wet meadow; 49°31´36´´N, 19°28´26´´E; 749 m; 20 Jul. 2012; leg. JK, MS & EŠ | 10 | 10 | - |
| SK-ST42 | Slovakia; Chočské vrchy Mts., near Jasenová village, ruderal meadow; 49°10´14´´N, 19°17´51´´E; 567 m; 20 Jul. 2012; leg. JK, MS & EŠ | 10 | 10 | - |
| SK-ST43 | Slovakia; Malá Fatra Mts., Vrátna dolina valley, wet meadow; 49°13´54´´N, 19°01´59´´E; 639 m; 20 Jul. 2012; leg. JK, MS & EŠ | 10 | 10 | - |
| SK-ST44 | Slovakia; Veľká Fatra Mts., near Malý Šturec sadle, mountain meadow; 48°50´13´´N, 18°57´40´´E; 728 m; 20 Jul. 2012; leg. JK, MS & EŠ | 10 | 10 | - |
| SK-ST45 | Slovakia; Nízke Tatry Mts., Motyčky village, edge of forest; 48°51´26´´N, 19°10´28´´E; 703 m; 4 Aug. 2012; leg. EŠ | 10 | 10 | - |
| SK-ST46 | Slovakia; Západné Tatry Mts., near Žiarska chata cottage, ruderal meadow; 49°10´55´´N, 19°43´14´´E; 1311 m; 7 Aug. 2012; leg. EŠ | 10 | 10 | - |
| SK-ST47 | Slovakia; Slovenský raj Mts., near Podlesok village, edge of forest; 48°57´47´´N, 20°23´18´´E; 560 m; 22 Aug. 2012; leg. EŠ | 10 | 10 | - |
| SK-ST48 | Slovakia; Poľana Mts., near Poľana hotel, mountain meadow; 48°37´37´´N, 19°27´58´´E; 1302 m; 8 Sep. 2012; leg. MS & EŠ | 10 | 10 | - |
| SK-ST49 | Slovakia; Štiavnické vrchy Mts., Štiavnické Bane village, mesophylous meadow; 48°26´03´´N, 18°51´33´´E; 682 m; 8 Sep. 2012; leg. MS & EŠ | 10 | 10 | - |
| SK-ST50 | Slovakia; Kremnické vrchy Mts., Skalka Mt., ruderal meadow; 48°44´24´´N, 18°59´09´´E; 1161 m; 8 Sep. 2012; leg. MS & EŠ | 9 | 9 | - |
| UA-ST1 | Ukraine; Zakarpastkeperedgirya Mts., Ternovo village, ruderal wet meadow; 48°05´29´´N, 23°45´04´´E; 291 m; 16 Aug. 2011; leg. JK & MS | 10 | 10 | - |
| UA-ST2 | Ukraine; Marmaroski Alpi Mts., Dilove village, meadow; 47°57´18´´N, 24°11´16´´E; 378 m; 16 Aug. 2011; leg. JK & MS | 10 | 10 | - |
| UA-ST3 | Ukraine; Čivčino-Grinyavskigori Mts., Jablunickij sadle, mountain meadow; 48°18´23´´N, 24°27´03´´E; 895 m; 16 Aug. 2011; leg. JK & MS | 10 | 10 | - |
| UA-ST4 | Ukraine; Prikarpattya Mts., Sokolivka village, wet pasture; 48°16´16´´N, 24°59´11´´E; 501 m; 16 Aug. 2011; leg. JK & MS | 10 | 10 | - |
| UA-ST5 | Ukraine; Prikarpattya Mts., Nadvirna city, wet meadow; 48°40´01´´N, 24°34´04´´E; 464 m; 17 Aug. 2011; leg. JK & MS | 10 | 10 | - |
| UA-ST6 | Ukraine; Prikarpattya Mts., near Dolina village, wet meadow; 48°57´09´´N, 23°56´00´´E; 445 m; 17 Aug. 2011; leg. JK & MS | 10 | 10 | - |
| UA-ST7 | Ukraine; Gorgani Mts., Viškivskij sadle, pasture; 48°44´31´´N, 23°40´54´´E; 931 m; 17 Aug. 2011; leg. JK & MS | 10 | 10 | - |
| UA-ST8 | Ukraine; Schidni Beskidii Nizki Polonini Mts., near Podobovec village, wet meadow; 48°40´40´´N, 23°16´41´´E; 692 m; 17 Aug. 2011; leg. JK & MS | 10 | 10 | - |
| UA-ST9 | Ukraine; Schidni Beskidii Nizki Polonini Mts., near Bilasovitsa village, meadow; 48°51´22´´N, 23°07´03´´E; 753 m; 17 Aug. 2011; leg. JK & MS | 9 | 9 | - |
| UA-ST10 | Ukraine; Prikarpattya Mts., near Lišnja village, wet meadow; 49°25´17´´N, 23°26´04´´E; 313 m; 17 Aug. 2011; leg. JK & MS | 10 | 10 | - |
| UA-ST11 | Ukraine; Prikarpattya Mts., Strilki village, pasture; 49°19´01´´N, 22°57´42´´E; 410 m; 18 Aug. 2011; leg. JK & MS | 4 | 10 | - |
| UA-ST12 | Ukraine; Schidni Beskidii Nizki Polonini Mts., near Borinja village, mountain meadow; 49°05´46´´N, 22°59´46´´E; 747 m; 18 Aug. 2011; leg. JK & MS | 10 | 10 | 2n=4x=52 (one individual) |
| RO-ST1 | Romania; Munţii Parâng Mts., near Groapa Seâca pass, mountain meadow; 45°24´33´´N, 23°35´03´´E; 1532 m; 4 Aug. 2010, leg. JK & MS | 9 | 9 | - |
| RO-ST2 | Romania; Munţii Retezat Mts., Tomeasa village, ruderal meadow; 45°21´39´´N, 22°44´31´´E; 904 m; 5 Aug. 2010, leg. JK & MS | 8 | 8 | - |
| RO-ST3 | Romania; Munţii Piatra Craiului Mts., near Magura village, meadow; 45°31´51´´N, 25°17´28´´E; 977 m; 6 Aug. 2010, leg. JK & MS | 10 | 10 | 2n=2x=26 (one individual) |
| RO-ST4 | Romania; Munţii Bucegi Mts., Sinaia village, near Cota 1400 mountain hotel, mountain meadow; 45°21´16´´N, 25°30´46´´E; 1509 m; 7 Aug. 2010, leg. JK & MS | 8 | 8 | - |
| RO-ST5 | Romania; Munţii Ciomatu Mts., near BăileTuşnad town, meadow; 46°07´38´´N, 25°51´25´´E; 665 m; 8 Aug. 2010, leg. JK & MS | 8 | 8 | - |
| RO-ST6 | Romania; Munţii Hăşmaş Mts., near Lacu Roşu lake, meadow; 46°46´48´´N, 25°45´03´´E; 1066 m; 9 Aug. 2010, leg. JK & MS | 9 | 9 | - |
| RO-ST7 | Romania; Munţii Bistriței Mts., Poiana Largului Mt., meadow slopes near road; 47°05´28´´N, 25°57´28´´E; 533 m; 9 Aug. 2010, leg. JK & MS | 10 | 10 | - |
| RO-ST8 | Romania; Munţii Rodnei Mts., near Cârlibaba village, meadow; 47°33´41´´N, 25°02´38´´E; 1006 m; 10 Aug. 2010, leg. JK & MS | 10 | 10 | - |
| RO-ST9 | Romania; Munţii Rodnei Mts., Prislop pass, mountain meadow; 47°36´35´´N, 24°51´26´´E; 1410 m; 10 Aug. 2010, leg. JK & MS | 8 | 8 | - |
| RO-ST10 | Romania; Munţii Făgărăş Mts., near Capu Piscului village, ruderal meadow; 44°59´18´´N, 24°44´36´´E; 325 m; 22 Jul. 2011; leg. JK & MS | 9 | 9 | - |
| RO-ST11 | Romania; Munţii Făgărăş Mts., near Cârtişoara village, edge of forest; 45°39´14´´N, 24°36´15´´E; 1376 m; 22 Jul. 2011; leg. JK & MS | 10 | 10 | - |
| RO-ST12 | Romania; Podişul Hârtibaciului Mts., near Bradu village, Poiana Bradului Mt., ruderal meadow; 45°42´53´´N, 24°18´41´´E; 408 m; 23 Jul. 2011; leg. JK & MS | 10 | 10 | - |
| RO-ST13 | Romania; Munţii Şureanu Mts., near Orăştie town, ruderal meadow; 45°51´39´´N, 23°12´55´´E; 217 m; 23 Jul. 2011; leg. JK & MS | 10 | 10 | - |
| RO-ST14 | Romania; Munţii Zarandului Mts., Târnava de Cris village, ruderal meadow; 46°11´03´´N, 22°39´19´´E; 218 m; 23 Jul. 2011; leg. JK & MS | 10 | 10 | - |
| RO-ST15 | Romania; Munţii Pădurea CraiuluiMts., near Pocola village, edge of forest; 46°42´34´´N, 22°15´35´´E; 237 m; 23 Jul. 2011; leg. JK & MS | 10 | 10 | - |
| RO-ST16 | Romania; Munţii Plopiş Mts., near Borod village, ruderal meadow; 46°59´58´´N, 22°36´12´´E; 336 m; 11 Aug. 2011; leg. JK & MS | 10 | 10 | - |
| RO-ST17 | Romania; Muntele Mare Mts., near Someşu Cald lake, meadow; 46°43´53´´N, 23°20´18´´E; 466 m; 12 Aug. 2011; leg. JK & MS | 10 | 10 | - |
| RO-ST18 | Romania; Câmpia Transilvaniei lowland, Suatu city, wet pasture; 46°47´54´´N, 23°57´13´´E; 377 m; 12 Aug. 2011; leg. JK & MS | 10 | 10 | - |
| RO-ST19 | Romania; Câmpia Transilvaniei lowland, Fărăgău village, wet mowed meadow; 46°45´50´´N, 24°32´47´´E; 402 m; 12 Aug. 2011; leg. JK & MS | 10 | 10 | - |
| RO-ST20 | Romania; Munţii Gurghiu Mts., Eremitu village, meadow; 46°39´04´´N, 24°58´34´´E; 563 m; 12 Aug. 2011; leg. JK & MS | 10 | 10 | - |
| RO-ST21 | Romania; Munţii Gurghiu Mts., Pasul Bucinsadle, pastures; 46°39´19´´N, 25°17´48´´E; 1282 m; 12 Aug. 2011; leg. JK & MS | 10 | 10 | - |
| RO-ST22 | Romania; Podişul Hârtibaciului Mts., Vânători village, dry pasture; 46°13´35´´N, 24°58´46´´E; 399 m; 12 Aug. 2011; leg. JK & MS | 10 | 10 | - |
| RO-ST23 | Romania; Munţii Baraolt Mts., Vâlcele village, pasture; 45°50´57´´N, 25°43´14´´E; 399 m; 13 Aug. 2011; leg. JK & MS | 10 | 10 | - |
| RO-ST24 | Romania; Munţii Piatra Mare Mts., Săcele village, dry pasture; 45°35´37´´N, 25°44´54´´E; 664 m; 13 Aug. 2011; leg. JK & MS | 10 | 10 | - |
| RO-ST25 | Romania; Munţii Ciucaş Mts., Pasul Bratoceasadle, forest clearings; 45°28´49´´N, 25°53´50´´E; 1273 m; 13 Aug. 2011; leg. JK & MS | 10 | 10 | - |
| RO-ST26 | Romania; Munţii Monteoru Mts., Haleş village, dry meadow; 45°14´05´´N, 26°32´44´´E; 428 m; 13 Aug. 2012; leg. JK & MS | 10 | 10 | - |
| RO-ST27 | Romania; Munţii Vrancei Mts., Poduri village, orchard; 45°53´05´´N, 26°49´55´´E; 323 m; 13 Aug. 2011; leg. JK & MS | 10 | 10 | - |
| RO-ST28 | Romania; Munţii Vrancei Mts., saddle above Greşu village, mountain meadow; 45°58´27´´N, 26°26´40´´E; 987 m; 13 Aug. 2011; leg. JK & MS | 10 | 10 | - |
| RO-ST29 | Romania; Munţii Nemira Mts., Pasul Oituzsadle, mountain meadow; 46°03´31´´N, 26°21´16´´E; 871 m; 14 Aug. 2011; leg. JK & MS | 10 | 10 | - |
| RO-ST30 | Romania; Munţii Goşmanului Mts., Zemeş village, old orchard; 46°32´22´´N, 26°28´01´´E; 566 m; 14 Aug. 2011; leg. JK & MS | 10 | 10 | - |
| RO-ST31 | Romania; Munţii Stânişoara Mts., Poiana Crăcăoani Mt., ruderal places near stream; 47°03´37´´N, 26°17´05´´E; 606 m; 14 Aug. 2011; leg. JK & MS | 10 | 10 | - |
| RO-ST32 | Romania; Munţii Stânişoara Mts., near Gura Humorului town, pasture; 47°29´50´´N, 25°52´41´´E; 565 m; 14 Aug. 2011; leg. JK & MS | 10 | 10 | - |
| RO-ST33 | Romania; Munţii Giumalău Mts., Pasul Mestecaniş sadle, mountain meadow; 47°27´52´´N, 25°20´49´´E; 1102 m; 14 Aug. 2012; leg. JK & MS | 10 | 10 | - |
| RO-ST34 | Romania; Munţii Călimani Mts., near GuraHaiţii village, ruderal meadow; 47°07´15´´N, 25°15´10´´E; 1407 m; 15 Aug. 2011; leg. JK & MS | 9 | 9 | - |
| RO-ST35 | Romania; Munţii Bârgăului Mts., Poiana Stampei Mt., meadow; 47°15´59´´N, 25°01´12´´E; 1200 m; 15 Aug. 2011; leg. JK & MS | 10 | 10 | - |
| RO-ST36 | Romania; Munţii Bârgăului Mts., Cepari Mt., wet meadow; 47°14´28´´N, 24°25´33´´E; 349 m; 15 Aug. 2011; leg. JK & MS | 10 | 10 | - |
| RO-ST37 | Romania; Culmea Brezei Mts., Poiana Blenchii Mt., Cheile Babei canyon, dry pasture; 47°19´06´´N, 23°44´50´´E; 275 m; 15 Aug. 2011; leg. JK & MS | 10 | 10 | - |
| RO-ST38 | Romania; Munţii Gutâi Mts., Izvoarele sky centre, meadow; 47°45´06´´N, 23°43´08´´E; 925 m; 15 Aug. 2011; leg. JK & MS | 10 | 10 | - |
| RO-ST39 | Romania; Munţii Cozia Mts., near Dângeşti village, edge of forest; 45°18´52´´N, 24°22´33´´E; 1047 m; 29 Aug. 2011; leg. BŠ | 9 | 9 | - |
| RO-ST40 | Romania; Munţii Căpăţinii Mts., near Malaia village, forest clearings; 45°20´19´´N, 24°00´52´´E; 850 m; 28 Aug. 2011; leg. BŠ | 10 | 10 | - |
| RO-ST41 | Romania; Munţii Retezat Mts., Lacul Gura Apelor lake, ruderal meadow; 45°19´54´´N, 22°43´31´´E; 1096 m; 27 Aug. 2011; leg. BŠ | 9 | 9 | - |
|  | **Total number** | **995** | **1002** | **22** |

**SUPPLEMENTARY TABLE S2** Details on analyses performed on *S. graminea* populations in the study. Locality code: the country code as reported in the Supplementary Table S1; Individual number: the number of an individual within population; IRS: the internal reference standard (B – *Bellis perennis* L., G – *Glycine max* ‘Polanka’); Ploidy level: 2 – DNA diploid, 3 – DNA triploid and 4 – DNA tetraploid, * indicates populations including tetraploid individuals (‘peculiar tetraploids’) having different absolute genome size overlapping in FCM histograms with peaks of *Bellis perennis*; RGS (a.u.): the relative genome size given in arbitrary units; CVs: B –the coefficient of variation in internal reference standard; CVsa: the coefficient of variation of *S. graminea* sample; SP: the seed production, + – individual with proved seed production, NA – not applicable – the individual in which was not possible unambiguously verify the seed formation; SEX: 0 – code for the individual with male-sterile flowers: 1 – code for the individual with hermaphrodite flowers, ^!^ indicates plants having both sterile and fertile anthers within single flower (gynomonoecy); PWDPG: the percentage of well-developed pollen grains, the dash indicates the individual in which it was not possible to estimate pollen formation.

| **Locality Code** | **Individual number** | **IRS** | **Ploidy level** | **RGS (a.u.)** | | **CVst** | **CVsa** | **SP** | **SEX** | **PWDPG** |
| --- | --- | --- | --- | --- | --- | --- | --- | --- | --- | --- |
|  | | | | **(2n)** | **(n)** |  | | | | |
| SK-ST1 | 2 | B | 4 | 1.15 | 0.29 | 1.2 | 2.76 | + | 1 | 28 |
|  | 4 | B | 4 | 1.18 | 0.29 | 1.36 | 4.05 | NA | 1 | 93 |
|  | 5 | B | 4 | 1.16 | 0.29 | 1.4 | 2.85 | + | 1 | 88 |
|  | 6 | B | 4 | 1.13 | 0.28 | 1.41 | 2.15 | NA | 1 | 80 |
|  | 9 | B | 4 | 1.17 | 0.29 | 1.41 | 2.69 | + | 0 | - |
|  | 10 | B | 4 | 1.17 | 0.29 | 1.41 | 3.4 | NA | 0 | - |
| SK-ST2 | 1 | B | 2 | 0.62 | 0.31 | 1.42 | 3.03 | + | 1 | 40 |
|  | 2 | B | 2 | 0.6 | 0.3 | 1.43 | 3.63 | + | 1 | 41 |
|  | 3 | B | 2 | 0.59 | 0.3 | 1.43 | 3.3 | + | 1 | 46 |
|  | 4 | B | 2 | 0.59 | 0.3 | 1.44 | 3.16 | + | 1 | 48 |
|  | 5 | B | 2 | 0.59 | 0.3 | 1.44 | 3.75 | + | 1 | 49 |
|  | 6 | B | 2 | 0.6 | 0.3 | 1.45 | 3.5 | + | 1 | 52 |
|  | 7 | B | 2 | 0.61 | 0.31 | 1.45 | 4.05 | + | 1 | 56 |
|  | 8 | B | 2 | 0.61 | 0.3 | 1.46 | 3.63 | + | 1 | 58 |
|  | 9 | B | 2 | 0.61 | 0.3 | 1.47 | 3.73 | + | 1 | 59 |
|  | 10 | B | 2 | 0.6 | 0.3 | 1.47 | 3.23 | + | 1 | 59 |
| SK-ST3 | 1 | B | 4 | 1.15 | 0.29 | 1.47 | 3.18 | + | 1 | 81 |
|  | 2 | G | 4* | 1.45 | 0.35 | 1.48 | 3.4 | NA | 1 | 68 |
|  | 3 | B | 4 | 1.18 | 0.3 | 1.48 | 2.83 | + | 1 | 81 |
|  | 4 | B | 4 | 1.17 | 0.29 | 1.48 | 2.86 | NA | 0 | 80 |
|  | 5 | B | 4 | 1.15 | 0.29 | 1.49 | 3.51 | + | 0 | 0 |
|  | 6 | G | 4* | 1.5 | 0.36 | 1.5 | 2.33 | + | 1 | 79 |
|  | 7 | B | 4 | 1.14 | 0.28 | 1.5 | 2.97 | NA | 0 | 0 |
|  | 8 | B | 4 | 1.16 | 0.29 | 1.5 | 3.27 | + | 1 | 81 |
|  | 9 | B | 4 | 1.19 | 0.3 | 1.51 | 3.17 | NA | 1 | - |
|  | 10 | B | 4 | 1.15 | 0.29 | 1.51 | 4.03 | NA | 0 | 0 |
| SK-ST4 | 1 | B | 4 | 1.19 | 0.3 | 1.51 | 4.11 | NA | 1 | 82 |
|  | 2 | B | 4 | 1.15 | 0.29 | 1.51 | 3.16 | NA | 1 | 92 |
|  | 3 | B | 4 | 1.13 | 0.28 | 1.52 | 3.15 | NA | 1 | - |
|  | 4 | B | 4 | 1.13 | 0.28 | 1.53 | 3.09 | + | 1 | 92 |
|  | 5 | B | 4 | 1.15 | 0.29 | 1.53 | 3.09 | + | 1 | - |
|  | 6 | B | 4 | 1.12 | 0.28 | 1.54 | 4.02 | + | 1 | 84 |
|  | 7 | B | 4 | 1.13 | 0.28 | 1.54 | 3.61 | NA | 1 | 96 |
|  | 8 | B | 4 | 1.11 | 0.28 | 1.54 | 1.83 | NA | 1 | 92 |
|  | 9 | B | 4 | 1.19 | 0.3 | 1.56 | 2.47 | + | 1 | 98 |
|  | 10 | B | 4 | 1.12 | 0.28 | 1.57 | 3.31 | + | 1 | 97 |
| SK-ST5 | 1 | B | 2 | 0.6 | 0.3 | 1.58 | 3.69 | + | 1 | 61 |
|  | 2 | B | 2 | 0.62 | 0.31 | 1.59 | 3.02 | + | 1 | 62 |
|  | 3 | B | 2 | 0.6 | 0.3 | 1.59 | 3.9 | + | 1 | 62 |
|  | 4 | B | 2 | 0.6 | 0.3 | 1.6 | 3.74 | + | 1 | 63 |
|  | 5 | B | 2 | 0.6 | 0.3 | 1.6 | 3.93 | + | 1 | 63 |
|  | 6 | B | 2 | 0.6 | 0.3 | 1.61 | 3.72 | + | 1 | 63 |
|  | 7 | B | 2 | 0.6 | 0.3 | 1.61 | 3.25 | NA | 1 | 63 |
|  | 8 | B | 2 | 0.6 | 0.3 | 1.62 | 4.1 | + | 1 | 64 |
|  | 9 | B | 4 | 1.13 | 0.28 | 1.62 | 1.92 | + | 0 | 0 |
|  | 10 | B | 2 | 0.6 | 0.3 | 1.62 | 3.35 | + | 1 | 65 |
| SK-ST6 | 1 | G | 4* | 1.08 | 0.36 | 1.63 | 1.3 | + | 1 | - |
|  | 2 | G | 4* | 1.08 | 0.36 | 1.63 | 1.3 | NA | 0 | - |
|  | 3 | G | 4* | 1.08 | 0.36 | 1.65 | 1.3 | NA | 0 | - |
|  | 4 | G | 4* | 1.08 | 0.36 | 1.65 | 1.3 | + | 0 | 0 |
|  | 5 | G | 4* | 1.07 | 0.36 | 1.65 | 1.62 | + | 0 | - |
|  | 6 | G | 4* | 1.07 | 0.36 | 1.65 | 1.62 | NA | 0 | 0 |
|  | 7 | G | 4* | 1.07 | 0.36 | 1.66 | 1.62 | + | 0 | 0 |
|  | 8 | G | 4* | 1.07 | 0.36 | 1.66 | 1.62 | + | 1 | 81 |
|  | 9 | G | 4* | 1.07 | 0.36 | 1.66 | 1.7 | NA | 0 | - |
|  | 10 | G | 4 | 1.07 | 0.36 | 1.66 | 1.7 | + | 0 | 0 |
| SK-ST7 | 1 | B | 4 | 1.14 | 0.28 | 1.68 | 3.02 | + | 0 | 0 |
|  | 2 | B | 4 | 1.16 | 0.29 | 1.68 | 3.98 | + | 1 | - |
|  | 3 | B | 4 | 1.16 | 0.29 | 1.68 | 3.02 | + | 1! | 75 |
|  | 4 | B | 4 | 1.15 | 0.29 | 1.69 | 1.81 | + | 1 | 85 |
|  | 5 | B | 4 | 1.16 | 0.29 | 1.69 | 3.34 | + | 1 | 80 |
|  | 6 | B | 4 | 1.17 | 0.29 | 1.7 | 3.32 | + | 1 | 72 |
|  | 7 | B | 4 | 1.17 | 0.29 | 1.7 | 3.2 | + | 1 | - |
|  | 8 | B | 4 | 1.12 | 0.28 | 1.7 | 2.76 | + | 1 | 95 |
|  | 9 | B | 4 | 1.15 | 0.29 | 1.7 | 3 | + | 1 | 89 |
|  | 10 | B | 4 | 1.17 | 0.29 | 1.71 | 3.01 | + | 1 | 77 |
| SK-ST8 | 1 | B | 4 | 1.14 | 0.29 | 1.71 | 3.14 | + | 0 | - |
|  | 2 | B | 4 | 1.15 | 0.29 | 1.71 | 2.86 | + | 1 | 69 |
|  | 3 | B | 4 | 1.15 | 0.29 | 1.72 | 3.23 | NA | 1 | 93 |
|  | 4 | B | 4 | 1.16 | 0.29 | 1.72 | 3.72 | + | 0 | - |
|  | 5 | B | 4 | 1.14 | 0.29 | 1.72 | 3.21 | + | 0 | 0 |
|  | 6 | B | 4 | 1.13 | 0.28 | 1.72 | 2.65 | + | 1 | - |
|  | 7 | B | 4 | 1.16 | 0.29 | 1.72 | 3.73 | + | 1 | - |
|  | 8 | B | 4 | 1.14 | 0.29 | 1.72 | 2.9 | + | 1 | 94 |
|  | 9 | B | 4 | 1.13 | 0.28 | 1.73 | 2.99 | NA | 0 | 0 |
|  | 10 | B | 4 | 1.15 | 0.29 | 1.73 | 3.4 | + | 1 | 95 |
| SK-ST9 | 2 | B | 4 | 1.13 | 0.28 | 1.73 | 2.18 | + | 1 | 86 |
|  | 3 | B | 4 | 1.11 | 0.28 | 1.73 | 2.82 | + | 1 | - |
|  | 4 | G | 4* | 1.09 | 0.36 | 1.73 | 1.6 | NA | 1 | - |
|  | 5 | G | 4* | 1.08 | 0.36 | 1.73 | 2.99 | NA | 1 | 92 |
|  | 6 | B | 4 | 1.09 | 0.36 | 1.73 | 2.43 | + | 1 | 81 |
|  | 7 | G | 4* | 1.08 | 0.36 | 1.74 | 1.25 | + | 1 | 82 |
|  | 8 | B | 4 | 1.1 | 0.28 | 1.74 | 2.72 | + | 1 | - |
|  | 9 | G | 4* | 1.08 | 0.36 | 1.74 | 3.5 | NA | 1 | 91 |
|  | 10 | B | 4 | 1.14 | 0.28 | 1.74 | 1.86 | NA | 1 | 93 |
| SK-ST10 | 1 | B | 4 | 1.15 | 0.29 | 1.75 | 3.51 | + | 1 | 90 |
|  | 2 | B | 4 | 1.15 | 0.29 | 1.75 | 3.31 | + | 1 | 95 |
|  | 3 | B | 4 | 1.17 | 0.29 | 1.75 | 3.65 | + | 1 | 96 |
|  | 4 | B | 4 | 1.16 | 0.29 | 1.75 | 3.87 | + | 0 | 0 |
|  | 5 | B | 4 | 1.15 | 0.29 | 1.75 | 3.28 | + | 1 | 97 |
|  | 6 | B | 4 | 1.17 | 0.29 | 1.76 | 3.17 | + | 1 | 87 |
|  | 7 | B | 4 | 1.14 | 0.29 | 1.76 | 2.6 | + | 1 | 96 |
|  | 8 | B | 4 | 1.13 | 0.28 | 1.76 | 1.9 | + | 1 | 93 |
|  | 9 | B | 4 | 1.17 | 0.29 | 1.76 | 3.56 | + | 1 | 55 |
|  | 10 | B | 4 | 1.15 | 0.29 | 1.76 | 3.01 | NA | 1 | 84 |
| SK-ST11 | 1 | B | 4 | 1.13 | 0.28 | 1.77 | 4.04 | + | 0 | - |
|  | 2 | B | 4 | 1.15 | 0.29 | 1.77 | 1.86 | + | 1 | 94 |
|  | 3 | B | 4 | 1.13 | 0.28 | 1.77 | 2.99 | NA | 0 | 0 |
|  | 4 | B | 4 | 1.19 | 0.3 | 1.77 | 1.83 | NA | 1 | 93 |
|  | 5 | B | 4 | 1.12 | 0.28 | 1.77 | 2.05 | + | 1 | - |
|  | 6 | B | 4 | 1.13 | 0.28 | 1.77 | 3.09 | + | 1 | 78 |
|  | 7 | B | 4 | 1.13 | 0.28 | 1.77 | 2.69 | NA | 1 | 80 |
|  | 8 | B | 4 | 1.17 | 0.29 | 1.77 | 3.47 | + | 0 | - |
|  | 9 | B | 4 | 1.13 | 0.28 | 1.78 | 2.86 | + | 1 | 91 |
|  | 10 | B | 4 | 1.15 | 0.29 | 1.78 | 3.25 | + | 0 | 0 |
| SK-ST12 | 1 | B | 4 | 1.12 | 0.28 | 1.78 | 2.02 | NA | 1 | 94 |
|  | 2 | B | 4 | 1.16 | 0.29 | 1.79 | 4.09 | + | 0 | 0 |
|  | 3 | B | 4 | 1.11 | 0.28 | 1.79 | 2.77 | + | 1 | 92 |
|  | 4 | B | 4 | 1.15 | 0.29 | 1.79 | 2.04 | + | 1 | 88 |
|  | 5 | B | 4 | 1.16 | 0.29 | 1.79 | 2.32 | NA | 1 | 97 |
|  | 6 | B | 4 | 1.17 | 0.29 | 1.8 | 3.25 | NA | 1 | 94 |
|  | 7 | B | 4 | 1.18 | 0.29 | 1.8 | 3.3 | + | 1 | 94 |
|  | 8 | B | 4 | 1.19 | 0.3 | 1.8 | 3.65 | + | 1 | 84 |
|  | 9 | B | 4 | 1.11 | 0.28 | 1.81 | 1.98 | + | 1 | 92 |
|  | 10 | B | 4 | 1.1 | 0.27 | 1.81 | 2.45 | + | 1 | 88 |
| SK-ST13 | 1 | B | 4 | 1.15 | 0.29 | 1.82 | 2.84 | + | 1 | 86 |
|  | 2 | B | 4 | 1.13 | 0.28 | 1.82 | 2.48 | NA | 0 | 0 |
|  | 3 | B | 4 | 1.16 | 0.29 | 1.82 | 1.78 | + | 1 | 72 |
|  | 4 | B | 4 | 1.16 | 0.29 | 1.82 | 3.11 | + | 1 | 96 |
|  | 5 | B | 4 | 1.19 | 0.3 | 1.82 | 3.12 | + | 1 | 84 |
|  | 6 | B | 4 | 1.17 | 0.29 | 1.82 | 2.46 | + | 1 | 97 |
|  | 7 | B | 4 | 1.14 | 0.28 | 1.82 | 3.32 | + | 1 | 91 |
|  | 8 | B | 4 | 1.15 | 0.29 | 1.82 | 3.15 | NA | 1 | 96 |
|  | 9 | B | 4 | 1.12 | 0.28 | 1.82 | 2.58 | NA | 1 | 95 |
|  | 10 | B | 4 | 1.16 | 0.29 | 1.83 | 2.61 | NA | 1 | 64 |
| SK-ST14 | 1 | B | 4 | 1.17 | 0.29 | 1.83 | 2.54 | + | 1 | 70 |
|  | 2 | B | 4 | 1.15 | 0.29 | 1.83 | 1.66 | NA | 0 | 0 |
|  | 3 | B | 4 | 1.15 | 0.29 | 1.83 | 2.21 | NA | 1 | 32 |
|  | 4 | B | 4 | 1.15 | 0.29 | 1.84 | 2.77 | NA | 1 | 90 |
|  | 5 | B | 4 | 1.14 | 0.29 | 1.84 | 1.71 | NA | 1 | 89 |
|  | 6 | B | 4 | 1.16 | 0.29 | 1.84 | 1.73 | NA | 0 | 0 |
|  | 7 | B | 4 | 1.13 | 0.28 | 1.84 | 3.31 | + | 1 | 77 |
|  | 8 | B | 4 | 1.15 | 0.29 | 1.84 | 2.3 | + | 1 | 87 |
|  | 9 | B | 4 | 1.14 | 0.29 | 1.84 | 2.65 | + | 1 | - |
|  | 10 | B | 4 | 1.12 | 0.28 | 1.84 | 3.05 | + | 1 | 96 |
| SK-ST15 | 1 | B | 4 | 1.16 | 0.29 | 1.84 | 2.04 | + | 0 | 0 |
|  | 2 | B | 4 | 1.16 | 0.29 | 1.85 | 1.93 | + | 0 | 0 |
|  | 3 | B | 4 | 1.19 | 0.3 | 1.85 | 3.67 | NA | 1 | 93 |
|  | 4 | B | 4 | 1.14 | 0.28 | 1.85 | 3.05 | + | 1 | 82 |
|  | 5 | B | 4 | 1.16 | 0.29 | 1.86 | 2.4 | + | 0 | 0 |
|  | 6 | B | 4 | 1.14 | 0.29 | 1.86 | 2.4 | + | 1 | 73 |
|  | 7 | B | 4 | 1.15 | 0.29 | 1.86 | 2.38 | + | 0 | 0 |
|  | 8 | B | 4 | 1.17 | 0.29 | 1.86 | 2.84 | NA | 0 | 0 |
|  | 9 | B | 4 | 1.14 | 0.29 | 1.86 | 2.8 | NA | 0 | 0 |
|  | 10 | B | 4 | 1.13 | 0.28 | 1.86 | 2.36 | + | 1 | 93 |
| SK-ST16 | 1 | B | 4 | 1.11 | 0.28 | 1.86 | 2.69 | + | 1 | 43 |
|  | 2 | B | 4 | 1.16 | 0.29 | 1.87 | 2.04 | + | 1 | 69 |
|  | 3 | B | 4 | 1.13 | 0.28 | 1.87 | 2.29 | + | 1 | 92 |
|  | 4 | B | 4 | 1.16 | 0.29 | 1.87 | 1.86 | + | 1 | 32 |
|  | 5 | B | 4 | 1.15 | 0.29 | 1.87 | 2.08 | + | 1 | 95 |
|  | 6 | B | 4 | 1.14 | 0.29 | 1.87 | 3.08 | + | 1 | 80 |
|  | 7 | B | 4 | 1.12 | 0.28 | 1.88 | 2.71 | + | 1 | 70 |
|  | 8 | B | 2 | 0.62 | 0.31 | 1.88 | 2.94 | NA | 0 | 0 |
|  | 9 | B | 4 | 1.15 | 0.29 | 1.88 | 2.3 | + | 1 | - |
|  | 10 | B | 4 | 1.17 | 0.29 | 1.88 | 1.9 | + | 1 | 52 |
| SK-ST17 | 1 | B | 2 | 0.61 | 0.31 | 1.88 | 2 | NA | 1 | 65 |
|  | 2 | B | 4 | 1.13 | 0.28 | 1.88 | 2.51 | + | 1 | - |
|  | 3 | B | 4 | 1.18 | 0.3 | 1.89 | 2.2 | + | 1 | 57 |
|  | 4 | B | 4 | 1.15 | 0.29 | 1.89 | 2.66 | NA | 1 | 80 |
|  | 5 | B | 4 | 1.16 | 0.29 | 1.89 | 2.47 | + | 1 | 72 |
|  | 6 | B | 4 | 1.17 | 0.29 | 1.9 | 3.08 | + | 1 | 98 |
|  | 7 | B | 4 | 1.25 | 0.3 | 1.9 | 2.74 | + | 1 | 62 |
|  | 8 | B | 4 | 1.18 | 0.3 | 1.9 | 2.22 | + | 1 | 90 |
|  | 9 | B | 4 | 1.2 | 0.3 | 1.91 | 2.55 | + | 1 | - |
|  | 10 | B | 4 | 1.19 | 0.3 | 1.91 | 2.73 | + | 1 | 69 |
| SK-ST18 | 1 | B | 4 | 1.13 | 0.28 | 1.91 | 3.33 | + | 1 | 86 |
|  | 2 | B | 4 | 1.15 | 0.29 | 1.91 | 2.38 | + | 0 | 0 |
|  | 3 | B | 4 | 1.15 | 0.29 | 1.92 | 1.57 | + | 1 | 76 |
|  | 4 | B | 4 | 1.16 | 0.29 | 1.92 | 2.14 | + | 0 | 0 |
|  | 5 | B | 4 | 1.18 | 0.29 | 1.92 | 3.82 | + | 1 | 53 |
|  | 6 | B | 4 | 1.15 | 0.29 | 1.92 | 2.05 | + | 1 | 98 |
|  | 7 | B | 4 | 1.16 | 0.29 | 1.93 | 3.09 | + | 1 | 94 |
|  | 8 | B | 4 | 1.16 | 0.29 | 1.93 | 2.1 | + | 1 | 88 |
|  | 9 | B | 4 | 1.16 | 0.29 | 1.93 | 3.33 | NA | 0 | 0 |
|  | 10 | B | 4 | 1.16 | 0.29 | 1.93 | 2.23 | NA | 0 | 0 |
| SK-ST19 | 1 | B | 4 | 1.14 | 0.29 | 1.93 | 2.76 | + | 1 | 86 |
|  | 2 | B | 4 | 1.15 | 0.29 | 1.93 | 2.63 | + | 1 | 80 |
|  | 3 | B | 4 | 1.15 | 0.29 | 1.94 | 2.63 | + | 1 | 94 |
|  | 4 | B | 4 | 1.16 | 0.29 | 1.94 | 2.94 | + | 1 | 54 |
|  | 5 | B | 4 | 1.14 | 0.29 | 1.94 | 2.6 | + | 1 | 53 |
|  | 6 | B | 4 | 1.15 | 0.29 | 1.95 | 2.36 | + | 1 | 68 |
|  | 7 | B | 4 | 1.17 | 0.29 | 1.95 | 3.07 | NA | 1 | 54 |
|  | 8 | B | 4 | 1.18 | 0.29 | 1.95 | 3.48 | + | 1 | 93 |
|  | 9 | B | 4 | 1.16 | 0.29 | 1.95 | 2.88 | NA | 1 | 38 |
|  | 10 | B | 2 | 0.61 | 0.3 | 1.96 | 2.84 | + | 1 | 66 |
| SK-ST20 | 1 | B | 4 | 1.16 | 0.29 | 1.96 | 3.7 | NA | 1 | 70 |
|  | 2 | B | 4 | 1.15 | 0.29 | 1.96 | 3.6 | + | 1 | 83 |
|  | 3 | B | 4 | 1.15 | 0.29 | 1.96 | 3.25 | NA | 1 | 29 |
|  | 4 | B | 4 | 1.16 | 0.29 | 1.96 | 3.25 | + | 1 | 78 |
|  | 5 | B | 4 | 1.14 | 0.29 | 1.96 | 3.02 | + | 1 | 72 |
|  | 6 | B | 4 | 1.15 | 0.29 | 1.96 | 2.76 | NA | 1 | 84 |
|  | 7 | B | 4 | 1.16 | 0.29 | 1.96 | 3.3 | NA | 1 | 86 |
|  | 8 | B | 4 | 1.16 | 0.29 | 1.97 | 1.94 | + | 1 | 91 |
|  | 9 | B | 4 | 1.19 | 0.3 | 1.97 | 2.06 | + | 0 | 0 |
|  | 10 | B | 4 | 1.17 | 0.29 | 1.97 | 3.62 | + | 1 | 72 |
| SK-ST21 | 1 | B | 4 | 1.17 | 0.29 | 1.97 | 2.34 | + | 1 | 86 |
|  | 2 | B | 4 | 1.16 | 0.29 | 1.97 | 3.33 | + | 1 | 79 |
|  | 3 | B | 4 | 1.17 | 0.29 | 1.97 | 3.42 | + | 1 | - |
|  | 4 | B | 4 | 1.17 | 0.29 | 1.97 | 3.36 | + | 0 | - |
|  | 5 | B | 4 | 1.18 | 0.29 | 1.98 | 2.98 | + | 0 | 0 |
|  | 6 | B | 4 | 1.17 | 0.29 | 1.98 | 3.24 | + | 1 | 80 |
|  | 7 | B | 4 | 1.17 | 0.29 | 1.98 | 1.84 | + | 1 | 68 |
|  | 8 | B | 4 | 1.17 | 0.29 | 1.98 | 3.69 | + | 1 | - |
|  | 9 | B | 4 | 1.17 | 0.29 | 1.98 | 3.14 | NA | 1 | 60 |
|  | 10 | B | 4 | 1.17 | 0.29 | 1.99 | 3.9 | NA | 1 | 92 |
| SK-ST22 | 1 | B | 4 | 1.13 | 0.28 | 1.99 | 2.56 | NA | 1 | 96 |
|  | 2 | B | 4 | 1.15 | 0.29 | 1.99 | 2.25 | + | 1 | 86 |
|  | 3 | B | 4 | 1.15 | 0.29 | 1.99 | 2.08 | NA | 1 | - |
|  | 5 | B | 4 | 1.14 | 0.29 | 1.99 | 1.98 | + | 0 | 0 |
|  | 6 | B | 4 | 1.16 | 0.29 | 1.99 | 2.19 | + | 1! | - |
|  | 7 | B | 4 | 1.18 | 0.3 | 1.99 | 4.32 | NA | 0 | 0 |
|  | 8 | B | 4 | 1.16 | 0.29 | 1.99 | 1.86 | + | 0 | 0 |
|  | 9 | B | 4 | 1.14 | 0.29 | 1.99 | 3.06 | + | 1 | 76 |
|  | 10 | B | 4 | 1.18 | 0.3 | 1.99 | 2.52 | + | 1 | 91 |
| SK-ST23 | 1 | B | 4 | 1.14 | 0.29 | 1.99 | 1.54 | + | 1 | 88 |
|  | 2 | B | 4 | 1.13 | 0.28 | 1.99 | 2.38 | + | 1 | 94 |
|  | 3 | B | 4 | 1.16 | 0.29 | 2 | 3.08 | + | 0 | 0 |
|  | 4 | B | 2 | 0.61 | 0.3 | 2.01 | 3.32 | + | 1 | 67 |
|  | 5 | B | 4 | 1.15 | 0.29 | 2.01 | 2.71 | + | 1 | 90 |
|  | 6 | B | 2 | 0.61 | 0.31 | 2.01 | 3.56 | + | 1 | 67 |
|  | 7 | B | 2 | 0.63 | 0.31 | 2.01 | 3.73 | NA | 0 | 0 |
|  | 8 | B | 4 | 1.14 | 0.29 | 2.02 | 1.69 | + | 1 | 92 |
|  | 9 | B | 2 | 0.61 | 0.31 | 2.02 | 3.16 | + | 1 | 67 |
|  | 10 | B | 2 | 0.62 | 0.31 | 2.02 | 1.58 | + | 1 | 67 |
| SK-ST24 | 1 | B | 4 | 1.19 | 0.3 | 2.03 | 3.95 | + | 1 | 88 |
|  | 2 | B | 4 | 1.15 | 0.29 | 2.03 | 1.71 | NA | 1 | 92 |
|  | 3 | B | 4 | 1.18 | 0.3 | 2.04 | 2.8 | NA | 1 | 88 |
|  | 4 | B | 4 | 1.17 | 0.29 | 2.05 | 1.67 | NA | 0 | 0 |
|  | 5 | B | 4 | 1.16 | 0.29 | 2.05 | 1.37 | + | 1 | 80 |
|  | 6 | B | 4 | 1.17 | 0.29 | 2.05 | 1.34 | + | 1 | 89 |
|  | 8 | B | 4 | 1.16 | 0.29 | 2.05 | 1.61 | + | 1 | 95 |
|  | 9 | B | 4 | 1.19 | 0.3 | 2.05 | 3.23 | + | 1 | 90 |
|  | 10 | B | 4 | 1.15 | 0.29 | 2.05 | 2.74 | + | 1 | 72 |
| SK-ST25 | 1 | B | 4 | 1.13 | 0.28 | 2.05 | 2.07 | + | 1 | 81 |
|  | 2 | B | 4 | 1.15 | 0.29 | 2.06 | 2.05 | + | 1 | 82 |
|  | 3 | B | 4 | 1.15 | 0.29 | 2.06 | 3.73 | + | 1 | 37 |
|  | 4 | B | 4 | 1.14 | 0.29 | 2.06 | 2.48 | + | 1 | 84 |
|  | 5 | B | 4 | 1.15 | 0.29 | 2.06 | 2.68 | + | 1 | 82 |
|  | 6 | B | 4 | 1.16 | 0.29 | 2.06 | 3.04 | + | 1 | 16 |
|  | 7 | B | 4 | 1.16 | 0.29 | 2.06 | 1.91 | NA | 1 | - |
| SK-ST26 | 1 | B | 2 | 0.61 | 0.3 | 2.07 | 3.06 | NA | 1 | 67 |
|  | 2 | B | 2 | 0.6 | 0.3 | 2.07 | 4.11 | NA | 1 | 68 |
|  | 3 | B | 2 | 0.61 | 0.3 | 2.07 | 3.75 | + | 0 | 0 |
|  | 4 | B | 2 | 0.6 | 0.3 | 2.07 | 3.24 | + | 1 | 68 |
|  | 5 | B | 2 | 0.62 | 0.31 | 2.08 | 3.1 | NA | 1 | 68 |
|  | 6 | B | 4 | 1.19 | 0.3 | 2.08 | 1.99 | + | 1 | 91 |
|  | 7 | B | 2 | 0.61 | 0.3 | 2.08 | 3.02 | + | 1 | 68 |
|  | 8 | B | 2 | 0.62 | 0.31 | 2.08 | 3.75 | + | 1! | 69 |
|  | 9 | B | 2 | 0.61 | 0.3 | 2.09 | 3.56 | + | 1 | 69 |
|  | 10 | B | 2 | 0.61 | 0.3 | 2.09 | 3.17 | + | 1 | 69 |
| SK-ST27 | 1 | B | 4 | 1.16 | 0.29 | 2.09 | 1.99 | + | 1 | 92 |
|  | 2 | B | 4 | 1.18 | 0.29 | 2.09 | 3.34 | + | 1 | 79 |
|  | 3 | B | 4 | 1.17 | 0.29 | 2.09 | 2.99 | + | 1 | 49 |
|  | 4 | B | 4 | 1.18 | 0.29 | 2.1 | 4.45 | + | 1 | 80 |
|  | 5 | B | 4 | 1.16 | 0.29 | 2.1 | 3.57 | NA | 0 | 0 |
|  | 6 | B | 4 | 1.17 | 0.29 | 2.1 | 3.63 | + | 1 | 46 |
|  | 7 | B | 4 | 1.14 | 0.28 | 2.11 | 3.57 | + | 1 | 80 |
|  | 8 | B | 4 | 1.13 | 0.28 | 2.11 | 2.13 | + | 1 | 91 |
|  | 9 | B | 2 | 0.6 | 0.3 | 2.11 | 3.05 | + | 1 | 69 |
|  | 10 | B | 4 | 1.18 | 0.29 | 2.11 | 3.7 | + | 0 | 0 |
| SK-ST28 | 1 | B | 4 | 1.16 | 0.29 | 2.11 | 4.02 | + | 1 | 92 |
|  | 2 | B | 4 | 1.16 | 0.29 | 2.12 | 3.36 | + | 0 | 0 |
|  | 3 | B | 4 | 1.14 | 0.29 | 2.12 | 3.05 | + | 1 | 90 |
|  | 4 | B | 4 | 1.16 | 0.29 | 2.12 | 3.8 | + | 1 | 90 |
|  | 5 | B | 4 | 1.16 | 0.29 | 2.12 | 4.1 | NA | 1 | 64 |
|  | 6 | B | 4 | 1.15 | 0.29 | 2.12 | 3.13 | + | 1 | 80 |
|  | 7 | B | 4 | 1.18 | 0.29 | 2.12 | 3.26 | NA | 1 | 79 |
|  | 8 | B | 4 | 1.14 | 0.29 | 2.13 | 3.37 | + | 1 | 79 |
|  | 9 | B | 4 | 1.15 | 0.29 | 2.13 | 2.95 | + | 1 | 88 |
|  | 10 | B | 4 | 1.13 | 0.28 | 2.13 | 3.49 | + | 1 | 91 |
| SK-ST29 | 1 | B | 4 | 1.16 | 0.29 | 2.13 | 2.24 | + | 1 | 82 |
|  | 2 | B | 4 | 1.16 | 0.29 | 2.13 | 2.25 | + | 1 | 76 |
|  | 3 | B | 4 | 1.13 | 0.28 | 2.14 | 2.58 | + | 1 | 64 |
|  | 4 | B | 4 | 1.14 | 0.29 | 2.14 | 3.95 | + | 1 | 60 |
|  | 5 | B | 4 | 1.14 | 0.28 | 2.14 | 2.26 | + | 1 | 76 |
|  | 6 | B | 4 | 1.14 | 0.29 | 2.15 | 2.95 | NA | 0 | 0 |
|  | 7 | B | 4 | 1.17 | 0.29 | 2.16 | 2.24 | + | 0 | 0 |
|  | 8 | B | 4 | 1.16 | 0.29 | 2.16 | 3.85 | NA | 1 | 80 |
|  | 9 | B | 4 | 1.14 | 0.28 | 2.16 | 3.07 | + | 1 | 89 |
|  | 10 | B | 4 | 1.13 | 0.28 | 2.16 | 2.84 | NA | 1 | 79 |
| SK-ST30 | 1 | B | 4 | 1.12 | 0.28 | 2.17 | 2.99 | + | 0 | - |
|  | 2 | B | 4 | 1.16 | 0.29 | 2.17 | 4.13 | + | 1 | - |
|  | 3 | B | 4 | 1.12 | 0.28 | 2.17 | 2.15 | + | 1 | 82 |
|  | 4 | B | 4 | 1.17 | 0.29 | 2.17 | 3.59 | + | 0 | 0 |
|  | 5 | B | 4 | 1.16 | 0.29 | 2.18 | 3.45 | + | 0 | 0 |
|  | 6 | B | 4 | 1.16 | 0.29 | 2.18 | 2.21 | NA | 1 | 80 |
|  | 7 | B | 4 | 1.11 | 0.28 | 2.18 | 3.24 | + | 0 | 0 |
|  | 8 | B | 4 | 1.13 | 0.28 | 2.18 | 2.26 | + | 1! | - |
|  | 9 | B | 4 | 1.1 | 0.28 | 2.19 | 1.96 | NA | 1 | 72 |
|  | 10 | B | 4 | 1.15 | 0.29 | 2.19 | 3.16 | + | 0 | 0 |
| SK-ST31 | 1 | B | 4 | 1.16 | 0.29 | 2.19 | 4.04 | + | 0 | 0 |
|  | 2 | B | 4 | 1.11 | 0.28 | 2.19 | 1.61 | + | 1 | 89 |
|  | 3 | B | 2 | 0.62 | 0.31 | 2.19 | 3.48 | + | 1 | 69 |
|  | 4 | B | 4 | 1.14 | 0.28 | 2.2 | 2.18 | + | 1 | 88 |
|  | 5 | B | 4 | 1.18 | 0.29 | 2.2 | 2.11 | + | 1 | 72 |
|  | 6 | B | 4 | 1.15 | 0.29 | 2.2 | 2.18 | + | 1 | 92 |
|  | 7 | B | 2 | 0.62 | 0.31 | 2.21 | 3.41 | + | 1 | 70 |
|  | 8 | B | 4 | 1.16 | 0.29 | 2.21 | 2.16 | + | 1 | 89 |
|  | 9 | B | 4 | 1.17 | 0.29 | 2.21 | 2.26 | + | 1 | 80 |
|  | 10 | B | 4 | 1.15 | 0.29 | 2.21 | 3.25 | + | 0 | 0 |
| SK-ST32 | 1 | B | 3 | 0.79 | 0.26 | 2.21 | 2.93 | NA | 1 | - |
|  | 2 | B | 4 | 1.16 | 0.29 | 2.22 | 2.48 | + | 1 | 89 |
|  | 3 | B | 4 | 1.17 | 0.29 | 2.23 | 3.19 | + | 1 | 91 |
|  | 4 | B | 4 | 1.19 | 0.3 | 2.23 | 2.48 | NA | 1 | 80 |
|  | 5 | B | 4 | 1.16 | 0.29 | 2.23 | 2.22 | + | 1 | 91 |
|  | 6 | B | 4 | 1.17 | 0.29 | 2.23 | 2.51 | + | 1 | 82 |
|  | 7 | B | 4 | 1.13 | 0.28 | 2.24 | 2.34 | + | 1 | 80 |
|  | 8 | B | 4 | 1.15 | 0.29 | 2.24 | 3.33 | NA | 1 | 94 |
| SK-ST33 | 1 | B | 4 | 1.15 | 0.29 | 2.24 | 3.04 | + | 1 | 78 |
|  | 2 | B | 4 | 1.12 | 0.28 | 2.24 | 3.3 | + | 1 | 73 |
|  | 3 | B | 4 | 1.17 | 0.29 | 2.25 | 3.75 | + | 1 | 90 |
|  | 4 | B | 4 | 1.13 | 0.28 | 2.25 | 1.72 | + | 1 | 66 |
|  | 5 | B | 4 | 1.17 | 0.29 | 2.25 | 2.44 | + | 1 | 79 |
|  | 6 | B | 4 | 1.18 | 0.3 | 2.25 | 3.09 | + | 1 | 81 |
|  | 7 | B | 4 | 1.15 | 0.29 | 2.25 | 2.18 | + | 1 | 87 |
|  | 8 | B | 4 | 1.16 | 0.29 | 2.25 | 2.45 | + | 1! | - |
|  | 9 | B | 4 | 1.15 | 0.29 | 2.26 | 2.96 | + | 0 | 0 |
|  | 10 | B | 4 | 1.14 | 0.28 | 2.26 | 2.33 | + | 1 | 59 |
| SK-ST34 | 1 | B | 2 | 0.63 | 0.31 | 2.26 | 3.25 | + | 1 | 71 |
|  | 2 | B | 2 | 0.62 | 0.31 | 2.27 | 2.89 | + | 1 | 71 |
|  | 3 | B | 2 | 0.63 | 0.31 | 2.27 | 2.56 | + | 0 | 0 |
|  | 4 | B | 2 | 0.61 | 0.31 | 2.27 | 3.56 | + | 1 | 72 |
|  | 5 | B | 2 | 0.62 | 0.31 | 2.27 | 3.59 | + | 1 | 72 |
|  | 6 | B | 2 | 0.62 | 0.31 | 2.27 | 3.65 | + | 0 | 0 |
|  | 7 | B | 2 | 0.63 | 0.32 | 2.27 | 3.05 | + | 1 | 72 |
|  | 8 | B | 2 | 0.62 | 0.31 | 2.27 | 3.74 | + | 0 | 0 |
|  | 9 | B | 2 | 0.62 | 0.31 | 2.27 | 2.49 | + | 1 | 72 |
|  | 10 | B | 2 | 0.61 | 0.31 | 2.28 | 3.4 | + | 0 | 0 |
| SK-ST35 | 1 | B | 4 | 1.17 | 0.29 | 2.28 | 2 | + | 1 | 83 |
|  | 2 | B | 4 | 1.17 | 0.29 | 2.29 | 1.94 | + | 1 | 82 |
|  | 3 | B | 4 | 1.14 | 0.28 | 2.29 | 2.79 | + | 1 | 79 |
|  | 4 | B | 4 | 1.15 | 0.29 | 2.29 | 1.64 | + | 1 | 91 |
|  | 5 | B | 4 | 1.14 | 0.29 | 2.29 | 2.78 | + | 1 | - |
|  | 6 | B | 4 | 1.14 | 0.29 | 2.29 | 1.94 | + | 0 | 0 |
|  | 7 | B | 4 | 1.15 | 0.29 | 2.29 | 1.93 | + | 1 | 68 |
|  | 8 | B | 4 | 1.16 | 0.29 | 2.29 | 2.57 | + | 0 | 0 |
|  | 9 | B | 4 | 1.13 | 0.28 | 2.29 | 1.49 | NA | 1 | 80 |
|  | 10 | B | 4 | 1.13 | 0.28 | 2.29 | 2.66 | + | 1 | 83 |
| SK-ST36 | 1 | B | 4 | 1.15 | 0.29 | 2.29 | 3.61 | + | 1 | - |
|  | 2 | B | 4 | 1.16 | 0.29 | 2.3 | 2.67 | + | 1 | 89 |
|  | 3 | B | 4 | 1.16 | 0.29 | 2.3 | 3.5 | NA | 1 | 54 |
|  | 4 | B | 4 | 1.14 | 0.29 | 2.3 | 3.04 | + | 0 | 0 |
|  | 5 | B | 4 | 1.12 | 0.28 | 2.3 | 2.7 | + | 1 | 82 |
|  | 6 | B | 4 | 1.16 | 0.29 | 2.31 | 3.71 | + | 1 | 63 |
|  | 7 | B | 4 | 1.15 | 0.29 | 2.31 | 2.92 | + | 0 | 0 |
|  | 8 | B | 4 | 1.15 | 0.29 | 2.31 | 3.11 | + | 1 | 88 |
|  | 9 | B | 4 | 1.17 | 0.29 | 2.31 | 4.2 | NA | 0 | 0 |
|  | 10 | B | 4 | 1.19 | 0.3 | 2.31 | 2.1 | + | 1 | 74 |
| SK-ST37 | 1 | B | 4 | 1.12 | 0.28 | 2.31 | 2.77 | + | 1 | 84 |
|  | 2 | B | 4 | 1.13 | 0.28 | 2.31 | 2.65 | + | 0 | 0 |
|  | 3 | B | 4 | 1.13 | 0.28 | 2.31 | 2.45 | + | 1 | - |
|  | 4 | B | 4 | 1.15 | 0.29 | 2.31 | 4.11 | + | 0 | 0 |
|  | 5 | B | 4 | 1.16 | 0.29 | 2.32 | 2.42 | + | 0 | 0 |
|  | 6 | B | 4 | 1.13 | 0.28 | 2.32 | 2.45 | + | 1 | 82 |
|  | 7 | B | 4 | 1.12 | 0.28 | 2.33 | 2.77 | + | 1 | 59 |
|  | 8 | B | 4 | 1.14 | 0.28 | 2.33 | 2.3 | + | 1 | 62 |
|  | 9 | B | 4 | 1.17 | 0.29 | 2.33 | 2.34 | + | 1 | 83 |
|  | 10 | B | 4 | 1.14 | 0.28 | 2.33 | 2.67 | + | 1 | 91 |
| SK-ST38 | 1 | B | 4 | 1.17 | 0.29 | 2.33 | 4.09 | NA | 0 | 0 |
|  | 2 | B | 4 | 1.12 | 0.28 | 2.33 | 2.43 | + | 0 | 0 |
|  | 3 | B | 4 | 1.15 | 0.29 | 2.33 | 2.73 | + | 0 | - |
|  | 4 | B | 4 | 1.16 | 0.29 | 2.34 | 3.55 | NA | 0 | 0 |
|  | 5 | B | 4 | 1.16 | 0.29 | 2.34 | 2.87 | + | 1 | 92 |
|  | 6 | B | 4 | 1.15 | 0.29 | 2.34 | 2.65 | + | 0 | 0 |
|  | 7 | B | 4 | 1.17 | 0.29 | 2.35 | 3.57 | NA | 0 | 0 |
|  | 8 | B | 4 | 1.17 | 0.29 | 2.35 | 3 | NA | 0 | 0 |
|  | 9 | B | 4 | 1.14 | 0.29 | 2.35 | 3.83 | NA | 0 | - |
|  | 10 | B | 4 | 1.16 | 0.29 | 2.36 | 2.42 | NA | 0 | 0 |
| SK-ST39 | 1 | B | 4 | 1.15 | 0.29 | 2.36 | 2.6 | NA | 1 | 82 |
|  | 2 | B | 4 | 1.16 | 0.29 | 2.36 | 2 | + | 1 | 90 |
|  | 3 | B | 4 | 1.13 | 0.28 | 2.36 | 2.28 | NA | 1 | 72 |
|  | 4 | B | 4 | 1.15 | 0.29 | 2.36 | 2.52 | + | 1 | 88 |
|  | 5 | B | 4 | 1.13 | 0.28 | 2.36 | 2.69 | NA | 0 | 0 |
|  | 6 | B | 4 | 1.13 | 0.28 | 2.36 | 2.76 | NA | 1 | 91 |
|  | 7 | B | 4 | 1.16 | 0.29 | 2.36 | 3.22 | NA | 0 | 0 |
|  | 8 | B | 4 | 1.16 | 0.29 | 2.36 | 3.12 | NA | 0 | 0 |
|  | 9 | B | 4 | 1.16 | 0.29 | 2.37 | 2.55 | + | 1 | 87 |
|  | 10 | B | 2 | 0.61 | 0.31 | 2.37 | 4.11 | + | 1! | 72 |
| SK-ST40 | 1 | B | 4 | 1.15 | 0.29 | 2.37 | 2.87 | + | 1 | 65 |
|  | 2 | B | 4 | 1.17 | 0.29 | 2.37 | 2.53 | NA | 1 | 92 |
|  | 3 | B | 4 | 1.19 | 0.3 | 2.37 | 4.05 | NA | 1 | 80 |
|  | 4 | B | 4 | 1.16 | 0.29 | 2.37 | 3.88 | NA | 1 | 73 |
|  | 5 | B | 4 | 1.13 | 0.28 | 2.38 | 3.03 | NA | 1 | 80 |
|  | 6 | B | 4 | 1.15 | 0.29 | 2.38 | 3.07 | NA | 1 | - |
|  | 7 | B | 4 | 1.17 | 0.29 | 2.38 | 4.08 | NA | 0 | 0 |
|  | 8 | B | 4 | 1.17 | 0.29 | 2.38 | 2.18 | NA | 0 | 0 |
|  | 9 | B | 4 | 1.18 | 0.3 | 2.38 | 3.65 | NA | 1 | 94 |
|  | 10 | B | 4 | 1.14 | 0.28 | 2.39 | 1.98 | + | 1 | 88 |
| SK-ST41 | 1 | B | 4 | 1.14 | 0.29 | 2.39 | 1.91 | + | 1 | 80 |
|  | 2 | B | 4 | 1.15 | 0.29 | 2.39 | 1.81 | + | 1 | 92 |
|  | 3 | B | 4 | 1.15 | 0.29 | 2.39 | 2.4 | + | 1 | 80 |
|  | 4 | B | 4 | 1.15 | 0.29 | 2.4 | 2.42 | NA | 1 | 79 |
|  | 5 | B | 4 | 1.16 | 0.29 | 2.4 | 3.36 | + | 1 | 77 |
|  | 6 | B | 4 | 1.16 | 0.29 | 2.4 | 2.37 | + | 0 | 0 |
|  | 7 | B | 4 | 1.17 | 0.29 | 2.4 | 2.52 | + | 1 | 79 |
|  | 8 | B | 4 | 1.16 | 0.29 | 2.4 | 3.14 | + | 1 | 62 |
|  | 9 | B | 4 | 1.17 | 0.29 | 2.41 | 3.87 | NA | 1 | 59 |
|  | 10 | B | 4 | 1.15 | 0.29 | 2.41 | 2.63 | NA | 1 | 96 |
| SK-ST42 | 1 | B | 4 | 1.14 | 0.29 | 2.41 | 3.65 | NA | 0 | 0 |
|  | 2 | B | 4 | 1.15 | 0.29 | 2.42 | 3.04 | + | 0 | 80 |
|  | 3 | B | 4 | 1.19 | 0.3 | 2.42 | 3.78 | + | 1 | 62 |
|  | 4 | B | 4 | 1.11 | 0.28 | 2.42 | 1.8 | NA | 0 | 0 |
|  | 5 | B | 4 | 1.13 | 0.28 | 2.42 | 2.78 | + | 1 | 92 |
|  | 6 | B | 4 | 1.14 | 0.29 | 2.43 | 1.76 | + | 1 | 93 |
|  | 7 | B | 4 | 1.14 | 0.28 | 2.43 | 3.01 | + | 1 | 59 |
|  | 8 | B | 4 | 1.14 | 0.29 | 2.43 | 3.35 | + | 1 | 96 |
|  | 9 | B | 4 | 1.17 | 0.29 | 2.43 | 3.87 | + | 0 | 0 |
|  | 10 | B | 4 | 1.19 | 0.3 | 2.44 | 1.76 | NA | 0 | 0 |
| SK-ST43 | 1 | B | 4 | 1.15 | 0.29 | 2.44 | 1.84 | + | 1 | - |
|  | 2 | B | 4 | 1.18 | 0.3 | 2.45 | 2.77 | + | 1 | 94 |
|  | 3 | B | 4 | 1.16 | 0.29 | 2.45 | 1.57 | + | 1 | 83 |
|  | 4 | B | 4 | 1.16 | 0.29 | 2.45 | 2.64 | NA | 1 | 81 |
|  | 5 | B | 2 | 0.63 | 0.31 | 2.45 | 2.67 | + | 1 | 72 |
|  | 6 | B | 4 | 1.15 | 0.29 | 2.45 | 2.97 | + | 1 | 68 |
|  | 7 | B | 4 | 1.19 | 0.3 | 2.45 | 1.74 | + | 0 | 0 |
|  | 8 | B | 4 | 1.14 | 0.29 | 2.45 | 2.57 | + | 1 | 80 |
|  | 9 | B | 4 | 1.17 | 0.29 | 2.45 | 2.53 | + | 0 | 0 |
|  | 10 | B | 4 | 1.14 | 0.29 | 2.46 | 1.96 | + | 1 | 91 |
| SK-ST44 | 1 | B | 4 | 1.13 | 0.28 | 2.46 | 2.26 | + | 1 | 83 |
|  | 2 | B | 4 | 1.14 | 0.28 | 2.46 | 2.61 | NA | 1 | 92 |
|  | 3 | B | 4 | 1.13 | 0.28 | 2.46 | 2.56 | NA | 1 | 49 |
|  | 4 | B | 4 | 1.13 | 0.28 | 2.46 | 2.95 | NA | 1 | 92 |
|  | 5 | B | 4 | 1.15 | 0.29 | 2.47 | 2.69 | + | 1! | - |
|  | 6 | B | 4 | 1.14 | 0.28 | 2.47 | 2.35 | NA | 0 | 0 |
|  | 7 | B | 4 | 1.14 | 0.29 | 2.47 | 2.78 | NA | 1 | 91 |
|  | 8 | B | 4 | 1.17 | 0.29 | 2.48 | 4.22 | NA | 1 | 79 |
|  | 9 | B | 4 | 1.16 | 0.29 | 2.48 | 2.45 | + | 1 | 94 |
|  | 10 | B | 4 | 1.17 | 0.29 | 2.48 | 4.12 | + | 0 | 80 |
| SK-ST45 | 1 | B | 2 | 0.61 | 0.3 | 2.48 | 2.61 | NA | 1 | 73 |
|  | 2 | B | 2 | 0.62 | 0.31 | 2.48 | 3.45 | NA | 1 | 73 |
|  | 3 | B | 2 | 0.61 | 0.31 | 2.48 | 2.23 | NA | 1 | 73 |
|  | 4 | B | 2 | 0.6 | 0.3 | 2.49 | 3.91 | NA | 1 | 74 |
|  | 5 | B | 2 | 0.63 | 0.31 | 2.49 | 3.21 | NA | 1 | 74 |
|  | 6 | B | 2 | 0.63 | 0.31 | 2.49 | 2.99 | NA | 1 | 75 |
|  | 7 | B | 2 | 0.61 | 0.3 | 2.5 | 3 | NA | 1 | 75 |
|  | 8 | B | 2 | 0.63 | 0.32 | 2.5 | 3.85 | NA | 1 | 75 |
|  | 9 | B | 2 | 0.62 | 0.31 | 2.5 | 3 | NA | 1 | 75 |
|  | 10 | B | 2 | 0.61 | 0.31 | 2.5 | 3.08 | NA | 1 | 75 |
| SK-ST46 | 1 | B | 4 | 1.14 | 0.29 | 2.51 | 2.83 | NA | 1 | 90 |
|  | 2 | B | 4 | 1.17 | 0.29 | 2.51 | 2.75 | NA | 1 | 92 |
|  | 3 | B | 4 | 1.12 | 0.28 | 2.51 | 3.6 | NA | 1 | 77 |
|  | 4 | B | 4 | 1.18 | 0.3 | 2.51 | 3.25 | NA | 1 | 92 |
|  | 5 | B | 4 | 1.17 | 0.29 | 2.51 | 2.4 | NA | 1 | 89 |
|  | 6 | B | 4 | 1.15 | 0.29 | 2.51 | 1.88 | NA | 1 | 88 |
|  | 7 | B | 4 | 1.19 | 0.3 | 2.51 | 1.87 | NA | 1 | 64 |
|  | 8 | B | 4 | 1.17 | 0.29 | 2.52 | 1.74 | NA | 1 | 82 |
|  | 9 | B | 4 | 1.13 | 0.28 | 2.52 | 2.92 | NA | 1 | 81 |
|  | 10 | B | 4 | 1.15 | 0.29 | 2.52 | 3.2 | NA | 1 | 88 |
| SK-ST47 | 1 | B | 4 | 1.19 | 0.3 | 2.52 | 2.73 | NA | 1 | 91 |
|  | 2 | B | 4 | 1.15 | 0.29 | 2.52 | 3.2 | NA | 1 | 95 |
|  | 3 | B | 4 | 1.17 | 0.29 | 2.53 | 3.3 | NA | 1 | 72 |
|  | 4 | B | 4 | 1.19 | 0.3 | 2.53 | 4.03 | NA | 1 | 85 |
|  | 5 | B | 4 | 1.18 | 0.29 | 2.53 | 1.68 | NA | 1 | 90 |
|  | 6 | B | 4 | 1.16 | 0.29 | 2.54 | 3.25 | NA | 1 | 63 |
|  | 7 | B | 4 | 1.16 | 0.29 | 2.54 | 2.85 | NA | 1 | 82 |
|  | 8 | B | 4 | 1.18 | 0.29 | 2.54 | 3.2 | NA | 1 | 94 |
|  | 9 | B | 4 | 1.16 | 0.29 | 2.54 | 3.12 | NA | 1 | 98 |
|  | 10 | B | 4 | 1.17 | 0.29 | 2.55 | 1.86 | NA | 1 | 58 |
| SK-ST48 | 1 | B | 4 | 1.17 | 0.29 | 2.55 | 2.89 | NA | 0 | 0 |
|  | 2 | B | 4 | 1.18 | 0.29 | 2.55 | 2.9 | NA | 1 | 91 |
|  | 3 | B | 4 | 1.16 | 0.29 | 2.55 | 2.22 | + | 1 | 61 |
|  | 4 | B | 4 | 1.15 | 0.29 | 2.55 | 1.72 | + | 1 | - |
|  | 5 | B | 4 | 1.14 | 0.28 | 2.55 | 1.32 | + | 1 | 92 |
|  | 6 | B | 4 | 1.16 | 0.29 | 2.55 | 1.55 | + | 1 | 89 |
|  | 7 | B | 4 | 1.18 | 0.29 | 2.55 | 1.9 | + | 1 | 98 |
|  | 8 | B | 4 | 1.13 | 0.28 | 2.55 | 1.54 | + | 1 | 80 |
|  | 9 | B | 4 | 1.15 | 0.29 | 2.56 | 1.51 | + | 1 | - |
|  | 10 | B | 4 | 1.14 | 0.29 | 2.56 | 2.67 | + | 1 | 89 |
| SK-ST49 | 1 | B | 4 | 1.13 | 0.28 | 2.56 | 2.56 | + | 0 | 0 |
|  | 2 | B | 4 | 1.14 | 0.29 | 2.56 | 2.85 | + | 1 | 73 |
|  | 3 | B | 4 | 1.16 | 0.29 | 2.56 | 1.93 | + | 1 | 92 |
|  | 4 | B | 4 | 1.12 | 0.28 | 2.56 | 1.82 | NA | 1 | 79 |
|  | 5 | B | 4 | 1.17 | 0.29 | 2.57 | 2.04 | + | 1 | 80 |
|  | 6 | B | 4 | 1.14 | 0.29 | 2.57 | 3.41 | + | 1 | 91 |
|  | 7 | B | 4 | 1.15 | 0.29 | 2.57 | 1.93 | + | 1 | 94 |
|  | 8 | B | 4 | 1.13 | 0.28 | 2.58 | 2.81 | + | 1 | 81 |
|  | 9 | B | 4 | 1.14 | 0.28 | 2.58 | 3.05 | + | 1 | 94 |
|  | 10 | B | 4 | 1.18 | 0.29 | 2.58 | 3.96 | + | 1 | 82 |
| SK-ST50 | 2 | B | 4 | 1.15 | 0.29 | 2.58 | 2.46 | + | 0 | 0 |
|  | 3 | B | 4 | 1.17 | 0.29 | 2.58 | 3.93 | + | 1 | 98 |
|  | 4 | B | 4 | 1.14 | 0.29 | 2.58 | 2.04 | + | 1 | - |
|  | 5 | B | 4 | 1.15 | 0.29 | 2.58 | 3.02 | + | 1 | 97 |
|  | 6 | B | 4 | 1.17 | 0.29 | 2.59 | 2.15 | NA | 1 | 98 |
|  | 7 | B | 4 | 1.17 | 0.29 | 2.59 | 2.04 | + | 1 | 68 |
|  | 8 | B | 4 | 1.2 | 0.3 | 2.59 | 4.15 | + | 1 | 80 |
|  | 9 | B | 4 | 1.17 | 0.29 | 2.59 | 2.83 | + | 1 | 89 |
|  | 10 | B | 4 | 1.14 | 0.28 | 2.59 | 2.19 | + | 1 | 83 |
| UA-ST1 | 1 | B | 4 | 1.11 | 0.28 | 2.6 | 3.04 | + | 1 | 81 |
|  | 2 | B | 4 | 1.16 | 0.29 | 2.6 | 2.85 | + | 1 | 61 |
|  | 3 | B | 4 | 1.14 | 0.29 | 2.6 | 3.25 | + | 0 | 0 |
|  | 4 | B | 4 | 1.16 | 0.29 | 2.6 | 3.25 | + | 1 | 88 |
|  | 5 | B | 4 | 1.16 | 0.29 | 2.61 | 2.08 | NA | 0 | 0 |
|  | 6 | B | 4 | 1.16 | 0.29 | 2.61 | 2.23 | + | 1 | 64 |
|  | 7 | B | 4 | 1.18 | 0.29 | 2.61 | 3.08 | + | 0 | 0 |
|  | 8 | B | 4 | 1.18 | 0.29 | 2.61 | 2.08 | + | 1 | 96 |
|  | 9 | B | 4 | 1.14 | 0.28 | 2.61 | 2.63 | + | 1 | 94 |
|  | 10 | B | 4 | 1.17 | 0.29 | 2.61 | 1.8 | NA | 1 | 83 |
| UA-ST2 | 1 | B | 4 | 1.1 | 0.37 | 2.61 | 1.7 | NA | 0 | 0 |
|  | 2 | G* | 4 | 1.08 | 0.36 | 2.62 | 1.54 | NA | 0 | 0 |
|  | 3 | B | 4 | 1.1 | 0.37 | 2.62 | 1.93 | NA | 0 | 0 |
|  | 4 | G* | 4 | 1.08 | 0.36 | 2.62 | 1.61 | NA | 0 | 0 |
|  | 5 | B | 4 | 1.12 | 0.28 | 2.63 | 3.02 | NA | 0 | 0 |
|  | 6 | B | 2 | 0.67 | 0.33 | 2.63 | 3.28 | + | 0 | 0 |
|  | 7 | B | 4 | 1.18 | 0.29 | 2.64 | 3.25 | NA | 0 | 0 |
|  | 8 | B | 4 | 1.18 | 0.3 | 2.65 | 3.25 | NA | 0 | 0 |
|  | 9 | B | 4 | 1.15 | 0.29 | 2.65 | 3.32 | NA | 1 | - |
|  | 10 | B | 4 | 1.15 | 0.29 | 2.65 | 3.9 | + | 1 | 77 |
| UA-ST3 | 1 | B | 2 | 0.62 | 0.31 | 2.65 | 1.93 | + | 0 | 0 |
|  | 2 | B | 2 | 0.62 | 0.31 | 2.65 | 2.42 | + | 0 | 0 |
|  | 3 | B | 2 | 0.63 | 0.31 | 2.65 | 3.25 | + | 0 | 0 |
|  | 4 | B | 2 | 0.63 | 0.31 | 2.65 | 3.2 | + | 1 | 76 |
|  | 5 | B | 2 | 0.67 | 0.33 | 2.66 | 3.06 | + | 0 | 0 |
|  | 6 | B | 2 | 0.62 | 0.31 | 2.66 | 1.9 | + | 0 | 0 |
|  | 7 | B | 2 | 0.62 | 0.31 | 2.66 | 2.67 | + | 0 | 0 |
|  | 8 | B | 2 | 0.67 | 0.33 | 2.66 | 2.56 | + | 0 | 0 |
|  | 9 | B | 2 | 0.62 | 0.31 | 2.66 | 1.99 | NA | 0 | 0 |
|  | 10 | B | 2 | 0.63 | 0.31 | 2.66 | 3.18 | + | 1 | 76 |
| UA-ST4 | 1 | B | 4 | 1.16 | 0.29 | 2.67 | 1.75 | NA | 1 | 91 |
|  | 2 | B | 2 | 0.62 | 0.31 | 2.67 | 3.47 | NA | 0 | 0 |
|  | 3 | B | 2 | 0.63 | 0.31 | 2.67 | 3.22 | + | 1 | 76 |
|  | 4 | B | 2 | 0.63 | 0.31 | 2.67 | 3.67 | + | 1! | 76 |
|  | 5 | B | 2 | 0.63 | 0.31 | 2.68 | 2.36 | NA | 1 | 76 |
|  | 6 | B | 2 | 0.63 | 0.31 | 2.68 | 1.97 | + | 0 | 0 |
|  | 7 | B | 2 | 0.63 | 0.31 | 2.68 | 2.3 | + | 0 | 0 |
|  | 8 | B | 2 | 0.62 | 0.31 | 2.68 | 2.2 | + | 1 | 76 |
|  | 9 | B | 2 | 0.62 | 0.31 | 2.68 | 2.14 | + | 1 | 77 |
|  | 10 | B | 2 | 0.63 | 0.31 | 2.68 | 3.06 | + | 1 | 78 |
| UA-ST5 | 1 | B | 4 | 1.19 | 0.3 | 2.68 | 3.02 | + | 1 | 79 |
|  | 2 | B | 4 | 1.17 | 0.29 | 2.68 | 3.02 | + | 1 | - |
|  | 3 | B | 4 | 1.17 | 0.29 | 2.69 | 2.17 | + | 0 | - |
|  | 4 | B | 4 | 1.2 | 0.3 | 2.69 | 2.02 | + | 1 | - |
|  | 5 | B | 4 | 1.18 | 0.3 | 2.69 | 1.98 | + | 1 | 92 |
|  | 6 | B | 4 | 1.16 | 0.29 | 2.69 | 3.31 | + | 0 | 0 |
|  | 7 | B | 2 | 0.64 | 0.32 | 2.69 | 2.09 | + | 0 | 0 |
|  | 8 | B | 4 | 1.19 | 0.3 | 2.69 | 1.66 | + | 1 | - |
|  | 9 | B | 4 | 1.17 | 0.29 | 2.69 | 1.82 | + | 1 | - |
|  | 10 | B | 4 | 1.17 | 0.29 | 2.69 | 1.87 | + | 1 | - |
| UA-ST6 | 1 | B | 4 | 1.15 | 0.29 | 2.69 | 1.57 | + | 1 | 91 |
|  | 2 | B | 4 | 1.18 | 0.29 | 2.69 | 3.2 | NA | 1 | 74 |
|  | 3 | B | 4 | 1.16 | 0.29 | 2.69 | 1.95 | + | 0 | 0 |
|  | 4 | B | 4 | 1.17 | 0.29 | 2.69 | 1.82 | + | 1 | 92 |
|  | 5 | B | 4 | 1.16 | 0.29 | 2.69 | 1.69 | + | 1 | 68 |
|  | 6 | B | 4 | 1.19 | 0.3 | 2.69 | 3.17 | + | 1 | 62 |
|  | 7 | B | 4 | 1.15 | 0.29 | 2.69 | 1.4 | + | 0 | 0 |
|  | 8 | B | 4 | 1.14 | 0.29 | 2.69 | 1.59 | + | 1 | 64 |
|  | 9 | B | 4 | 1.15 | 0.29 | 2.7 | 1.86 | + | 1 | 77 |
|  | 10 | B | 4 | 1.17 | 0.29 | 2.7 | 1.77 | + | 1 | 64 |
| UA-ST7 | 1 | B | 4 | 1.15 | 0.29 | 2.7 | 1.88 | + | 1 | 89 |
|  | 2 | B | 4 | 1.14 | 0.28 | 2.7 | 1.5 | + | 1 | 91 |
|  | 3 | B | 4 | 1.16 | 0.29 | 2.7 | 1.71 | + | 0 | 0 |
|  | 4 | B | 4 | 1.16 | 0.29 | 2.7 | 2.44 | + | 0 | 0 |
|  | 5 | B | 4 | 1.14 | 0.29 | 2.7 | 1.69 | + | 0 | 0 |
|  | 6 | B | 4 | 1.16 | 0.29 | 2.71 | 1.86 | + | 1 | 95 |
|  | 7 | B | 4 | 1.17 | 0.29 | 2.71 | 1.7 | + | 0 | 0 |
|  | 8 | B | 4 | 1.15 | 0.29 | 2.71 | 1.67 | + | 1 | 84 |
|  | 9 | B | 4 | 1.16 | 0.29 | 2.71 | 1.9 | + | 0 | 0 |
|  | 10 | B | 4 | 1.17 | 0.29 | 2.71 | 2.69 | + | 1 | 95 |
| UA-ST8 | 1 | B | 4 | 1.14 | 0.28 | 2.72 | 2.13 | + | 1 | 86 |
|  | 2 | B | 4 | 1.12 | 0.28 | 2.72 | 1.97 | + | 1 | 80 |
|  | 3 | B | 4 | 1.17 | 0.29 | 2.72 | 3.5 | + | 1 | 82 |
|  | 4 | B | 4 | 1.14 | 0.29 | 2.72 | 3.41 | + | 1 | 84 |
|  | 5 | B | 4 | 1.1 | 0.28 | 2.73 | 1.84 | + | 1! | - |
|  | 6 | B | 4 | 1.16 | 0.29 | 2.73 | 2 | + | 1 | 76 |
|  | 7 | B | 4 | 1.17 | 0.29 | 2.74 | 3.12 | + | 0 | 0 |
|  | 8 | B | 4 | 1.16 | 0.29 | 2.74 | 3.12 | + | 0 | 0 |
|  | 9 | B | 4 | 1.15 | 0.29 | 2.74 | 3.05 | + | 1 | 91 |
|  | 10 | B | 4 | 1.13 | 0.28 | 2.74 | 1.64 | + | 1 | 88 |
| UA-ST9 | 1 | B | 4 | 1.14 | 0.28 | 2.74 | 2.78 | + | 0 | 0 |
|  | 2 | B | 4 | 1.12 | 0.28 | 2.74 | 2.97 | + | 1 | 81 |
|  | 3 | B | 4 | 1.15 | 0.29 | 2.74 | 2.63 | + | 0 | 0 |
|  | 4 | B | 4 | 1.15 | 0.29 | 2.75 | 2.47 | + | 1 | 82 |
|  | 5 | B | 4 | 1.13 | 0.28 | 2.75 | 2.05 | + | 1 | 88 |
|  | 6 | B | 4 | 1.15 | 0.29 | 2.75 | 3.12 | + | 1 | 80 |
|  | 7 | B | 4 | 1.14 | 0.29 | 2.76 | 3.22 | + | 1 | 79 |
|  | 8 | B | 4 | 0.61 | 0.31 | 2.76 | 3.25 | + | 1 | 95 |
|  | 9 | B | 2 | 1.14 | 0.29 | 2.76 | 2.04 | NA | 0 | - |
| UA-ST10 | 1 | B | 4 | 1.16 | 0.29 | 2.76 | 3.5 | + | 1 | 91 |
|  | 2 | B | 4 | 1.14 | 0.28 | 2.77 | 1.96 | + | 1 | 92 |
|  | 3 | B | 4 | 1.12 | 0.28 | 2.77 | 1.68 | + | 1 | 90 |
|  | 4 | B | 4 | 1.14 | 0.29 | 2.77 | 2.85 | NA | 1 | 81 |
|  | 5 | B | 4 | 1.13 | 0.28 | 2.78 | 2.6 | + | 1 | 89 |
|  | 6 | B | 4 | 1.12 | 0.28 | 2.78 | 2.81 | + | 1 | 82 |
|  | 7 | B | 4 | 1.14 | 0.28 | 2.79 | 2.55 | + | 0 | 0 |
|  | 8 | B | 4 | 1.12 | 0.28 | 2.79 | 2.59 | + | 1 | 86 |
|  | 9 | B | 4 | 1.13 | 0.28 | 2.79 | 1.31 | + | 1 | 94 |
|  | 10 | B | 4 | 1.14 | 0.28 | 2.79 | 3.5 | + | 0 | 0 |
| UA-ST11 | 1 | B | 4 | 1.15 | 0.29 | 2.79 | 2.87 | + | 1 | 89 |
|  | 2 | B | 4 | 1.1 | 0.28 | 2.79 | 2.74 | + | 1 | 92 |
|  | 3 | B | 4 | 1.15 | 0.29 | 2.79 | 2.74 | + | 1 | 86 |
|  | 4 | B | 4 | 1.13 | 0.28 | 2.79 | 2.48 | NA | 0 | 0 |
|  | 5 | B | 4 | 1.14 | 0.28 | 2.8 | 2.62 | + | 1 | 42 |
|  | 6 | B | 4 | 1.17 | 0.29 | 2.8 | 3.15 | + | 1 | 91 |
|  | 7 | B | 4 | 1.14 | 0.29 | 2.8 | 3.65 | + | 1 | - |
|  | 8 | B | 4 | 1.15 | 0.29 | 2.8 | 2.38 | + | 0 | 0 |
|  | 9 | B | 4 | 1.15 | 0.29 | 2.8 | 2.85 | + | 0 | 0 |
|  | 10 | B | 4 | 1.15 | 0.29 | 2.81 | 2.85 | + | 0 | 0 |
| UA-ST12 | 1 | B | 4 | 1.17 | 0.29 | 2.81 | 2.52 | + | 1 | 89 |
|  | 2 | B | 4 | 1.16 | 0.29 | 2.81 | 2.51 | + | 1 | 83 |
|  | 3 | B | 4 | 1.17 | 0.29 | 2.82 | 2.53 | + | 0 | - |
|  | 4 | B | 4 | 1.17 | 0.29 | 2.82 | 2.45 | + | 1 | 79 |
|  | 5 | B | 4 | 1.15 | 0.29 | 2.82 | 2.71 | + | 0 | 0 |
|  | 6 | B | 4 | 1.16 | 0.29 | 2.82 | 2.19 | + | 1 | 57 |
|  | 7 | B | 4 | 1.14 | 0.29 | 2.83 | 2.76 | + | 1 | 80 |
|  | 8 | B | 4 | 1.16 | 0.29 | 2.83 | 2.34 | + | 0 | 0 |
|  | 9 | B | 4 | 1.16 | 0.29 | 2.83 | 1.85 | + | 1 | 65 |
|  | 10 | B | 4 | 1.14 | 0.29 | 2.83 | 2.2 | + | 1 | 79 |
| RO-ST1 | 1 | B | 2 | 0.62 | 0.31 | 2.83 | 3.8 | + | 1 | 78 |
|  | 2 | B | 4 | 1.16 | 0.29 | 2.83 | 2.44 | + | 1 | 80 |
|  | 3 | B | 2 | 0.61 | 0.3 | 2.83 | 3.13 | + | 1 | 78 |
|  | 4 | B | 2 | 0.62 | 0.31 | 2.83 | 3.76 | + | 1 | 78 |
|  | 5 | B | 2 | 0.53 | 0.27 | 2.83 | 3.81 | + | 1 | 78 |
|  | 6 | B | 4 | 1.18 | 0.3 | 2.84 | 3.4 | + | 1 | 76 |
|  | 7 | B | 2 | 0.64 | 0.32 | 2.84 | 2 | + | 1 | 79 |
|  | 8 | B | 2 | 0.55 | 0.27 | 2.84 | 3.61 | + | 1 | 79 |
|  | 9 | B | 2 | 0.65 | 0.33 | 2.84 | 2.12 | + | 1 | 79 |
| RO-ST2 | 1 | B | 2 | 0.61 | 0.3 | 2.84 | 2.5 | + | 1 | 79 |
|  | 2 | B | 2 | 0.62 | 0.31 | 2.84 | 3.7 | + | 1 | 79 |
|  | 3 | B | 2 | 0.61 | 0.3 | 2.84 | 2.77 | + | 1 | 79 |
|  | 4 | B | 2 | 0.61 | 0.31 | 2.84 | 3.6 | + | 1 | 80 |
|  | 5 | B | 2 | 0.61 | 0.31 | 2.85 | 3.21 | + | 1 | 80 |
|  | 8 | B | 2 | 0.63 | 0.31 | 2.85 | 2.76 | + | 1 | 80 |
|  | 9 | B | 2 | 0.63 | 0.32 | 2.85 | 2.32 | + | 1 | 80 |
|  | 10 | B | 2 | 0.65 | 0.32 | 2.85 | 2.67 | + | 1 | 80 |
| RO-ST3 | 1 | B | 2 | 0.62 | 0.31 | 2.85 | 2.93 | + | 1 | 80 |
|  | 2 | B | 2 | 0.61 | 0.31 | 2.86 | 2.46 | + | 0 | 0 |
|  | 3 | B | 2 | 0.62 | 0.31 | 2.86 | 4.23 | + | 1 | 80 |
|  | 4 | B | 2 | 0.63 | 0.31 | 2.86 | 3.76 | + | 1 | 80 |
|  | 5 | B | 2 | 0.63 | 0.32 | 2.86 | 3.18 | + | 0 | 0 |
|  | 6 | B | 2 | 0.62 | 0.31 | 2.86 | 3.42 | + | 1 | 80 |
|  | 7 | B | 2 | 0.63 | 0.31 | 2.86 | 2.36 | + | 0 | 0 |
|  | 8 | B | 2 | 0.66 | 0.33 | 2.86 | 3.15 | + | 1 | 81 |
|  | 9 | B | 2 | 0.65 | 0.32 | 2.86 | 2.79 | + | 1 | 81 |
|  | 10 | B | 2 | 0.64 | 0.32 | 2.86 | 3.48 | + | 0 | 0 |
| RO-ST4 | 1 | B | 2 | 0.63 | 0.31 | 2.86 | 4 | + | 0 | 0 |
|  | 2 | B | 2 | 0.64 | 0.32 | 2.86 | 2.64 | + | 1 | 81 |
|  | 3 | B | 2 | 0.63 | 0.31 | 2.86 | 2.47 | + | 0 | 0 |
|  | 4 | B | 2 | 0.64 | 0.32 | 2.87 | 4.02 | + | 1 | 82 |
|  | 5 | B | 2 | 0.65 | 0.32 | 2.87 | 2.05 | + | 1 | 82 |
|  | 6 | B | 2 | 0.65 | 0.32 | 2.87 | 2.96 | + | 1 | 82 |
|  | 7 | B | 2 | 0.65 | 0.32 | 2.87 | 3.38 | + | 1 | 82 |
|  | 8 | B | 2 | 0.66 | 0.33 | 2.87 | 2.94 | + | 1 | 82 |
| RO-ST5 | 1 | B | 4 | 1.13 | 0.28 | 2.87 | 1.62 | + | 1 | - |
|  | 2 | B | 4 | 1.11 | 0.28 | 2.87 | 1.75 | + | 1! | - |
|  | 3 | B | 4 | 1.13 | 0.28 | 2.88 | 2.03 | NA | 0 | 0 |
|  | 4 | B | 4 | 1.12 | 0.28 | 2.88 | 2.33 | + | 1 | - |
|  | 5 | B | 4 | 1.15 | 0.29 | 2.88 | 2.31 | + | 1 | - |
|  | 6 | B | 2 | 0.63 | 0.32 | 2.88 | 4.05 | + | 1! | 82 |
|  | 8 | B | 4 | 1.18 | 0.29 | 2.88 | 2.43 | + | 1 | 92 |
|  | 10 | B | 2 | 0.54 | 0.27 | 2.89 | 3.51 | NA | 1 | 82 |
| RO-ST6 | 1 | B | 2 | 0.63 | 0.32 | 2.89 | 2.27 | + | 1 | 82 |
|  | 2 | B | 2 | 0.65 | 0.33 | 2.89 | 3.11 | + | 0 | 0 |
|  | 3 | B | 2 | 0.63 | 0.31 | 2.89 | 1.65 | + | 0 | 0 |
|  | 4 | B | 2 | 0.63 | 0.31 | 2.89 | 3.65 | + | 0 | 0 |
|  | 5 | B | 2 | 0.64 | 0.32 | 2.9 | 2.03 | + | 1 | 82 |
|  | 6 | B | 2 | 0.64 | 0.32 | 2.9 | 3.33 | + | 1 | 82 |
|  | 7 | B | 2 | 0.63 | 0.32 | 2.9 | 3.05 | + | 1 | 83 |
|  | 8 | B | 2 | 0.61 | 0.31 | 2.9 | 1.64 | + | 0 | 0 |
|  | 9 | B | 2 | 0.61 | 0.31 | 2.91 | 3.65 | + | 0 | 0 |
| RO-ST7 | 1 | B | 2 | 0.64 | 0.32 | 2.92 | 2.34 | + | 1! | 83 |
|  | 2 | B | 2 | 0.63 | 0.32 | 2.92 | 1.85 | + | 0 | 0 |
|  | 3 | B | 2 | 0.61 | 0.31 | 2.92 | 3.63 | + | 1 | 83 |
|  | 4 | B | 2 | 0.62 | 0.31 | 2.93 | 3.25 | + | 1 | 83 |
|  | 5 | B | 2 | 0.62 | 0.31 | 2.93 | 3.83 | + | 1! | 84 |
|  | 6 | B | 2 | 0.62 | 0.31 | 2.94 | 3.54 | + | 1 | 84 |
|  | 7 | B | 2 | 0.65 | 0.32 | 2.94 | 1.89 | + | 1 | 84 |
|  | 8 | B | 2 | 0.65 | 0.32 | 2.94 | 1.76 | + | 1 | 84 |
|  | 9 | B | 2 | 0.64 | 0.32 | 2.94 | 2.14 | + | 1 | 85 |
|  | 10 | B | 2 | 0.64 | 0.32 | 2.94 | 3.27 | + | 1 | 85 |
| RO-ST8 | 1 | B | 2 | 0.62 | 0.31 | 2.95 | 3.08 | + | 1! | 85 |
|  | 2 | B | 2 | 0.62 | 0.31 | 2.95 | 3.08 | + | 1 | 85 |
|  | 3 | B | 2 | 0.62 | 0.31 | 2.95 | 3.08 | NA | 1 | 85 |
|  | 4 | B | 2 | 0.62 | 0.31 | 2.95 | 2.52 | + | 0 | 0 |
|  | 5 | B | 2 | 0.62 | 0.31 | 2.95 | 2.52 | NA | 1 | 86 |
|  | 6 | B | 2 | 0.62 | 0.31 | 2.95 | 2.52 | + | 1 | 86 |
|  | 7 | B | 2 | 0.62 | 0.31 | 2.95 | 3.56 | + | 1 | 87 |
|  | 8 | B | 2 | 0.6 | 0.3 | 2.95 | 1.01 | + | 0 | 0 |
|  | 9 | B | 4 | 1.12 | 0.28 | 2.95 | 1.72 | + | 0 | 0 |
|  | 10 | B | 2 | 0.64 | 0.32 | 2.96 | 2.8 | + | 1 | 87 |
| RO-ST9 | 1 | B | 4 | 1.13 | 0.29 | 2.96 | 3.12 | + | 1 | 85 |
|  | 2 | B | 4 | 1.15 | 0.29 | 2.96 | 2.61 | NA | 0 | 0 |
|  | 3 | B | 4 | 1.15 | 0.29 | 2.96 | 3.28 | + | 1 | 62 |
|  | 4 | B | 4 | 1.17 | 0.29 | 2.96 | 3.54 | NA | 1! | 91 |
|  | 5 | B | 4 | 1.12 | 0.29 | 2.96 | 2.74 | NA | 1 | - |
|  | 7 | B | 4 | 1.17 | 0.29 | 2.96 | 1.94 | + | 1 | 94 |
|  | 9 | B | 4 | 1.14 | 0.29 | 2.96 | 2.23 | + | 1 | 90 |
|  | 10 | B | 4 | 1.14 | 0.28 | 2.96 | 2.35 | + | 1 | 83 |
| RO-ST10 | 1 | B | 2 | 0.65 | 0.32 | 2.96 | 3.03 | + | 0 | 0 |
|  | 2 | B | 2 | 0.65 | 0.32 | 2.96 | 3.26 | + | 0 | 0 |
|  | 3 | B | 2 | 0.64 | 0.32 | 2.97 | 3.98 | + | 0 | - |
|  | 4 | B | 2 | 0.63 | 0.31 | 2.97 | 3.54 | + | 1 | 87 |
|  | 5 | B | 2 | 0.65 | 0.32 | 2.97 | 2.13 | + | 0 | 0 |
|  | 6 | B | 2 | 0.65 | 0.32 | 2.97 | 2.09 | + | 0 | 0 |
|  | 7 | B | 2 | 0.62 | 0.31 | 2.98 | 2.62 | + | 1 | 87 |
|  | 8 | B | 2 | 0.64 | 0.32 | 2.98 | 3.76 | + | 0 | 0 |
|  | 9 | B | 2 | 0.65 | 0.33 | 2.98 | 3.27 | + | 0 | 0 |
| RO-ST11 | 1 | B | 4 | 1.15 | 0.29 | 2.98 | 3.06 | + | 1 | 72 |
|  | 2 | B | 4 | 1.16 | 0.29 | 2.98 | 2.88 | + | 0 | 0 |
|  | 3 | B | 4 | 1.16 | 0.29 | 2.98 | 2.98 | + | 1 | 89 |
|  | 4 | B | 4 | 1.16 | 0.29 | 2.99 | 3.25 | + | 0 | 0 |
|  | 5 | B | 4 | 1.17 | 0.29 | 2.99 | 3.52 | + | 1 | 64 |
|  | 6 | B | 4 | 1.16 | 0.29 | 2.99 | 3.69 | + | 1 | 85 |
|  | 7 | B | 4 | 1.17 | 0.29 | 2.99 | 2.43 | NA | 1! | - |
|  | 8 | B | 4 | 1.15 | 0.29 | 2.99 | 2.92 | + | 0 | 0 |
|  | 9 | B | 4 | 1.16 | 0.29 | 2.99 | 3.24 | + | 1 | 92 |
|  | 10 | B | 4 | 1.15 | 0.29 | 2.99 | 3.28 | + | 0 | 0 |
| RO-ST12 | 1 | B | 2 | 0.62 | 0.31 | 2.99 | 3.01 | + | 1 | 88 |
|  | 2 | B | 4 | 1.2 | 0.3 | 3 | 3.2 | + | 1 | 81 |
|  | 3 | B | 4 | 1.19 | 0.3 | 3 | 3.46 | + | 1 | 91 |
|  | 4 | B | 2 | 0.64 | 0.32 | 3 | 3.56 | + | 1 | 88 |
|  | 5 | B | 4 | 1.16 | 0.29 | 3 | 2.53 | + | 1 | 85 |
|  | 6 | B | 2 | 0.64 | 0.32 | 3.01 | 3.34 | + | 0 | 0 |
|  | 7 | B | 2 | 0.62 | 0.31 | 3.01 | 3.57 | + | 1 | 88 |
|  | 8 | B | 4 | 1.18 | 0.3 | 3.01 | 3.76 | + | 1 | 88 |
|  | 9 | B | 2 | 0.64 | 0.32 | 3.02 | 4.23 | + | 0 | 0 |
|  | 10 | B | 2 | 0.63 | 0.31 | 3.02 | 3.51 | + | 0 | 0 |
| RO-ST13 | 1 | B | 2 | 0.62 | 0.31 | 3.02 | 3.53 | NA | 1 | 88 |
|  | 2 | B | 2 | 0.62 | 0.31 | 3.02 | 3.62 | NA | 1 | 88 |
|  | 3 | B | 2 | 0.62 | 0.31 | 3.03 | 2.68 | + | 1 | 88 |
|  | 4 | B | 2 | 0.63 | 0.31 | 3.03 | 3.54 | NA | 1 | 88 |
|  | 5 | B | 2 | 0.62 | 0.31 | 3.03 | 3.39 | + | 0 | 0 |
|  | 6 | B | 2 | 0.63 | 0.31 | 3.03 | 3.61 | + | 0 | 0 |
|  | 7 | B | 2 | 0.61 | 0.31 | 3.03 | 3.65 | + | 1 | 88 |
|  | 8 | B | 2 | 0.62 | 0.31 | 3.04 | 3.65 | + | 0 | 0 |
|  | 9 | B | 2 | 0.62 | 0.31 | 3.04 | 3.42 | + | 1 | 88 |
|  | 10 | B | 2 | 0.61 | 0.31 | 3.04 | 3.56 | + | 0 | 0 |
| RO-ST14 | 1 | B | 2 | 0.61 | 0.3 | 3.04 | 3.19 | + | 1 | 88 |
|  | 2 | B | 2 | 0.63 | 0.31 | 3.05 | 4.02 | + | 1 | 88 |
|  | 3 | B | 2 | 0.62 | 0.31 | 3.05 | 3.24 | + | 1 | 88 |
|  | 4 | B | 2 | 0.64 | 0.32 | 3.05 | 4.03 | + | 1 | 88 |
|  | 5 | B | 2 | 0.61 | 0.3 | 3.05 | 3.86 | + | 1 | 88 |
|  | 6 | B | 2 | 0.61 | 0.3 | 3.05 | 4.05 | + | 1 | 88 |
|  | 7 | B | 2 | 0.61 | 0.3 | 3.05 | 3.75 | + | 1 | 88 |
|  | 8 | B | 2 | 0.62 | 0.31 | 3.05 | 3.62 | + | 0 | 0 |
|  | 9 | B | 2 | 0.6 | 0.3 | 3.06 | 4.02 | NA | 1 | 89 |
|  | 10 | B | 2 | 0.63 | 0.31 | 3.06 | 3.09 | + | 0 | 0 |
| RO-ST15 | 1 | B | 2 | 0.63 | 0.32 | 3.06 | 4.02 | + | 1 | 89 |
|  | 2 | B | 2 | 0.61 | 0.3 | 3.06 | 3.35 | NA | 1 | 89 |
|  | 3 | B | 2 | 0.61 | 0.31 | 3.06 | 3.34 | + | 1 | 89 |
|  | 4 | B | 2 | 0.63 | 0.32 | 3.06 | 4.02 | + | 1 | 89 |
|  | 5 | B | 2 | 0.62 | 0.31 | 3.06 | 3.91 | + | 1 | 89 |
|  | 6 | B | 4 | 1.19 | 0.3 | 3.07 | 2.52 | + | 0 | 0 |
|  | 7 | B | 2 | 0.63 | 0.31 | 3.07 | 4.06 | + | 0 | 0 |
|  | 8 | B | 2 | 0.61 | 0.3 | 3.07 | 4.05 | + | 0 | 0 |
|  | 9 | B | 2 | 0.62 | 0.31 | 3.07 | 4.13 | + | 0 | 0 |
|  | 10 | B | 2 | 0.61 | 0.3 | 3.08 | 3.68 | NA | 0 | 0 |
| RO-ST16 | 1 | B | 4 | 1.14 | 0.28 | 3.08 | 2.58 | NA | 0 | 0 |
|  | 2 | B | 4 | 1.16 | 0.29 | 3.08 | 3.52 | NA | 0 | 0 |
|  | 3 | B | 4 | 1.16 | 0.29 | 3.08 | 3.52 | + | 0 | 0 |
|  | 4 | B | 4 | 1.13 | 0.28 | 3.09 | 3.3 | + | 1 | 87 |
|  | 5 | B | 4 | 1.12 | 0.28 | 3.09 | 2.65 | NA | 1 | 94 |
|  | 6 | B | 4 | 1.12 | 0.28 | 3.09 | 2.31 | NA | 1 | 88 |
|  | 7 | B | 4 | 1.12 | 0.28 | 3.09 | 2.5 | NA | 1 | 78 |
|  | 8 | B | 4 | 1.14 | 0.28 | 3.09 | 2.5 | + | 1 | 83 |
|  | 9 | B | 4 | 1.12 | 0.28 | 3.09 | 2.35 | NA | 1 | 91 |
|  | 10 | B | 4 | 1.13 | 0.28 | 3.09 | 2.77 | + | 1 | 90 |
| RO-ST17 | 1 | B | 2 | 0.63 | 0.32 | 3.09 | 3.46 | + | 1 | 89 |
|  | 2 | B | 2 | 0.62 | 0.32 | 3.1 | 3.33 | + | 1 | 89 |
|  | 3 | B | 2 | 0.61 | 0.32 | 3.1 | 2.65 | + | 1 | 90 |
|  | 4 | B | 2 | 0.62 | 0.31 | 3.11 | 3.15 | + | 1 | 90 |
|  | 5 | B | 2 | 0.62 | 0.31 | 3.12 | 3.09 | + | 0 | 0 |
|  | 6 | B | 2 | 0.61 | 0.31 | 3.12 | 2.4 | + | 1 | 90 |
|  | 7 | B | 2 | 0.62 | 0.31 | 3.12 | 3.2 | + | 0 | 0 |
|  | 8 | B | 2 | 0.61 | 0.31 | 3.12 | 2.25 | + | 1 | 90 |
|  | 9 | B | 2 | 0.61 | 0.31 | 3.12 | 2.27 | + | 1 | 90 |
|  | 10 | B | 2 | 0.62 | 0.31 | 3.12 | 2.97 | + | 1 | 90 |
| RO-ST18 | 1 | B | 2 | 0.64 | 0.32 | 3.12 | 2.54 | + | 1 | 90 |
|  | 2 | B | 2 | 0.65 | 0.32 | 3.12 | 2.19 | + | 1 | 90 |
|  | 3 | B | 2 | 0.63 | 0.32 | 3.13 | 2.73 | + | 1 | 90 |
|  | 4 | B | 2 | 0.61 | 0.31 | 3.13 | 3.25 | NA | 0 | 0 |
|  | 5 | B | 2 | 0.62 | 0.31 | 3.13 | 3.25 | NA | 1 | 91 |
|  | 6 | B | 2 | 0.61 | 0.31 | 3.13 | 2.76 | + | 1 | 91 |
|  | 7 | B | 2 | 0.63 | 0.31 | 3.13 | 3.65 | + | 1 | 91 |
|  | 8 | B | 2 | 0.63 | 0.31 | 3.14 | 3.23 | + | 1 | 91 |
|  | 9 | B | 2 | 0.62 | 0.31 | 3.14 | 3.1 | + | 1 | 91 |
|  | 10 | B | 2 | 0.62 | 0.31 | 3.14 | 3.25 | + | 0 | 0 |
| RO-ST19 | 1 | B | 4 | 1.1 | 0.37 | 3.14 | 2.54 | + | 0 | 0 |
|  | 2 | G | 4* | 1.08 | 0.36 | 3.14 | 2.36 | + | 1 | 59 |
|  | 3 | G | 4* | 1.06 | 0.35 | 3.15 | 1.31 | + | 0 | 0 |
|  | 4 | B | 4 | 1.1 | 0.37 | 3.15 | 1.71 | NA | 0 | 0 |
|  | 5 | G | 4* | 1.07 | 0.36 | 3.15 | 1.42 | + | 0 | 0 |
|  | 6 | G | 4* | 1.07 | 0.36 | 3.15 | 0.94 | NA | 1! | 58 |
|  | 7 | G | 4* | 1.07 | 0.36 | 3.15 | 1.26 | + | 1 | 92 |
|  | 8 | G | 4* | 1.07 | 0.36 | 3.15 | 1.35 | + | 0 | 0 |
|  | 9 | B | 4 | 1.09 | 0.36 | 3.15 | 1.76 | + | 0 | - |
|  | 10 | G | 4* | 1.08 | 0.36 | 3.16 | 1.62 | NA | 0 | 0 |
| RO-ST20 | 1 | B | 2 | 0.63 | 0.31 | 3.16 | 3.05 | + | 1 | 92 |
|  | 2 | B | 2 | 0.63 | 0.32 | 3.16 | 3.2 | + | 1 | 92 |
|  | 3 | B | 2 | 0.6 | 0.3 | 3.16 | 3 | + | 1 | 92 |
|  | 4 | B | 2 | 0.63 | 0.31 | 3.17 | 2.65 | NA | 1 | 92 |
|  | 5 | B | 4 | 1.17 | 0.29 | 3.17 | 3.25 | + | 0 | 0 |
|  | 6 | B | 2 | 0.62 | 0.31 | 3.17 | 2.65 | + | 1 | 92 |
|  | 7 | B | 2 | 0.63 | 0.31 | 3.17 | 2.36 | + | 1 | 92 |
|  | 8 | B | 2 | 0.62 | 0.31 | 3.18 | 2.78 | NA | 1 | 92 |
|  | 9 | B | 2 | 0.63 | 0.31 | 3.19 | 3.08 | + | 0 | 0 |
|  | 10 | B | 2 | 0.62 | 0.31 | 3.2 | 2.94 | NA | 1 | 92 |
| RO-ST21 | 1 | B | 2 | 0.63 | 0.31 | 3.2 | 3.25 | + | 1 | 93 |
|  | 2 | B | 2 | 0.62 | 0.31 | 3.2 | 2.91 | + | 1 | 93 |
|  |  | G | 4* | 1.43 | 0.34 | 3.2 | 2.19 | + | 1 | 93 |
|  | 4 | B | 2 | 0.61 | 0.31 | 3.21 | 3.38 | + | 1 | 93 |
|  | 5 | B | 2 | 0.63 | 0.31 | 3.21 | 3.25 | + | 0 | 0 |
|  | 6 | B | 2 | 0.63 | 0.32 | 3.21 | 3.63 | + | 0 | 0 |
|  | 7 | G | 4* | 1.44 | 0.35 | 3.22 | 3.65 | NA | 1 | 94 |
|  | 8 | G | 4* | 1.47 | 0.35 | 3.22 | 2.69 | NA | 1 | 72 |
|  | 9 | B | 2 | 0.64 | 0.32 | 3.22 | 3.02 | + | 1 | 93 |
|  | 10 | B | 2 | 0.63 | 0.31 | 3.22 | 3.45 | + | 0 | 0 |
| RO-ST22 | 1 | B | 2 | 0.63 | 0.32 | 3.22 | 2.67 | + | 1 | 93 |
|  | 2 | G | 4* | 1.08 | 0.36 | 3.23 | 1.91 | + | 1 | 92 |
|  | 3 | B | 4 | 1.14 | 0.29 | 3.23 | 1.9 | + | 1 | 89 |
|  | 4 | B | 2 | 0.61 | 0.3 | 3.24 | 3.25 | + | 1 | 94 |
|  | 5 | B | 2 | 0.63 | 0.31 | 3.24 | 2.89 | + | 0 | 0 |
|  | 6 | B | 4 | 1.13 | 0.28 | 3.24 | 2.64 | + | 1 | 95 |
|  | 7 | B | 2 | 0.62 | 0.31 | 3.25 | 2.9 | + | 1 | 94 |
|  | 8 | G | 4* | 1.06 | 0.35 | 3.25 | 0.9 | + | 1 | 90 |
|  | 9 | B | 2 | 0.62 | 0.31 | 3.25 | 3.18 | + | 1! | 94 |
|  | 10 | B | 2 | 0.63 | 0.31 | 3.25 | 2.7 | + | 0 | 0 |
| RO-ST23 | 1 | B | 2 | 0.63 | 0.31 | 3.25 | 3.25 | + | 1 | 94 |
|  | 2 | B | 2 | 0.65 | 0.33 | 3.25 | 3.65 | + | 1 | 94 |
|  | 3 | B | 2 | 0.64 | 0.32 | 3.25 | 3.26 | + | 1 | 94 |
|  | 4 | B | 2 | 0.62 | 0.31 | 3.25 | 3.26 | + | 1 | 94 |
|  | 5 | B | 2 | 0.63 | 0.32 | 3.25 | 3.26 | + | 0 | 0 |
|  | 6 | B | 2 | 0.64 | 0.32 | 3.25 | 2.08 | + | 0 | 0 |
|  | 7 | B | 2 | 0.64 | 0.32 | 3.26 | 1.96 | + | 1 | 94 |
|  | 8 | B | 2 | 0.63 | 0.32 | 3.26 | 2.63 | + | 1 | 94 |
|  | 9 | B | 2 | 0.62 | 0.31 | 3.26 | 3.36 | + | 1 | 94 |
|  | 10 | B | 2 | 0.64 | 0.32 | 3.27 | 2.66 | + | 0 | 0 |
| RO-ST24 | 1 | B | 2 | 0.64 | 0.32 | 3.27 | 2.8 | + | 0 | 0 |
|  | 2 | B | 2 | 0.63 | 0.32 | 3.27 | 3.36 | + | 0 | 0 |
|  | 3 | B | 2 | 0.66 | 0.33 | 3.28 | 3.24 | + | 0 | 0 |
|  | 4 | B | 2 | 0.62 | 0.31 | 3.28 | 3.69 | + | 1 | 94 |
|  | 5 | B | 2 | 0.66 | 0.33 | 3.28 | 3.72 | + | 0 | 0 |
|  | 6 | B | 2 | 0.62 | 0.31 | 3.28 | 4.12 | + | 0 | 0 |
|  | 7 | B | 2 | 0.62 | 0.31 | 3.28 | 2.43 | + | 1! | 94 |
|  | 8 | B | 2 | 0.63 | 0.31 | 3.29 | 3.21 | + | 0 | - |
|  | 9 | B | 2 | 0.65 | 0.33 | 3.29 | 2.97 | + | 1 | 94 |
|  | 10 | B | 2 | 0.64 | 0.32 | 3.29 | 3.62 | + | 1 | 95 |
| RO-ST25 | 1 | B | 2 | 0.64 | 0.32 | 3.3 | 3.08 | + | 1 | 95 |
|  | 2 | B | 2 | 0.63 | 0.32 | 3.3 | 2.36 | + | 1 | 95 |
|  | 3 | B | 2 | 0.64 | 0.32 | 3.3 | 3.25 | NA | 1 | 95 |
|  | 4 | B | 2 | 0.65 | 0.33 | 3.31 | 2.89 | + | 0 | 0 |
|  | 5 | B | 2 | 0.66 | 0.33 | 3.31 | 3.36 | + | 0 | 0 |
|  | 6 | B | 2 | 0.65 | 0.32 | 3.31 | 3.19 | + | 1 | 95 |
|  | 7 | B | 2 | 0.63 | 0.32 | 3.31 | 3.78 | + | 1 | 95 |
|  | 8 | B | 2 | 0.67 | 0.33 | 3.31 | 3.02 | + | 0 | 0 |
|  | 9 | B | 2 | 0.66 | 0.33 | 3.31 | 2.5 | + | 1 | 95 |
|  | 10 | B | 2 | 0.69 | 0.35 | 3.32 | 2.57 | NA | 0 | - |
| RO-ST26 | 1 | B | 2 | 0.63 | 0.32 | 3.32 | 3.32 | + | 1 | 95 |
|  | 2 | B | 2 | 0.63 | 0.32 | 3.32 | 3.72 | + | 1 | 95 |
|  | 3 | B | 2 | 0.64 | 0.32 | 3.32 | 3.25 | + | 0 | 0 |
|  | 4 | B | 2 | 0.65 | 0.32 | 3.32 | 2.6 | + | 1 | 95 |
|  | 5 | G | 4* | 1.45 | 0.35 | 3.32 | 3.52 | + | 1 | 94 |
|  | 6 | G | 4* | 1.44 | 0.35 | 3.32 | 3.47 | NA | 0 | 0 |
|  | 7 | B | 2 | 0.64 | 0.32 | 3.32 | 3.56 | + | 1 | 96 |
|  | 8 | G | 4* | 1.45 | 0.35 | 3.32 | 1.89 | NA | 1 | 83 |
|  | 9 | B | 2 | 0.66 | 0.33 | 3.32 | 3.95 | + | 1 | 96 |
|  | 10 | B | 2 | 0.63 | 0.32 | 3.33 | 3.65 | + | 1 | 96 |
| RO-ST27 | 1 | B | 2 | 0.63 | 0.31 | 3.33 | 3.66 | + | 0 | 0 |
|  | 2 | G | 4* | 1.42 | 0.34 | 3.33 | 3.84 | NA | 1 | 77 |
|  | 3 | B | 2 | 0.65 | 0.32 | 3.33 | 3.65 | + | 1 | 96 |
|  | 4 | B | 2 | 0.62 | 0.31 | 3.33 | 3.78 | + | 1 | 96 |
|  | 5 | B | 2 | 0.63 | 0.31 | 3.34 | 3.13 | + | 1 | 97 |
|  | 6 | B | 2 | 0.62 | 0.31 | 3.34 | 2.56 | + | 1 | 97 |
|  | 7 | B | 2 | 0.64 | 0.32 | 3.34 | 3.03 | NA | 1 | 97 |
|  | 8 | B | 2 | 0.64 | 0.32 | 3.35 | 3.11 | + | 0 | 0 |
|  | 9 | G | 4* | 1.45 | 0.35 | 3.35 | 3.45 | + | 1 | 94 |
|  | 10 | G | 4* | 1.47 | 0.36 | 3.35 | 4.2 | NA | 0 | 0 |
| RO-ST28 | 1 | B | 2 | 0.65 | 0.32 | 3.35 | 3.41 | + | 1 | 97 |
|  | 2 | B | 2 | 0.64 | 0.32 | 3.35 | 3.69 | + | 0 | 0 |
|  | 3 | B | 4 | 1.15 | 0.29 | 3.36 | 3.57 | NA | 1 | 82 |
|  | 4 | B | 4 | 1.16 | 0.29 | 3.36 | 3.95 | NA | 1 | 68 |
|  | 5 | B | 4 | 1.15 | 0.29 | 3.36 | 3.31 | + | 0 | - |
|  | 6 | B | 2 | 0.63 | 0.32 | 3.36 | 2.47 | + | 0 | 0 |
|  | 7 | B | 2 | 0.65 | 0.33 | 3.36 | 2.53 | NA | 1 | 97 |
|  | 8 | B | 2 | 0.65 | 0.33 | 3.37 | 3.98 | + | 1 | 97 |
|  | 9 | B | 2 | 0.66 | 0.33 | 3.37 | 3.38 | + | 1 | 97 |
|  | 10 | B | 2 | 0.65 | 0.32 | 3.37 | 3.07 | + | 1 | 97 |
| RO-ST29 | 1 | B | 4 | 1.19 | 0.3 | 3.38 | 2.06 | NA | 0 | 0 |
|  | 2 | B | 2 | 0.68 | 0.34 | 3.38 | 3.72 | + | 0 | - |
|  | 3 | B | 2 | 0.66 | 0.33 | 3.38 | 3.65 | + | 0 | 0 |
|  | 4 | B | 2 | 0.65 | 0.33 | 3.38 | 3.03 | + | 0 | 0 |
|  | 5 | B | 4 | 1.15 | 0.29 | 3.39 | 3.26 | NA | 1 | 85 |
|  | 6 | B | 4 | 1.17 | 0.29 | 3.39 | 3.69 | NA | 0 | 0 |
|  | 7 | B | 4 | 1.13 | 0.28 | 3.4 | 3.94 | + | 1 | 74 |
|  | 8 | B | 4 | 1.15 | 0.29 | 3.4 | 3.99 | + | 1 | 87 |
|  | 9 | B | 2 | 0.64 | 0.32 | 3.4 | 3.4 | NA | 1 | 98 |
|  | 10 | B | 2 | 0.64 | 0.32 | 3.41 | 2.8 | + | 1 | 98 |
| RO-ST30 | 1 | B | 2 | 0.64 | 0.32 | 3.41 | 3.77 | + | 0 | 0 |
|  | 2 | B | 2 | 0.66 | 0.33 | 3.41 | 3.56 | NA | 1 | 98 |
|  | 3 | B | 2 | 0.68 | 0.34 | 3.41 | 2.79 | NA | 0 | 0 |
|  | 4 | B | 2 | 0.64 | 0.32 | 3.42 | 3.98 | + | 1 | 99 |
|  | 5 | B | 2 | 0.65 | 0.33 | 3.42 | 3.21 | + | 0 | 0 |
|  | 6 | B | 2 | 0.64 | 0.32 | 3.42 | 2.94 | + | 0 | 0 |
|  | 7 | B | 2 | 0.65 | 0.33 | 3.43 | 2.84 | + | 1 | - |
|  | 8 | B | 2 | 0.66 | 0.33 | 3.43 | 3.24 | + | 1 | - |
|  | 9 | B | 2 | 0.63 | 0.32 | 3.43 | 2.56 | + | 1 | - |
|  | 10 | B | 4 | 1.13 | 0.28 | 3.44 | 3 | + | 1 | - |
| RO-ST31 | 1 | B | 2 | 0.63 | 0.32 | 3.44 | 3.98 | + | 0 | - |
|  | 2 | B | 2 | 0.62 | 0.31 | 3.44 | 3.86 | NA | 0 | 0 |
|  | 3 | B | 2 | 0.63 | 0.31 | 3.44 | 3.77 | + | 0 | 0 |
|  | 4 | B | 2 | 0.63 | 0.31 | 3.44 | 3.59 | + | 1 | - |
|  | 5 | B | 2 | 0.63 | 0.32 | 3.45 | 2.29 | + | 1 | - |
|  | 6 | B | 2 | 0.63 | 0.31 | 3.45 | 3.25 | NA | 1 | - |
|  | 7 | B | 2 | 0.63 | 0.31 | 3.46 | 3.54 | NA | 0 | 0 |
|  | 8 | B | 2 | 0.62 | 0.31 | 3.47 | 3.56 | + | 0 | - |
|  | 9 | B | 2 | 0.63 | 0.31 | 3.47 | 3.57 | + | 1 | - |
|  | 10 | B | 2 | 0.63 | 0.32 | 3.48 | 3.56 | + | 1 | - |
| RO-ST32 | 1 | B | 2 | 0.62 | 0.31 | 3.48 | 3.99 | NA | 0 | 0 |
|  | 2 | B | 2 | 0.62 | 0.31 | 3.48 | 3.25 | NA | 1 | - |
|  | 3 | B | 2 | 0.62 | 0.31 | 3.48 | 3.95 | + | 1 | - |
|  | 4 | B | 2 | 0.63 | 0.31 | 3.49 | 3.26 | + | 0 | 0 |
|  | 5 | B | 2 | 0.64 | 0.32 | 3.51 | 2.69 | + | 1 | - |
|  | 6 | B | 4 | 1.17 | 0.29 | 3.51 | 3.66 | + | 1 | 72 |
|  | 7 | B | 4 | 1.16 | 0.29 | 3.52 | 3.07 | + | 1 | 66 |
|  | 8 | B | 4 | 1.13 | 0.28 | 3.52 | 1.8 | + | 1 | 59 |
|  | 9 | B | 2 | 0.62 | 0.31 | 3.52 | 3.99 | + | 1 | - |
|  | 10 | B | 2 | 0.63 | 0.32 | 3.52 | 2.7 | + | 1 | - |
| RO-ST33 | 1 | B | 2 | 0.62 | 0.31 | 3.54 | 3.69 | + | 1 | - |
|  | 2 | B | 2 | 0.63 | 0.32 | 3.54 | 4.19 | + | 0 | 0 |
|  | 3 | B | 2 | 0.62 | 0.31 | 3.55 | 2.52 | + | 1 | - |
|  | 4 | B | 2 | 0.62 | 0.31 | 3.55 | 3.26 | + | 0 | 0 |
|  | 5 | B | 4 | 1.15 | 0.29 | 3.55 | 3.75 | + | 1 | 61 |
|  | 6 | B | 2 | 0.63 | 0.32 | 3.56 | 3.42 | + | 0 | 0 |
|  | 7 | B | 4 | 1.15 | 0.29 | 3.56 | 2.12 | + | 1 | 84 |
|  | 8 | B | 2 | 0.63 | 0.32 | 3.56 | 3.01 | + | 0 | 0 |
|  | 9 | B | 2 | 0.62 | 0.31 | 3.56 | 3.78 | + | 0 | 0 |
|  | 10 | B | 2 | 0.62 | 0.31 | 3.58 | 3.99 | + | 1 | - |
| RO-ST34 | 1 | B | 2 | 0.66 | 0.33 | 3.58 | 3.65 | NA | 0 | 0 |
|  | 2 | B | 2 | 0.64 | 0.32 | 3.58 | 3.09 | + | 1 | - |
|  | 3 | B | 2 | 0.64 | 0.32 | 3.59 | 3.62 | NA | 0 | 0 |
|  | 4 | B | 2 | 0.63 | 0.32 | 3.6 | 3.98 | + | 1 | - |
|  | 5 | B | 2 | 0.64 | 0.32 | 3.6 | 3.94 | NA | 1 | - |
|  | 6 | B | 2 | 0.63 | 0.31 | 3.6 | 3.94 | NA | 1 | - |
|  | 8 | B | 2 | 0.62 | 0.31 | 3.6 | 4.1 | NA | 0 | 0 |
|  | 9 | B | 2 | 0.61 | 0.31 | 3.6 | 2.79 | NA | 1 | - |
|  | 10 | B | 2 | 0.63 | 0.32 | 3.62 | 2.77 | + | 1 | - |
| RO-ST35 | 1 | B | 2 | 0.61 | 0.31 | 3.63 | 3.39 | + | 1 | - |
|  | 2 | B | 2 | 0.62 | 0.31 | 3.63 | 3.69 | NA | 0 | - |
|  | 3 | B | 2 | 0.62 | 0.31 | 3.64 | 3.57 | + | 1 | - |
|  | 4 | B | 2 | 0.61 | 0.31 | 3.65 | 3.34 | + | 1 | - |
|  | 5 | B | 2 | 0.64 | 0.32 | 3.65 | 3.32 | + | 0 | 0 |
|  | 6 | B | 2 | 0.62 | 0.31 | 3.67 | 3.31 | + | 0 | 0 |
|  | 7 | B | 2 | 0.63 | 0.31 | 3.67 | 3.58 | + | 1 | - |
|  | 8 | B | 2 | 0.64 | 0.32 | 3.67 | 2.85 | + | 0 | - |
|  | 9 | B | 2 | 0.62 | 0.31 | 3.67 | 2.85 | + | 1 | - |
|  | 10 | B | 2 | 0.62 | 0.31 | 3.69 | 3.02 | + | 0 | 0 |
| RO-ST36 | 1 | B | 4 | 1.15 | 0.29 | 3.69 | 1.85 | NA | 0 | 0 |
|  | 2 | B | 2 | 0.62 | 0.31 | 3.69 | 2.74 | NA | 0 | 0 |
|  | 3 | B | 2 | 0.59 | 0.3 | 3.7 | 2.56 | NA | 1 | - |
|  | 4 | G | 4* | 1.46 | 0.35 | 3.7 | 3.69 | NA | 1 | 78 |
|  | 5 | G | 4* | 1.46 | 0.35 | 3.7 | 2.62 | NA | 0 | 0 |
|  | 6 | G | 4* | 1.48 | 0.36 | 3.71 | 1.72 | NA | 0 | 0 |
|  | 7 | B | 4 | 1.12 | 0.28 | 3.71 | 1.73 | NA | 0 | 0 |
|  | 8 | G | 4* | 1.46 | 0.35 | 3.71 | 2.52 | NA | 1 | 79 |
|  | 9 | G | 4* | 1.47 | 0.35 | 3.72 | 3.13 | + | 1 | 67 |
|  | 10 | B | 4 | 1.18 | 0.3 | 3.72 | 3.86 | NA | 0 | 0 |
| RO-ST37 | 1 | B | 2 | 0.63 | 0.31 | 3.72 | 3.97 | + | 1 | - |
|  | 2 | B | 2 | 0.62 | 0.31 | 3.72 | 3.09 | + | 1 | - |
|  | 3 | B | 4 | 1.14 | 0.28 | 3.73 | 3.56 | + | 0 | 0 |
|  | 4 | B | 2 | 0.62 | 0.31 | 3.73 | 2.83 | + | 1 | - |
|  | 5 | B | 4 | 1.14 | 0.29 | 3.74 | 1.71 | + | 0 | 0 |
|  | 6 | B | 2 | 0.63 | 0.31 | 3.75 | 3.96 | + | 0 | 0 |
|  | 7 | B | 2 | 0.63 | 0.32 | 3.75 | 2.97 | + | 1 | - |
|  | 8 | B | 2 | 0.62 | 0.31 | 3.76 | 3.36 | NA | 0 | 0 |
|  | 9 | B | 2 | 0.63 | 0.31 | 3.76 | 3.16 | NA | 0 | 0 |
|  | 10 | B | 4 | 1.15 | 0.29 | 3.78 | 2.17 | + | 1 | 71 |
| RO-ST38 | 1 | B | 4 | 1.18 | 0.3 | 3.78 | 3.56 | + | 1 | 91 |
|  | 2 | B | 4 | 1.19 | 0.3 | 3.79 | 3.5 | + | 1 | 75 |
|  | 3 | B | 4 | 1.14 | 0.28 | 3.8 | 3.03 | NA | 1 | - |
|  | 4 | B | 4 | 1.14 | 0.29 | 3.8 | 3.25 | + | 1 | 69 |
|  | 5 | B | 4 | 1.16 | 0.29 | 3.81 | 2.84 | + | 1 | 61 |
|  | 6 | B | 4 | 1.17 | 0.29 | 3.82 | 3.58 | + | 1 | 77 |
|  | 7 | B | 4 | 1.16 | 0.29 | 3.82 | 2.77 | + | 1 | 81 |
|  | 8 | B | 4 | 1.17 | 0.29 | 3.82 | 1.77 | + | 1 | 73 |
|  | 9 | B | 4 | 1.14 | 0.28 | 3.83 | 2.7 | + | 1 | 56 |
|  | 10 | B | 4 | 1.14 | 0.29 | 3.83 | 3.6 | + | 1 | 68 |
| RO-ST39 | 1 | B | 2 | 0.67 | 0.31 | 3.83 | 2.61 | NA | 0 | 0 |
|  | 2 | B | 2 | 0.65 | 0.31 | 3.84 | 2.69 | + | 1 | - |
|  | 3 | B | 4 | 0.67 | 0.3 | 3.85 | 2.65 | NA | 0 | 0 |
|  | 4 | B | 2 | 0.65 | 0.32 | 3.85 | 2.66 | NA | 1! | - |
|  | 5 | B | 2 | 0.65 | 0.32 | 3.87 | 2.9 | + | 0 | 0 |
|  | 6 | B | 2 | 0.62 | 0.3 | 3.88 | 2.99 | + | 0 | 0 |
|  | 7 | B | 2 | 0.66 | 0.31 | 3.88 | 2.71 | + | 0 | 0 |
|  | 8 | B | 2 | 0.66 | 0.31 | 3.88 | 3.6 | + | 1 | - |
|  | 10 | B | 2 | 0.67 | 0.3 | 3.9 | 3.55 | + | 1 | - |
| RO-ST40 | 1 | B | 2 | 0.63 | 0.33 | 3.91 | 3.85 | + | 0 | 0 |
|  | 2 | B | 2 | 0.62 | 0.32 | 3.91 | 2.63 | NA | 0 | 0 |
|  | 3 | B | 2 | 1.25 | 0.33 | 3.92 | 3.45 | + | 0 | 0 |
|  | 4 | B | 2 | 0.63 | 0.32 | 3.92 | 3.86 | NA | 0 | 0 |
|  | 5 | B | 2 | 0.64 | 0.32 | 3.94 | 3.27 | + | 0 | 0 |
|  | 6 | B | 2 | 0.61 | 0.31 | 3.94 | 4.4 | NA | 0 | 0 |
|  | 7 | B | 2 | 0.62 | 0.33 | 3.94 | 3.29 | + | 1 | - |
|  | 8 | B | 2 | 0.63 | 0.33 | 3.96 | 3.55 | + | 1 | - |
|  | 9 | B | 2 | 0.65 | 0.33 | 3.96 | 3.08 | + | 0 | 0 |
|  | 10 | B | 2 | 0.61 | 0.34 | 3.96 | 4.08 | + | 1 | - |
| RO-ST41 | 1 | B | 2 | 0.61 | 0.3 | 3.97 | 4.55 | + | 0 | 0 |
|  | 2 | B | 2 | 0.61 | 0.31 | 3.97 | 3.94 | + | 1 | - |
|  | 3 | B | 2 | 0.6 | 0.3 | 3.97 | 4.22 | + | 0 | 0 |
|  | 4 | B | 2 | 0.61 | 0.3 | 3.99 | 4.38 | + | 0 | 0 |
|  | 5 | B | 2 | 0.6 | 0.3 | 4.06 | 4.37 | + | 0 | 0 |
|  | 6 | B | 2 | 0.6 | 0.3 | 4.06 | 3.59 | + | 0 | 0 |
|  | 7 | B | 2 | 0.6 | 0.3 | 4.14 | 3.65 | NA | 0 | - |
|  | 8 | B | 2 | 0.62 | 0.31 | 4.2 | 4.27 | + | 1 | - |
|  | 10 | B | 4 | 1.13 | 0.28 | 4.74 | 1.76 | + | 1 | 82 |

**SUPPLEMENTARY TABLE S3** The list of eight quantitative morphological traits used in the morphometric analyses

| **Character code** | **Character explanation** |
| --- | --- |
| LP | the maximum length of the petal (mm) |
| WP | the maximum width of the petal (mm) |
| DPI | the depth of the petal incision (mm) |
| LST | the maximum length of the style (mm) |
| LS | the maximum length of the sepal (mm) |
| WS | the maximum width of the sepal (mm) |
| WMM | the maximum width of the membranous sepal margin (mm) |
| LBS | the length from the base to the widest part of sepal (mm) |

**SUPPLEMENTARY TABLE S4** Summary of GLMMs testing for the effect of cytotype, sex and their interaction on eight measured floral morphological traits. Likelihood-ratio test statistics (χ2) and associated probabilities (p) are displayed for each term and whole models, respectively. Marginal (R2m) and conditional determination coefficients (R2c) are given for each model. The floral morphological trait abbreviations are as follows: LP - the maximum length of the petal; WP - the maximum width of the petal; DPI - the depth of the petal incision; LST - the maximum length of the style; LS - the maximum length of the sepal; WS - the maximum width of the sepal; WMM - the maximum width of the membranous sepal margin and LBS - the length from the base to the widest part of sepal.

|  | Cytotype | |  | Sex | |  | Cytotype × Sex | |  | Whole model | | |
| --- | --- | --- | --- | --- | --- | --- | --- | --- | --- | --- | --- | --- |
| Variable | χ^2^ | p |  | χ^2^ | p |  | χ^2^ | p |  | χ^2^ | p | R^2^_m_/R^2^_c_ |
| LP | 33.0 | < 0.0001 |  | 532.6 | < 0.0001 |  | 8.3 | **0.0040** |  | 577.2 | < 0.0001 | 0.06/0.14 |
| WP | 33.6 | < 0.0001 |  | 106.7 | < 0.0001 |  | 4.7 | **0.0302** |  | 148.7 | < 0.0001 | 0.16/0.52 |
| DPI | 12.6 | 0.0004 |  | 55.5 | < 0.0001 |  | 0.4 | 0.5182 |  | 70.7 | < 0.0001 | 0.21/0.80 |
| LST | 13.5 | 0.0002 |  | 110.0 | < 0.0001 |  | 5.2 | **0.0229** |  | 123.6 | < 0.0001 | 0.05/0.16 |
| LS | 51.9 | < 0.0001 |  | 15.5 | 0.0001 |  | 0.4 | 0.5084 |  | 70.4 | < 0.0001 | 0.03/0.14 |
| WS | 33.8 | < 0.0001 |  | 7.8 | 0.0051 |  | 2.1 | 0.1487 |  | 41.7 | < 0.0001 | 0.09/0.49 |
| WMM | 14.4 | 0.0001 |  | 5.6 | 0.0184 |  | 2.5 | 0.1113 |  | 23.4 | < 0.0001 | 0.16/0.94 |
| LBS | 10.4 | 0.0013 |  | 9.8 | 0.0017 |  | 0.7 | 0.4146 |  | 22.0 | 0.0001 | 0.03/0.30 |

**SUPPLEMENTARY TABLE S5** The list of ecological variables used for estimation of the effects of habitat quality on the frequency of occurrence of sexual morphs and cytotypes in populations of *S. graminea*.
